# Genomic prediction of blood biomarkers of metabolic disorders in Holstein cattle using parametric and nonparametric models

**DOI:** 10.1186/s12711-024-00903-9

**Published:** 2024-04-29

**Authors:** Lucio F. M. Mota, Diana Giannuzzi, Sara Pegolo, Enrico Sturaro, Daniel Gianola, Riccardo Negrini, Erminio Trevisi, Paolo Ajmone Marsan, Alessio Cecchinato

**Affiliations:** 1 Department of Agronomy, Food, Natural Resources, Animals and Environment (DAFNAE), University of Padova, 35020 Legnaro, PD Italy; 2 Department of Animal Science, Food and Nutrition (DIANA) and the Romeo and Enrica Invernizzi Research Center for Sustainable Dairy Production (CREI), Faculty of Agricultural, Food, and Environmental Sciences, Università Cattolica del Sacro Cuore, 29122 Piacenza, Italy; 3 Nutrigenomics and Proteomics Research Center, Università Cattolica del Sacro Cuore, 29122 Piacenza, Italy; 4 Department of Animal and Dairy Sciences, University of Wisconsin, Madison, WI 53706 USA

## Abstract

**Background:**

Metabolic disturbances adversely impact productive and reproductive performance of dairy cattle due to changes in endocrine status and immune function, which increase the risk of disease. This may occur in the post-partum phase, but also throughout lactation, with sub-clinical symptoms. Recently, increased attention has been directed towards improved health and resilience in dairy cattle, and genomic selection (GS) could be a helpful tool for selecting animals that are more resilient to metabolic disturbances throughout lactation. Hence, we evaluated the genomic prediction of serum biomarkers levels for metabolic distress in 1353 Holsteins genotyped with the 100K single nucleotide polymorphism (SNP) chip assay. The GS was evaluated using parametric models best linear unbiased prediction (GBLUP), Bayesian B (BayesB), elastic net (ENET), and nonparametric models, gradient boosting machine (GBM) and stacking ensemble (Stack), which combines ENET and GBM approaches.

**Results:**

The results show that the Stack approach outperformed other methods with a relative difference (RD), calculated as an increment in prediction accuracy, of approximately 18.0% compared to GBLUP, 12.6% compared to BayesB, 8.7% compared to ENET, and 4.4% compared to GBM. The highest RD in prediction accuracy between other models with respect to GBLUP was observed for haptoglobin (hapto) from 17.7% for BayesB to 41.2% for Stack; for Zn from 9.8% (BayesB) to 29.3% (Stack); for ceruloplasmin (CuCp) from 9.3% (BayesB) to 27.9% (Stack); for ferric reducing antioxidant power (FRAP) from 8.0% (BayesB) to 40.0% (Stack); and for total protein (PROTt) from 5.7% (BayesB) to 22.9% (Stack). Using a subset of top SNPs (1.5k) selected from the GBM approach improved the accuracy for GBLUP from 1.8 to 76.5%. However, for the other models reductions in prediction accuracy of 4.8% for ENET (average of 10 traits), 5.9% for GBM (average of 21 traits), and 6.6% for Stack (average of 16 traits) were observed.

**Conclusions:**

Our results indicate that the Stack approach was more accurate in predicting metabolic disturbances than GBLUP, BayesB, ENET, and GBM and seemed to be competitive for predicting complex phenotypes with various degrees of mode of inheritance, i.e. additive and non-additive effects. Selecting markers based on GBM improved accuracy of GBLUP.

**Supplementary Information:**

The online version contains supplementary material available at 10.1186/s12711-024-00903-9.

## Background

Dairy cows may experience stressful periods during lactation, which are mainly caused by the high energy demands required for milk production [1, 2]. During this period, changes in metabolic and endocrine status occur, which can lead to increased lipolysis and proteolysis in support of a high milk yield [3, 4]. When metabolic stress occurs in lactating dairy cows, it triggers increased levels of haptoglobin, non-esterified fatty acids (NEFA), β-hydroxybutyrate acid (BHBA), ceruloplasmin (CuCp), and globulins in their serum and decreased levels of paraoxonase (PON), glucose, and albumin [5]. In this context, increased attention has been directed towards mitigating the harmful effects of metabolic imbalances, such as alterations in energy, immune, and hormonal states, with the aim of improving health and resilience of dairy cows [6].

Metabolic stress disturbances, such as ketosis, milk fever, and metabolic energy imbalances, are directly related to changes in the levels of NEFA, BHBA, and glucose and increase the chance of harmful effects on animal health and welfare, thus affecting farm profitability [7]. Assessing subclinical metabolic disorders through blood metabolite analyses can help to differentiate between cows that are prone to developing metabolic disorders and resilient cows. Studies of metabolic health disorders in dairy cattle that aim at selecting resilient animals and investigating responses to stressors have been recently performed [8]. Several biomarkers have been proposed for the detection of metabolic imbalance, such as metabolites associated with oxidative stress (reactive oxygen metabolites and species), energy balance (BHB, glucose, and NEFA), mineral status (calcium [Ca], sodium [Na], and magnesium [Mg]), liver function status (aspartate aminotransferase [AST], γ-glutamyl transferase [GGT], urea and albumin), and innate immunity and inflammation status (globulins, haptoglobin [hapto], myeloperoxidase, and ceruloplasmin [CuCp]) [9–11].

Traditionally, the effects of metabolic disorders have been mainly evaluated in the most critical phase of the lactation period, i.e. early lactation [12, 13]. However, recent studies have highlighted that changes in the immune and metabolic aspects of dairy cattle can occur in late lactation and in the dry-off period with subclinical forms, which can have, in turn, long-term effects on the subsequent lactation [14, 15]. In this view, serum biomarkers of inflammation, oxidative stress or hepatic overload could unveil crucial information on the health status of the cows throughout the lactation period, and deepen the knowledge on the complex mechanisms leading to post-partum metabolic imbalances. This knowledge of the critical levels for blood metabolites can help establish efficient nutrition and management strategies for preventive control of metabolic distress. Although these practices do not guarantee the complete prevention of clinical or subclinical metabolic disorders, genetic/genomic selection may represent a complementary approach to increase resilience in dairy cattle herds.

Exploring genetic markers (i.e., single nucleotide polymorphisms—SNPs) in genomic prediction (GP) approaches as predictors of complex phenotypes for breeding purposes is of interest. SNP-based approaches can help elucidate the genetic background of traits and enhance prediction over what is possible with pedigree-based approaches (i.e., best linear unbiased prediction—BLUP) [16]. Most GP approaches assume that observed phenotypes come from the action of several loci with additive effects distributed across the whole genome and produce shrinkage of estimated effect sizes, often leading to better predictive performance [17]. However, the inheritance of blood metabolites ranges from simple to complex, i.e., from a few to many underlying quantitative trait loci (QTL) [10, 11, 18]. In addition, non-additive effects (dominance or epistasis) could affect blood metabolites, thus the statistical approaches that capture non-additive inheritance might contribute to improve GP accuracy [19, 20]. The optimal direction of selection for most blood metabolic stress biomarkers is still under discussion. Indeed, many metabolites have level thresholds, which can allow the identification of cows at risk of compromising performance and health. Although these thresholds are not yet well-defined, several studies are being conducted to establish these subclinical thresholds [21, 22]. Moreover, animals with suboptimal or critical values can be found at both the extremes of the metabolite distribution (e.g., urea and total proteins), which complicates selective breeding programs.

In recent years, various machine learning (ML) methods have been employed for GP, providing opportunities to accommodate complex genetic architectures more efficiently than parametric models using less rigid assumptions [23–25]. Several studies have noted that ML, such as random forest (RF), support vector regression (SVR), gradient boosting machine (GBM), artificial neural networks (ANN), and stacking ensemble learning, can be used for GP, and outperform parametric models (i.e., GBLUP and Bayesian regression) in situations where the reference population is small [26–29]. On the one hand, ML techniques have also been explored as a potential strategy for variable selection and then used in genomic BLUP (GBLUP) [30, 31]. On the other hand, Azodi et al. [32], with data on plants, and Bellot et al. [33], with human data, found that some ML models (e.g., deep neural networks) had a lower stability (i.e., show variation in prediction accuracy when trained on different validation designs or populations) than linear models. To date, information on the application of GP approaches for predicting blood metabolites in dairy cows is scarce. Hence, this study was carried out to compare the predictive ability of GBLUP against Bayesian (BayesB), two machine learning approaches (GBM and stacking ensemble learning), and penalized regression via the elastic net in dairy cattle. The target phenotypes were blood metabolites related to energy, liver function and hepatic damage, oxidative stress, inflammation and innate immunity, and minerals.

## Methods

### Field data

The dataset comes from the BENELAT project, which aims at creating strategies and approaches to mitigate stressful factors to improve animal welfare and milk components in dairy cattle production systems [34]. Cows were fed twice daily with a ration based on corn silage and sorghum, and energy-protein supplementation was formulated following the nutritional recommendations for dairy cattle [35]. Blood samples were obtained from 1367 Holstein cows belonging to five herds in northern Italy (Emilia-Romagna and Veneto region) under a similar dairy production system. Cows were subjected to a health examination before blood sampling, and animals with clinical diseases or receiving medical treatment were excluded from the study. The animal handling procedure used followed the ARRIVE (Animal Research: Reporting of In Vivo Experiments) guidelines and was approved by the ethical committee Organismo Preposto al Benessere degli Animali (OPBA; Organization for Animal Welfare) of the Università Cattolica del Sacro Cuore (Piacenza, Italy) and by the Italian Ministry of Health (protocol number 510/2019-PR of 19/07/2019).

### Blood sampling

Blood samples were collected in 21 batches (i.e., herd/date combinations): 16 batches in 2019 (1013 cows) and five batches in 2020 (354 cows). The average values (± standard deviation (SD)) were 33.38 ± 9.11 for daily milk yield (kg), 3.71 ± 0.88 for fat (%), and 3.43 ± 0.36 for protein (%). The cows had an average for days in milk (DIM) of 191.32 ± 110.30 and an average number of parities of 2.09 ± 1.25. Five mL of blood from each cow were collected after the morning milking and before feeding through jugular venipuncture using vacutainer tubes containing 150 USP units of lithium heparin as anticoagulant (Vacumed; FL Medical, Torreglia, Padua, Italy). All blood samples were maintained on ice until two hours after blood sampling, followed by centrifugation at 3500 × g for 1 min at 6 °C (Hettich Universal 16R Centrifuge), and then the plasma samples obtained were collected and stored at – 20 °C until assessment of blood metabolites.

### Genomic data

In total, 1365 Holstein cows were genotyped with the Geneseek Genomic Profiler (GGP) Bovine 100 k SNP Chip assay. After removing the non-autosomal regions, we carried out genotype quality control. Autosomal SNPs with a minor allele frequency (MAF) lower than 0.05 and a significant deviation from Hardy–Weinberg equilibrium (*P* ≤ 10^−5^) were removed, as well as SNPs and samples with call rate lower than 0.95. After quality control, 1353 cows with phenotypic information and genotyped with 61,226 SNPs remained for genomic analyses. Principal component analysis (PCA) was used to assess population substructure based on the SNPs using the ade4 R package [36], and two distinct groups were clustered using k-means clustering (see Additional file 1: Fig. S1).

### Blood metabolic profile

In total, 28 blood metabolites were analyzed using a clinical autoanalyzer (ILAB 650, Instrumentation Laboratory, Lexington, MA) following the method reported by Calamari et al. [37] and Hanasand et al*.* [38]. A complete metabolic profile that includes energy-related metabolites (glucose, cholesterol, NEFA, BHB, urea, and creatinine), liver function/hepatic damage (AST, GGT, total bilirubin [BILt], albumin, alkaline phosphatase [ALP], and paraoxonase [PON]), oxidative stress (total reactive oxygen metabolites [ROMt]; advanced oxidation protein products [AOPP]; ferric reducing antioxidant power [FRAP]; thiol groups [SHp]), inflammation/innate immunity (CuCp, total proteins [PROTt], globulins, hapto, and myeloperoxidase), and minerals (Ca, P, Mg, Na, K, Cl and Zn). Glucose, total proteins, albumin, hapto, urea, Ca, AST, and GGT were determined using kits purchased from Instrumentation Laboratory (IL Test). Globulin concentration was estimated as the difference between total proteins and albumin. The potassium electrolyte (K^+^) was assessed using the potentiometer method (ion-selective electrode coupled to ILAB 600). Zn, NEFA, BHB, and CuCp were analyzed using the methods reported by Calamari et al. [37]. The concentrations of AOPP, ROMt, FRAP, and PON were determined according to Premi et al. [39]. Descriptive statistics for the blood metabolites and the density plot for each blood metabolite are shown in Additional file 2: Table S1 and Additional file 1: Figs. S2, S3 and S4.

### Genetic parameters

Genetic variance components and heritability estimates for blood metabolites were obtained with the following single-trait animal model via the Bayesian approach with Gibbs sampling:

$$\mathbf{y}=\mathbf{X}\mathbf{b}+\mathbf{W}\mathbf{h}+\mathbf{Z}\mathbf{a}+\mathbf{e},$$

where $\mathbf{y}$ is a vector of blood metabolite values; $\mathbf{b}$ is the vector of fixed effects of days in milk with six levels (levels 1: less than 60 days; 2: 60–120 days; 3: 121–180 days; 4: 181–240 days; 5: 241–300 days and 6: more than 300 days) and parity in four classes (1, 2, 3, and ≥ 4 parities), with the two first principal components that explained 12.3% of the genotypic variability considered as covariables. Furthermore, $\mathbf{h}$ is the vector of the random effects of batch; $\mathbf{a}$ is the vector of additive genetic effects; $\mathbf{X}$, $\mathbf{W}$, and $\mathbf{Z}$ are incidence matrices relating $\mathbf{y}$ to fixed effects ($\mathbf{b}$), batch effects ($\mathbf{h}$), and the additive genomic breeding value ($\mathbf{a}$), respectively; and $\mathbf{e}$ is the vector of random residual effects.

The model was fitted under the following assumptions for random effects: $\mathbf{a}\sim {\text{N}}(\mathbf{0},\mathbf{G}{\upsigma }_{{\text{a}}}^{2})$, $\mathbf{h}\sim {\text{N}}(\mathbf{0},\mathbf{I}{\upsigma }_{{\text{batch}}}^{2})$ and $\mathbf{e}\sim {\text{N}}(\mathbf{0},\mathbf{I}{\upsigma }_{{\text{e}}}^{2})$, where ${\upsigma}_{\mathrm{a }}^{2}$, ${\upsigma}_{{\text{batch}}}^{2}$, and ${\upsigma}_{{\text{e}}}^{2}$ are variances for additive, batch and residual effects, respectively; $\mathbf{I}$ is an identity matrix; and $\mathbf{G}$ was obtained according to VanRaden [16]: $\mathbf{G}=\frac{\mathbf{M}{\mathbf{M}}^{\mathbf{^{\prime}}}}{2\sum_{{\text{j}}=1}^{{\text{m}}}{{\text{p}}}_{{\text{j}}}\left(1-{{\text{p}}}_{{\text{j}}}\right)}$ where $\mathbf{M}$ is the SNP matrix with codes 0, 1, and 2 for genotypes *AA, AB*, and *BB,* adjusted for allele frequency, and ${{\text{p}}}_{{\text{j}}}$ is the frequency of the second allele of the ${\text{j}}$-*th* SNP. All random effects were assumed to be mutually independent. We assigned a flat prior distribution to the fixed effects and a scaled inverse chi-square distribution as prior for the random effects. Heritability (${{\text{h}}}^{2}$) was estimated from the posterior variance components for each trait as ${{\text{h}}}^{2}={\upsigma }_{{\text{a}}}^{2}/({\upsigma }_{{\text{a}}}^{2}+{\upsigma }_{{\text{batch}}}^{2}+{\upsigma }_{{\text{e}}}^{2})$, and ${{\text{h}}}_{{\text{batch}}}^{2}={\upsigma }_{\mathrm{batch }}^{2}/({\upsigma }_{{\text{a}}}^{2}+{\upsigma }_{{\text{batch}}}^{2}+{\upsigma }_{{\text{e}}}^{2})$ was the relative contribution of the batch to the variance.

The model was implemented using the gibbsf90 + software from the blupf90 suite [40]. The Gibbs sampler comprised a chain of 500,000 cycles, with a burn-in of the first 50,000 iterations and samples stored every ten cycles. Hence, the posterior means of genetic parameters were estimated from 45,000 samples. Convergence was evaluated through visual inspection of the trace plot using the BOA package in R [41], and all traits converged (p-value > 0.10) according to the Geweke test [42].

The PREDICTf90 software [40] was used to obtain the phenotypes adjusted for the fixed and batch effects (${\mathbf{y}}^{\mathbf{*}}=\mathbf{y}-\mathbf{X}\widehat{\mathbf{b}}-\mathbf{W}\widehat{\mathbf{h}}$), and the adjusted phenotypes were used as the response variables in genomic predictions.

A model considering dominance and additive-by-additive epistatic effects was fitted to assess the dominance and epistatic genetic contribution for blood metabolic profiles as well, using the following model:

$$\mathbf{y}=\mathbf{X}\mathbf{b}+\mathbf{W}\mathbf{h}+ \mathbf{Z}\mathbf{a}+\mathbf{Z}\mathbf{d}+\mathbf{Z}{\mathbf{e}\mathbf{p}}_{\mathbf{a}\mathbf{a}}+\mathbf{e},$$

where $\mathbf{d}$ is a vector of dominance genomic effects, ${\mathbf{e}\mathbf{p}}_{\mathbf{a}\mathbf{a}}$ is a vector of random additive-by-additive genomic effects, the dominance matrix (${\mathbf{D}}$) was computed according to Vitezica et al. [43] and the additive-by-additive matrix (${\mathbf{GG}}$) was computed using Hadamard products, following the assumptions $\mathbf{d}\sim\text{ N}\left({{0}, }{\mathbf{D}}{\sigma}_{{\text{d}}}^{2}\right)$ and ${\mathbf{e}\mathbf{p}}_{\mathbf{a}\mathbf{a}}\sim\text{ N}\left({{0}, }{\mathbf{GG}}{\upsigma}_{{{\text{ep}}}_{{\text{aa}}}}^{2}\right)$ where ${\upsigma}_{{\text{d}}}^{2}$ and ${\upsigma}_{{{\text{ep}}}_{{\text{aa}}}}^{2}$ are the dominance and epistasis variance, respectively. Dominance (${{\text{d}}}^{2}$) and additive-by-additive epistasis (${{\text{ep}}}_{{\text{aa}}}^{2})$ contributions were estimated as the proportion of phenotypic variance as ${{\text{d}}}^{2}={\upsigma }_{{\text{d}}}^{2}/({\upsigma }_{{\text{a}}}^{2}+ {\upsigma }_{{\text{d}}}^{2}+{\upsigma }_{{{\text{ep}}}_{{\text{aa}}}}^{2}+{\upsigma }_{{\text{e}}}^{2})$ and ${{\text{ep}}}_{{\text{aa}}}^{2}={\upsigma }_{{{\text{ep}}}_{{\text{aa}}}}^{2}/({\upsigma }_{{\text{a}}}^{2}+ {\upsigma }_{{\text{d}}}^{2}+{\upsigma }_{{{\text{ep}}}_{{\text{aa}}}}^{2}+{\upsigma }_{{\text{e}}}^{2})$. The model was implemented using the R package BGLR version 1.09 [44], considering a Gibbs chain of 500,000 cycles, with a burn-in of the first 50,000 iterations and samples stored every ten cycles. Convergence was evaluated through visual inspection of the trace plot using the BOA package in R [41], and all traits converged (p-value > 0.15) according to the Geweke test [42].

### Cross-validation scenarios

A tenfold cross-validation (CV) scheme was used for estimating the prediction accuracies of the parametric and nonparametric approaches. First, we split the dataset into ten non-overlapping folds of approximately equal size (135 or 136 cows per fold) based on the genomic distance to reduce the dependence between training and validation sets (see Additional file 1: Fig. S1a). Thus, nine folds were assigned to train the models and one to validate the model. Then, this CV procedure was repeated ten times, predicting each validation set once. In addition, a batch-out CV scenario was performed, where the data were randomly split considering 80% of batches (i.e. 16 batches) assigned as training set and 20% of batches (i.e. 5 batches) assigned as validation set. Then, this CV scenario was repeated five times, predicting each validation set once.

### Genomic prediction (GP) analyses

#### Parametric methods

***GBLUP.*** Genomic prediction of blood metabolites used in the single-trait model:

$${\mathbf{y}}^{*}=\mathbf{1}\upmu +\mathbf{Z}\mathbf{g}+\mathbf{e},$$

where ${\mathbf{y}}^{\mathbf{*}}$ is the vector of adjusted phenotypic values for blood metabolite-related traits*,* $\mathbf{1}$ is a vector of ones, $\upmu$ is the unknown average value, $\mathbf{Z}$ is the incidence matrix for genomic estimated breeding values (GEBV); $\mathbf{g}$ is a vector of random genomic values, assumed to follow a normal distribution given by ${\text{N}}(\mathbf{0},\mathbf{G}{\upsigma }_{{\text{g}}}^{2}$), where ${\upsigma }_{{\text{g}}}^{2}$ is the genomic variance and $\mathbf{G}$ is the additive genomic relationship matrix. The vector of the residual effects ($\mathbf{e}$) was distributed as ${\text{N}}(\mathbf{0},\mathbf{I}{\upsigma }_{{\text{e}}}^{2}$), where $\mathbf{I}$ is an identity matrix and ${\upsigma }_{{\text{e}}}^{2}$ is the residual variance. The GBLUP analyses were performed using the BLUPF90 program [40].

***BayesB.*** BayesB uses a linear model with a prior on marker effects that permits variable selection [45]. In the BayesB method, it is assumed that a known portion of the SNPs do not contribute to the genetic variation of the trait, via a mixture prior distribution, where a subset of the SNPs has a null effect (i.e., a point mass at zero) with probability π or an effect that follows a univariate t-distribution with probability 1–π [17, 45]. The BayesB model for the ith individual was ${{\text{y}}}_{{\text{i}}}^{*}=\upmu +\sum_{{\text{w}}=1}^{{\text{p}}}{{\text{x}}}_{{\text{iw}}}{{\text{u}}}_{{\text{w}}}+{{\text{e}}}_{{\text{i}}}$, where ${{\text{y}}}_{{\text{i}}}^{*}$ is the adjusted phenotype, $\upmu$ is the unknown average; ${{\text{x}}}_{{\text{iw}}}$ is the SNP $w$ (coded as 0, 1, and 2) in animal ${\text{i}}$; ${{\text{u}}}_{{\text{w}}}$ is the (additive) effect of SNP $w$ (p = 61,226); and ${{\text{e}}}_{{\text{i}}}$ is a residual effect assumed to be normally distributed as $\mathbf{e}\sim N(\mathbf{0},{\mathbf{I}\upsigma }_{{\text{e}}}^{2}$). A priori, the distribution of ${{\text{u}}}_{{\text{w}}}$ is:

$${\text{p}}\left({{\text{u}}}_{{\text{w}}}|{\text{df}},\uppi ,{{\text{S}}}_{{\text{B}}}\right)=\uppi *\left({{\text{u}}}_{{\text{w}}}=0\right)+\left(1-\uppi \right)*{\text{t}}\left({{\text{u}}}_{{\text{w}}}|{\text{df}},{{\text{S}}}_{{\text{B}}}\right),$$

where $\uppi$ is the known prior probability of the SNP having a null effect, $1-\uppi$ is the probability of the SNP having a non-null effect, and ${\text{t}}\left({{\text{u}}}_{{\text{w}}}|{\text{df}},{{\text{S}}}_{{\text{B}}}\right)$ is a scaled t distribution with 5 degrees of freedom (${\text{df}}$) and scale parameter ${{\text{S}}}_{{\text{B}}}$ [44]. BayesB was implemented using the R package BGLR version 1.09 [44]. The analyses used a Gibbs chain of 200,000 iterations, with the first 50,000 iterations discarded as burn-in and a sampling interval of 10 cycles.

***Elastic net (ENET).*** The elastic net is a penalized regression that combines two regularization terms: ${{\text{l}}}_{1}=\sum |{\upbeta }_{{\text{j}}}|$ (least absolute shrinkage and selection operator – LASSO) and ${{\text{l}}}_{2}= \sum {\upbeta }_{{\text{j}}}^{2}$ (RR) (ridge regression – RR) [46]. The terms ${l}_{1}$ and ${l}_{2}$ in ENET are controlled by an alpha parameter (α), providing balance between selection (LASSO) and shrinkage (RR) of the predictor variables (SNPs). ENET is considered a robust approach when predictor variables have a strong collinearity. The regression model remains the same as previously described, and optimum weight values for $\lambda$ and $\alpha$ in the ENET are entered into the loss function as follows:

$${\text{L}}\left(\uplambda ,\mathrm{ \alpha },\upbeta \right)={\text{min}}\left[\frac{1}{2{\text{N}}} \sum\nolimits_{{\text{i}}=1}^{{\text{N}}}\{{{\text{y}}}_{{\text{i}}}- {(\upbeta }_{0}+\sum\nolimits_{{\text{w}}=1}^{{\text{p}}}{{\text{x}}}_{{\text{iw}}}{\upbeta }_{{\text{w}}}){\}}^{2}+\uplambda (\left(1-\mathrm{\alpha }\right){\upbeta }_{{\text{w}}}^{2}+\mathrm{\alpha }\sum |{\upbeta }_{{\text{w}}}|)\right],$$

where ${\text{N}}$ is the number of animals, $\mathrm{\alpha }$ has a value between 0 (RR penalty) and 1 (LASSO penalty), and $\uplambda$ is an overall regularization parameter that controls the variable shrinkage. A random grid search was performed to find optimal values of $\alpha$ and $\uplambda$ ranging from 0.0 to 1.0 with an interval of 0.1 for each parameter. We implemented the ENET model using the h2o R package (https://github.com/h2oai/h2o-3). The search for $\mathrm{\alpha }$ and $\uplambda$ was performed using the *h2o.grid* function with a cross-validation that splits the training subset into five folds for training and testing. Finally, the trained model with the highest accuracy and lowest mean square error (MSE) was applied to a disjoint validation set.

### Nonparametric methods

#### Gradient boosting machine (GBM)

GBM is an ensemble learning technique that combines gradient-based optimization and boosting techniques with regression tree models. A loss function is minimized during the training process, applying an iterative process of ensemble weak tree learners to obtain a robust predictive model [47]. GBM performs automatic variable selection, by prioritizing the variables that contribute more to trait variability and discarding those that contain irrelevant or redundant information. GBM starts by fitting a weak model based on the distribution of the response variable; subsequently, the algorithm fits models based on residual values of the previous model. Each new model aims to reduce the prediction error from the previous model; then, the algorithm stops when no further improvements in the loss function are achieved.

The GBM model can be described as ${{\text{y}}}_{{\text{i}}}^{*}=\upmu +\sum_{{\text{w}}=1}^{{\text{p}}}{{\varphi h}}_{{\text{m}}}({{\text{y}}}_{{\text{i}}}^{*};\mathbf{x})+{{\text{e}}}_{{\text{i}}}$, where ${{\text{y}}}_{{\text{i}}}^{*}$ is the adjusted phenotype; $\upmu$ is an overall average; ${{\text{h}}}_{{\text{m}}}({{\text{y}}}_{{\text{i}}}^{*};\mathbf{x})$ represents the ${\text{m}}$-th model built to predict the target information; ${\varphi }$ is the weight parameter applied to the ${\text{m}}$-th model; $\mathbf{x}$ is the genotype vector for the ${\text{i}}$-*th* animal at locus ${\text{w}}$ (coded as 0, 1, and 2), and p = 61,222; and ${{\text{e}}}_{{\text{i}}}$ is the residual effect. We explored a random search for hyperparameters for GBM as follows: the number of trees (Ntree) was 100, 200, 300, 500, 750, 1000, 1250, 1500, 2000, 2500 and 3000; learning rate (ln_rate) ranged from 0.01 to 1 with intervals of 0.05; maximum tree depth (max_depth) ranged from 0 to 50 with intervals of 5; and the minimum number of observations per leaf (node_size) ranged from 5 to 50 with interval*s* of 15.

### Stacking ensemble

The stacking ensemble is a meta-learning algorithm that is used to make the best combination of predictions from multiple previously trained ML models to further enhance the accuracy of prediction [48, 49]. Stacking ensemble prediction is performed in two steps: (1) obtaining output predictions from base learners that were previously trained using a random search (GBM and ENET), and (2) producing a final prediction using a generalized linear model (GLM) and a linear combination of weights (a_1_,…, a_n_) as follows: ${{\text{f}}}_{{\text{stacking}}}=\sum_{{\text{i}}=1}^{{\text{n}}}{{\text{w}}}_{{\text{i}}}{{\text{f}}}_{{\text{i}}}({\text{x}})$, where ${{\text{f}}}_{{\text{i}}}({\text{x}})$ represents the phenotype predicted from each base learner, and ${{\text{w}}}_{{\text{i}}}$ is the weight vector learned in the meta-learner. The stacking ensemble was performed using the *h2o.stackedEnsemble* function of the *h2o* R package.

### Model performances

Prediction accuracy of the different methods was assessed by the Pearson’s and Spearman’s correlation (${\text{r}}={\text{cor}}({{\text{y}}}_{{\text{i}}}^{*},{\widehat{{\text{y}}}}_{{\text{i}}}^{*})$) between phenotypes adjusted for fixed effects (${{\text{y}}}_{{\text{i}}}^{*}$) and predicted adjusted phenotype (${\widehat{{\text{y}}}}_{{\text{i}}}^{*}$) of animal ${\text{i}}$. The predictive root mean squared error (RMSE) was ${\text{RMSE}}=\sqrt{\sum_{{\text{i}}=1}^{{\text{n}}}({\widehat{{\text{y}}}}_{{\text{i}}}^{*}-{{\text{y}}}_{{\text{i}}}^{*}{)}^{2}/{\text{n}}}$, where ${\text{n}}$ is the number of animals in the validation set. The slope of the linear regression of ${{\text{y}}}^{*}$ on ${\widehat{{\text{y}}}}_{{\text{i}}}^{*}$ was also used to assess prediction bias. The relative difference (RD) in prediction accuracy was measured as ${\text{RD}}=\frac{({{\text{r}}}_{{\text{m}}1}-{{\text{r}}}_{{\text{m}}2})}{{{\text{r}}}_{{\text{m}}2}}\times 100$, where ${{\text{r}}}_{{\text{m}}1}$ is the predictive ability using the other models and ${{\text{r}}}_{{\text{m}}2}$ is the predictive ability using the GBLUP approach.

### Feature reduction prediction

In order to evaluate the effectiveness of ML in reducing dimensionality and improving prediction accuracy, we preselected 1500 SNPs for GBM using a two-step strategy. In the first step, we selected 1.5k SNPs that were labeled as most influential in the GBM analysis, using only the training set. In the second step, these selected SNPs were used in GBLUP, GBM, and Stack to train the model and make predictions in the validation set. This approach was applied separately, for each cross-validation fold, to ensure that marker selection was not based on information from the validation set. It should be noted that we did not include preselected SNPs to fit the BayesB model because it is a variable selection method. The feature importance score for GBM was determined by calculating the relative influence of improvements in predictive ability during the tree regression building process expressed in a percentage scale [47], and SNPs were ranked by their importance score.

## Results

### Variance component estimates for blood metabolites

The genomic heritabilities (h^2^) estimated using GBLUP ranged from 0.06 to 0.36 for energy-related metabolites, from 0.11 to 0.35 for liver function and hepatic damage indicators, from 0.09 to 0.37 for oxidative stress metabolites, from 0.06 to 0.41 for inflammation and innate immunity indicators, and from 0.12 to 0.27 for blood minerals (see Additional file 2: Tables S2 and S3). The lowest heritability estimates were for hapto (0.06 ± 0.021), NEFA (0.06 ± 0.009), FRAP (0.09 ± 0.015), BILt (0.11 ± 0.051), Na (0.12 ± 0.0717), Ca (0.14 ± 0.046), K (0.16 ± 0.0424), globulins (0.17 ± 0.053), BHB (0.17 ± 0.036), myeloperoxidase (0.18 ± 0.060) and Mg (0.19 ± 0.0625). Moderate heritability estimates were observed across a spectrum of minerals (P, Cl, and Zn), ranging from 0.21 to 0.27, and energy-related metabolites (glucose, cholesterol, urea, and creatinine), ranging from 0.28 to 0.36. Similarly, the heritability estimates ranged from 0.23 to 0.35 for function and hepatic damage markers (AST, GGT, Albumin, ALP, and PON) they ranged, from 0.21 to 0.37 for oxidative stress indicators (ROMt, AOPP, and SHp), and was equal to 0.21 ± 0.06 for PROTt. The highest heritability estimate, 0.41 ± 0.062, was found for CuCp.

Batch variances (${{\text{h}}}_{{\text{batch}}}^{2}$) ranged from 0.05 to 0.49, with values higher than 30% of the total variation observed for FRAP, AOPP, BILt, K, hapto, creatinine, urea, ALP, myeloperoxidase, Zn, PON, and Na. The contribution of the epistatic variance to the phenotypic variance of blood metabolites ranged from 0.06 to 0.42 and was found to be greater than the contribution of dominance effects, which ranged from 0.06 to 0.17, except for AST, albumin, AOPP, magnesium, and potassium [see Additional file 1: Fig. S5 and Additional file 2: Table S4]. The largest contribution from epistasis was observed for hapto, FRAP, ROMt, PON, cholesterol, Zn, Na, PROTt, and globulins.

### Model performance

In our study, we compared the predictive ability based on model fit parameters for tenfold and batch-out CV scenarios (see Additional file 2: Tables S5 to S14). Compared to the tenfold CV, the batch-out CV reduced the dependencies among training and validation sets, which decreased the predictive ability, on average, by 12% for the energy-related metabolites, 20% for liver function and hepatic damage, 15% for oxidative stress, 10% for inflammation and innate immunity and 14% for minerals. After comparing different approaches, a similar pattern was observed between the tenfold CV and batch-out CV scenarios, for which the use of a stacking ensemble showed better predictive ability. Thus, the tenfold CV was used for model comparisons.

**Fig. 1:**
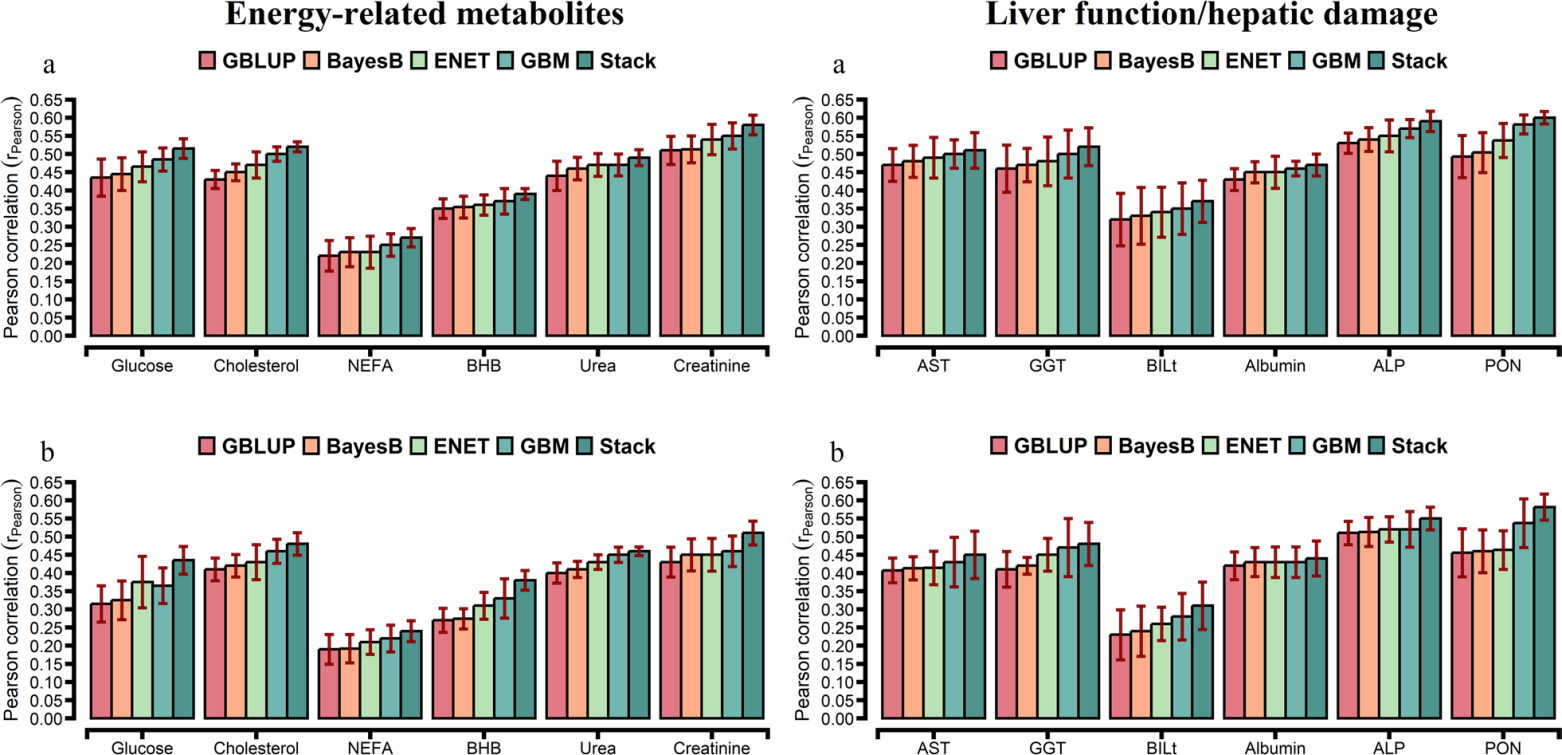
Prediction accuracy assessed by Pearson **a** and Spearman **b** correlations, including standard errors, obtained from genomic BLUP (GBLUP), BayesB, elastic net (ENET), gradient boosting machine (GBM), and stacking ensemble (Stack) for energy-related metabolites and liver function/hepatic damage. For more details, see Additional file 2: Tables S5 and S6. *NEFA* non-esterified fatty acids, *BHB* β-hydroxybutyrate, *AST* aspartate aminotransferase, *GGT* γ-glutamyl transferase, *BILt* total bilirubin; *ALP* alkaline phosphatase and PON paraoxonase

**Fig. 2:**
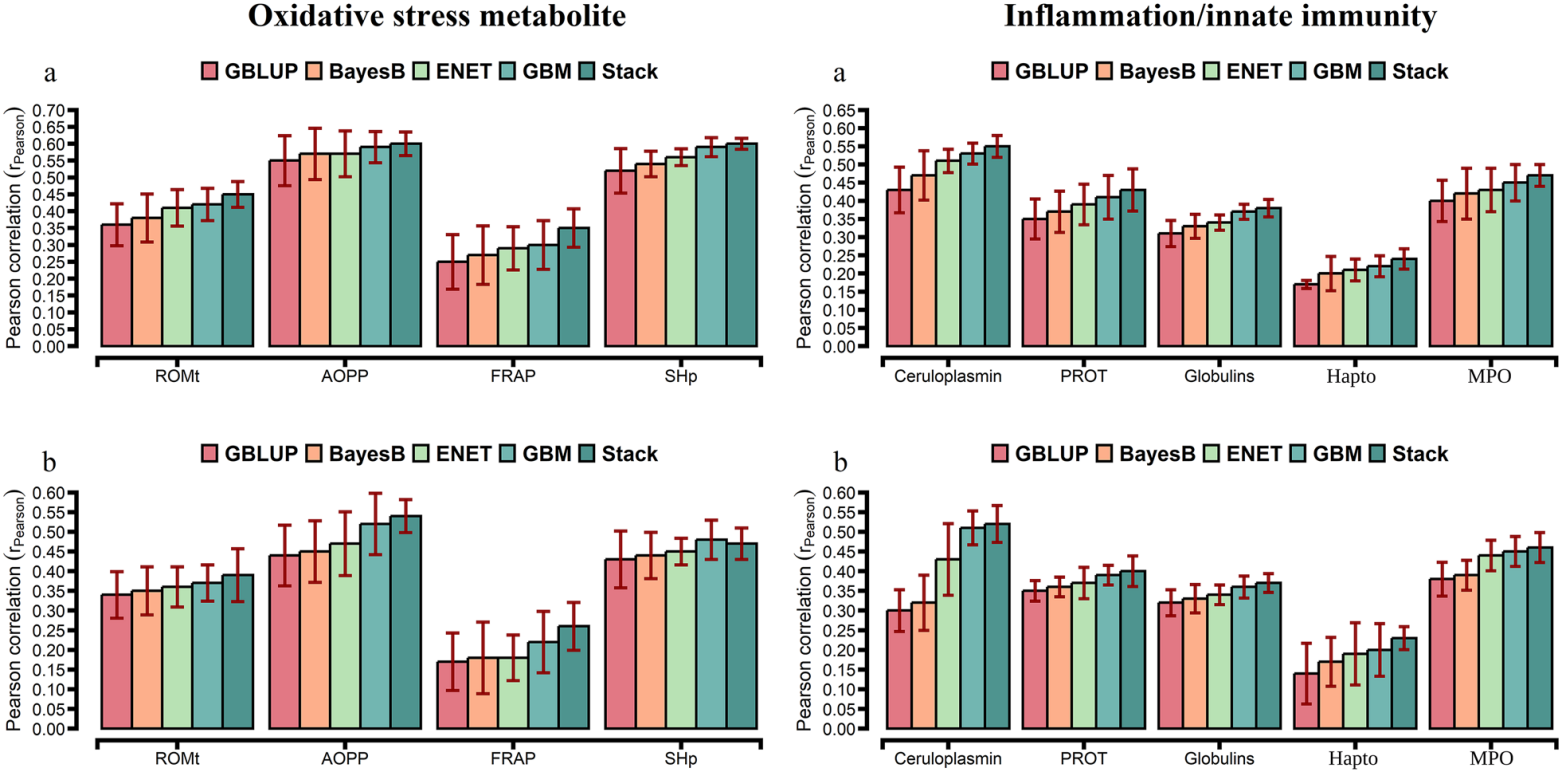
Prediction accuracy assessed by Pearson **a** and Spearman **b** correlations, including standard errors, obtained from genomic BLUP (GBLUP), BayesB, elastic net (ENET), gradient boosting machine (GBM), and stacking ensemble (Stack) for oxidative stress metabolites and inflammation/innate immunity. For more details, see Additional file 2: Tables S7 and S8. *ROMt* total reactive oxygen metabolites, *AOPP* advanced oxidation protein products, *FRAP* ferric reducing antioxidant power, *SHp* thiolic groups, and *PROT* total proteins

**Fig. 3:**
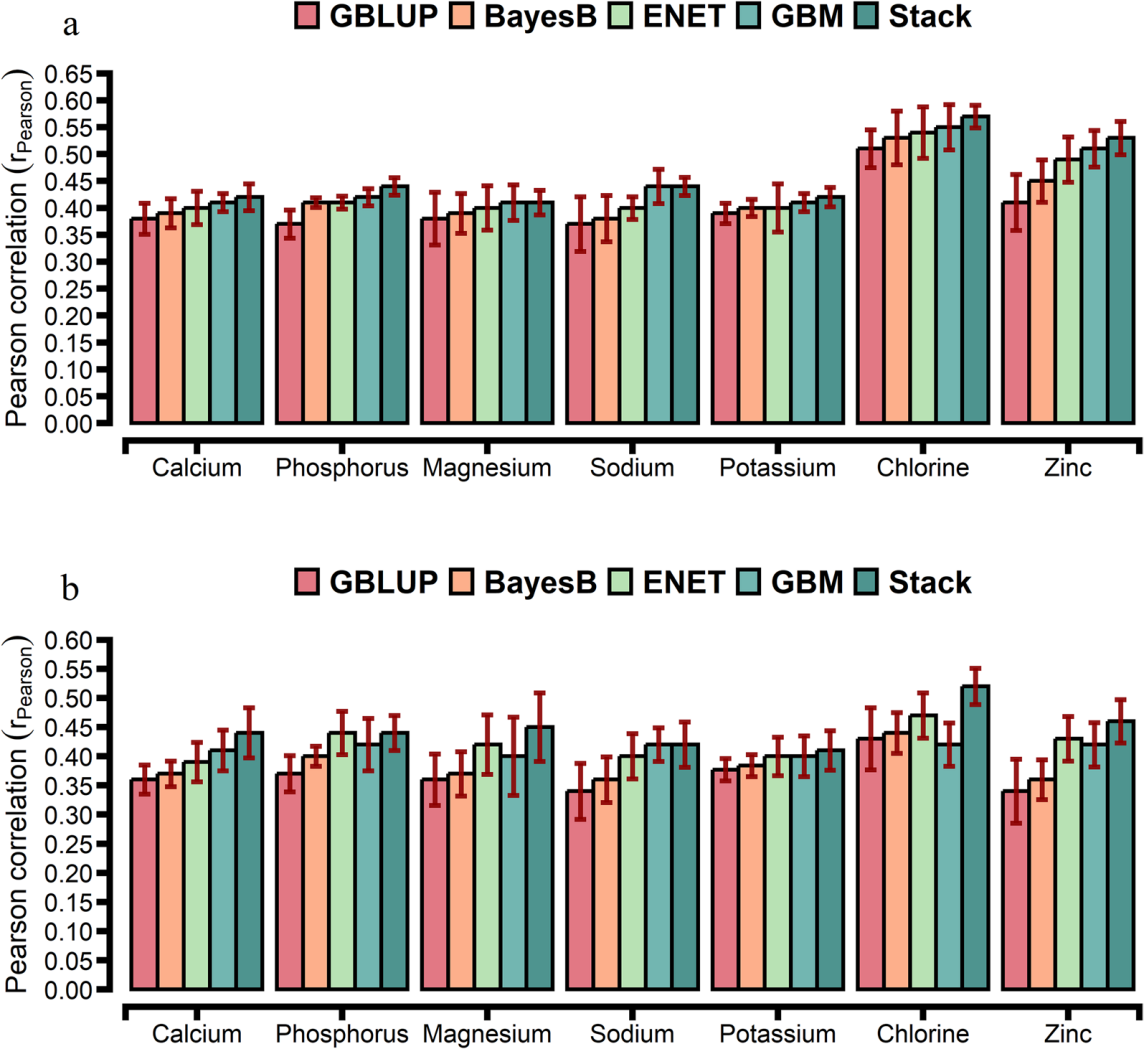
Prediction accuracy assessed by Pearson (**a**) and Spearman (**b**) correlations, including standard errors, obtained from genomic BLUP (GBLUP), BayesB, elastic net (ENET), gradient boosting machine (GBM), and stacking ensemble (Stack) for blood mineral. For more details, see Additional file 2: Table S9

**Fig. 4:**
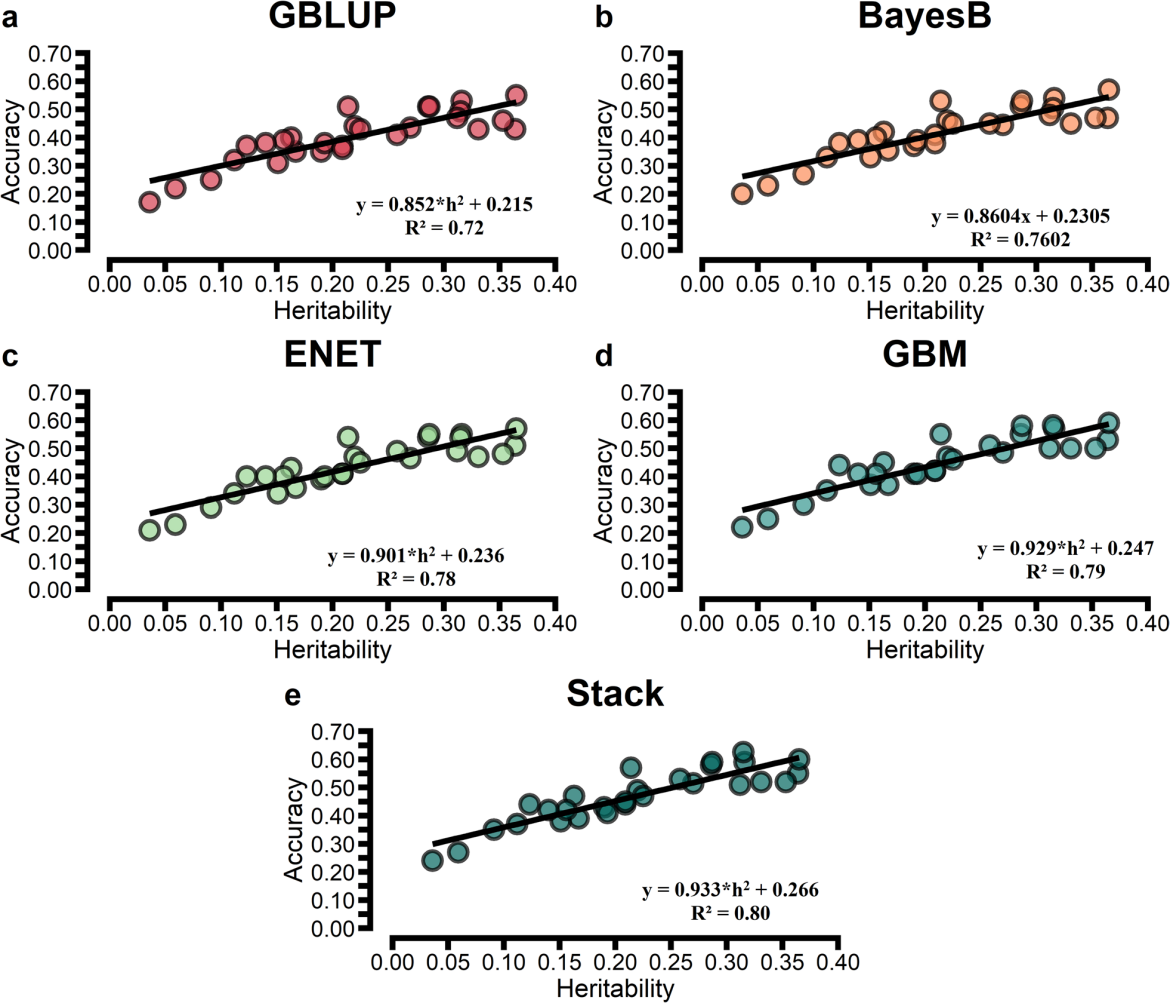
Relationship between prediction accuracy obtained using tenfold cross-validation and heritability estimates for blood metabolites: *GBLUP* genomic best linear unbiased prediction (**a**); BayesB (**b**); *ENET* elastic net (**c**), *GBM* gradient boosting machine (**d**) and *Stack* Stacking ensemble combining predictions from EN and GBM (**e**)

For the comparison of prediction accuracies, we used GBLUP as the benchmark against other statistical approaches. The prediction accuracy from the tenfold CV is shown in Figs. 1, 2, and 3. The prediction accuracy was positively correlated to the heritability of the trait for all approaches, with a higher association for ENT (R^2^ = 0.78), GBM (R^2^ = 0.79), and Stack (R^2^ = 0.80; Fig. 4). Prediction accuracies ranged from low (hapto, NEFA, and FRAP) to high (Cl, SHp, ALP, and AOPP). On average, predictions for liver function/hepatic damage traits (r = 0.48, ranging from 0.32 to 0.63) and for oxidative stress metabolites (r = 0.46, ranging from 0.25 to 0.60) were more accurate than predictions for energy-related metabolites (r = 0.43, ranging from 0.22 to 0.58), minerals (r = 0.43, ranging from 0.37 to 0.57), and inflammation/innate immunity indicators (r = 0.38, ranging from 0.17 to 0.55); see Figs. 1a, 2a and 3a.

GBLUP had a lower prediction accuracy than BayesB, ENET, GBM, and Stack (Figs. 1a, 2a and 3a). The Stack approach performed better than GBLUP, with accuracies ranging from 0.27 to 0.58 for energy-related metabolites, from 0.24 to 0.55 for inflammation/innate immunity, from 0.37 to 0.63 for liver function/hepatic damage, 0.35 to 0.60 for oxidative stress metabolites and 0.41 to 0.57 for minerals (Figs. 1a, 2a and 3a). On the one hand, relative differences (RD) between GBLUP and BayesB were very small for creatinine (0.59%), BHB (1.14%), ALP (1.89%), AST (2.13%), GGT (2.17%), PON (2.24%) and glucose (2.30%; see Additional file 1: Fig. S6a). On the other hand, greater RD were observed for all methods, i.e. for hapto, ranging from 17.65% for BayesB to 41.18% for Stack; Zn, ranging from 9.76% for BayesB to 29.27% for Stack; CuCp, ranging from 9.30% for BayesB to 27.91% for Stack; FRAP, ranging from 8% for BayesB to 40% for Stack; and PROTt, ranging from 5.7% for BayesB to 22.86% for Stack [see Additional file 1: Figs. S6a, S7a, and S8a]. Prediction accuracy of BayesB was similar to that of GBLUP (for cholesterol and NEFA) and of ENET (for albumin, AOPP, NEFA, P, and K), and ENT and GBM showed similar prediction accuracy for urea (Figs. 1a, 2a and 3a).

**Fig. 5:**
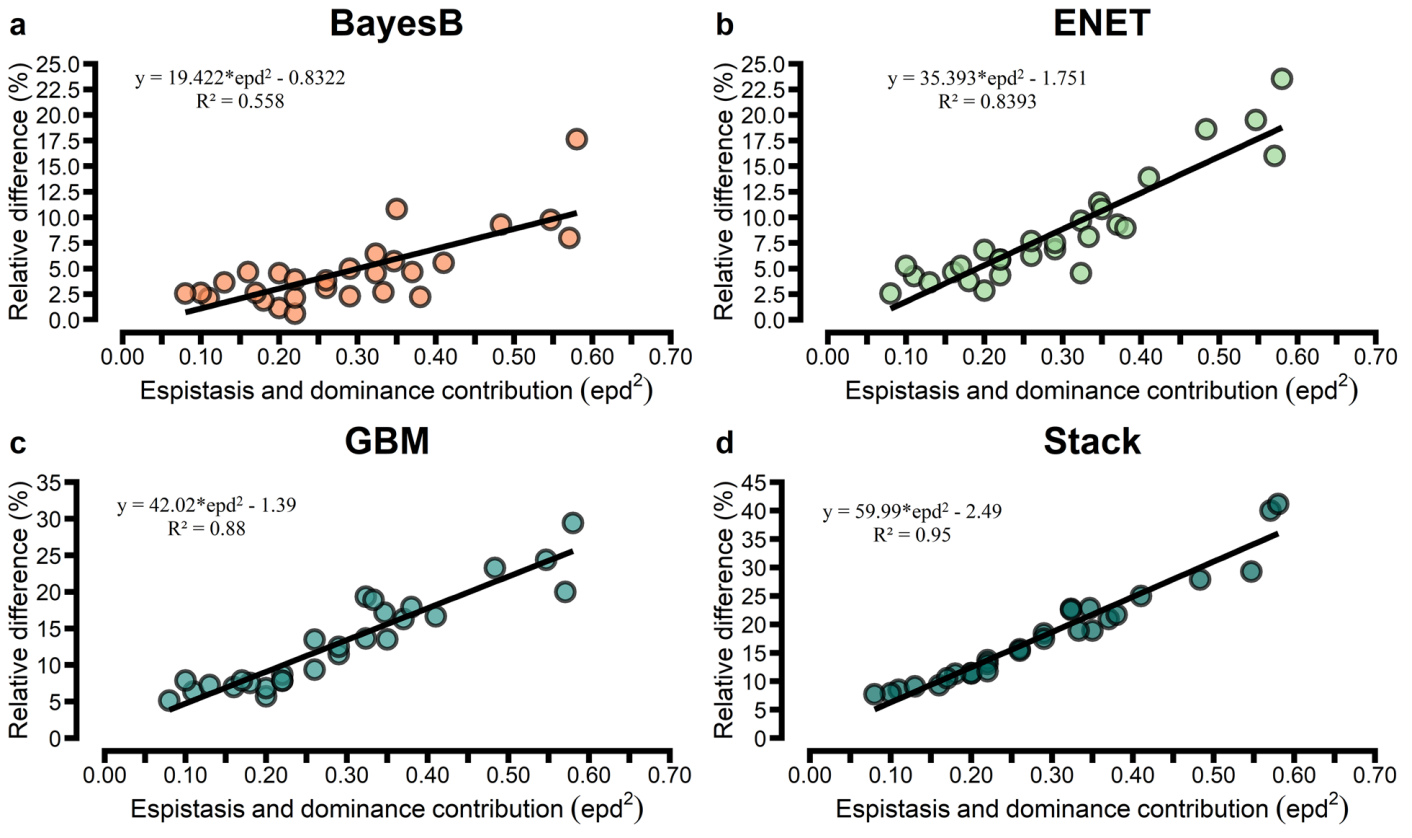
Relationship between relative gain in prediction accuracy obtained using tenfold cross-validation and non-additive effect for blood metabolites assessed by Pearson’s correlation for the approaches BayesB, elastic net (EN), gradient boosting machine (GBM) and stacking ensemble (Stack) against genomic best linear unbiased prediction (GBLUP)

Comparing alternative models to the GBLUP baseline model based on RD, ML approaches such as GBM and Stack increased the prediction accuracy as the non-additive effect on the trait increased (Fig. 5). A stronger association between RD and non-additive effect was observed for Stack (R^2^ = 0.95), GBM (R^2^ = 0.88) and ENET (R^2^ = 0.86), even for traits that are polygenic, and a moderate association was observed for BayesB (R^2^ = 0.55). However, when the blood phenotypes exhibited greater non-additive gene action (see Additional file 1: Fig. S5), the parametric models BayesB and ENET achieved gains in prediction accuracy over GBLUP.

The Spearman’s correlation measures differences in rank order between adjusted and predicted values, with higher values for Stack (0.23–0.58) than for GBM (0.23–0.54), ENET (0.18–0.52), BayesB (0.17–0.51) and GBLUP (0.14–0.51). Concerning RMSE, Stack had the best performance for all traits, with a larger reduction in RMSE ranging from 11 to 35% for traits related to liver function/hepatic damage, oxidative stress, and minerals (see Additional file 2: Table S5, S6, S7, S8 and S9). ENET had a higher RMSE than GBLUP for glucose, cholesterol, and urea, although it had a better predictive ability (see Additional file 2: Table S5). The slope coefficients differed slightly from 1, so the predictions were empirically unbiased for the GP methods evaluated (see Additional file 2: Tables S5, S6, S7, S8 and S9). However, using a hierarchical cluster, we observed that Stack, ENET, and GBLUP were similar to each other but different from GBM and BayesB (see Additional file 1: Fig. S9).

### Impact of GBM feature selection on prediction accuracy

**Fig. 6:**
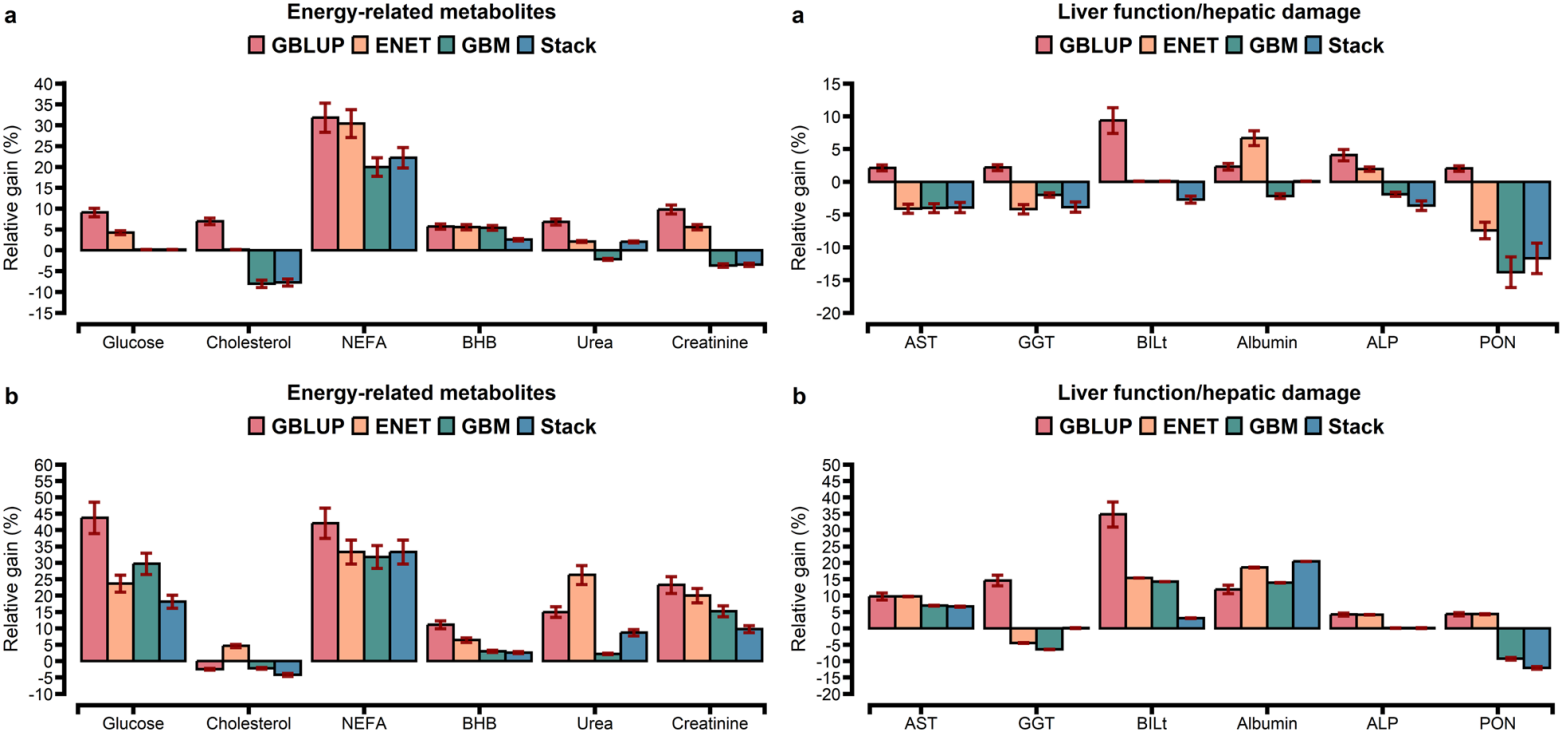
Relative gain in prediction accuracy assessed by Pearson **a** and Spearman **b** correlations considering the top 1500 SNPs ranked by a GBM model against fitting all 61k SNPs, including standard errors, for the energy-related metabolites and liver function/hepatic damage traits using GBLUP, ENET, GBM, and stacking ensemble (Stack). *NEFA* non-esterified fatty acids, *BHB* β-hydroxybutyrate, *AST* aspartate aminotransferase, *GGT* γ-glutamyl transferase, *BILt* total bilirubin, *ALP* alkaline phosphatase, *PON* paraoxonase

**Fig. 7:**
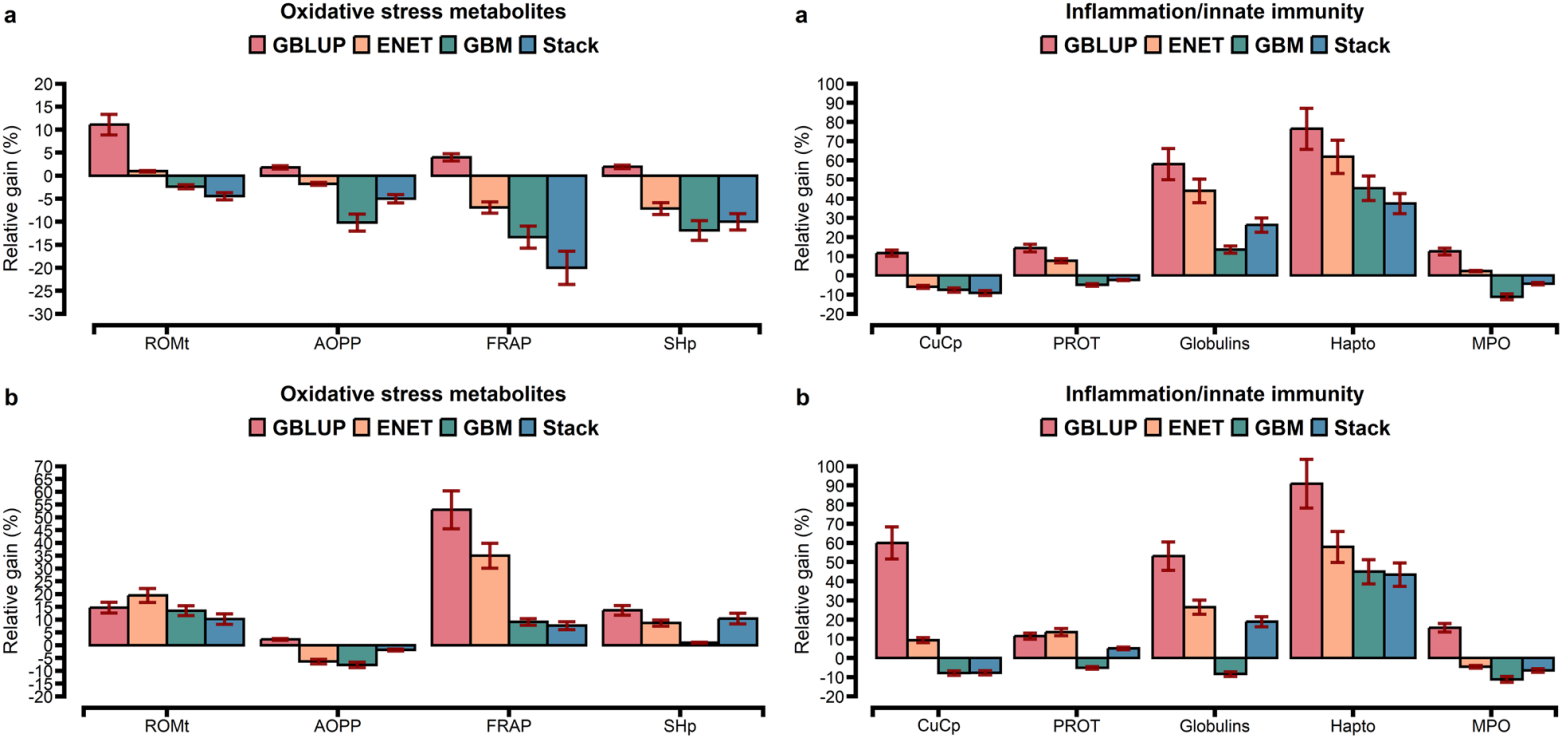
Relative gain in prediction accuracy assessed by Pearson **a** and Spearman **b** correlations considering the top 1500 SNPs ranked by a GBM model against fitting all 61k SNPs, including standard errors, for oxidative stress metabolites and inflammation/innate immunity traits using GBLUP, ENET, GBM, and stacking ensemble (Stack). *ROMt* total reactive oxygen metabolites, *AOPP* advanced oxidation protein products, *FRAP* ferric reducing antioxidant power, *SHp* thiolic groups, *CuCp* ceruloplasmin, *PROTt* total proteins, *Hapto* haptoglobin, *MPO* myeloperoxidase

Figures 5, 6 and 7 show the relative gain in prediction accuracy obtained by GBLUP, ENET, GBM, and Stack, considering all SNPs (~ 61 K) against the model fitting only the 1500 SNPs selected as most relevant from the GBM approach. The relative gain had distinct patterns depending on blood metabolite and model. Using preselected SNPs in the GBLUP method increased accuracy when it was assessed using Pearson’s correlation for all metabolites (Fig. 5a) while when it was assessed using Spearman’s correlation, only cholesterol showed a reduction of 2% (Fig. 5b).

**Fig. 8:**
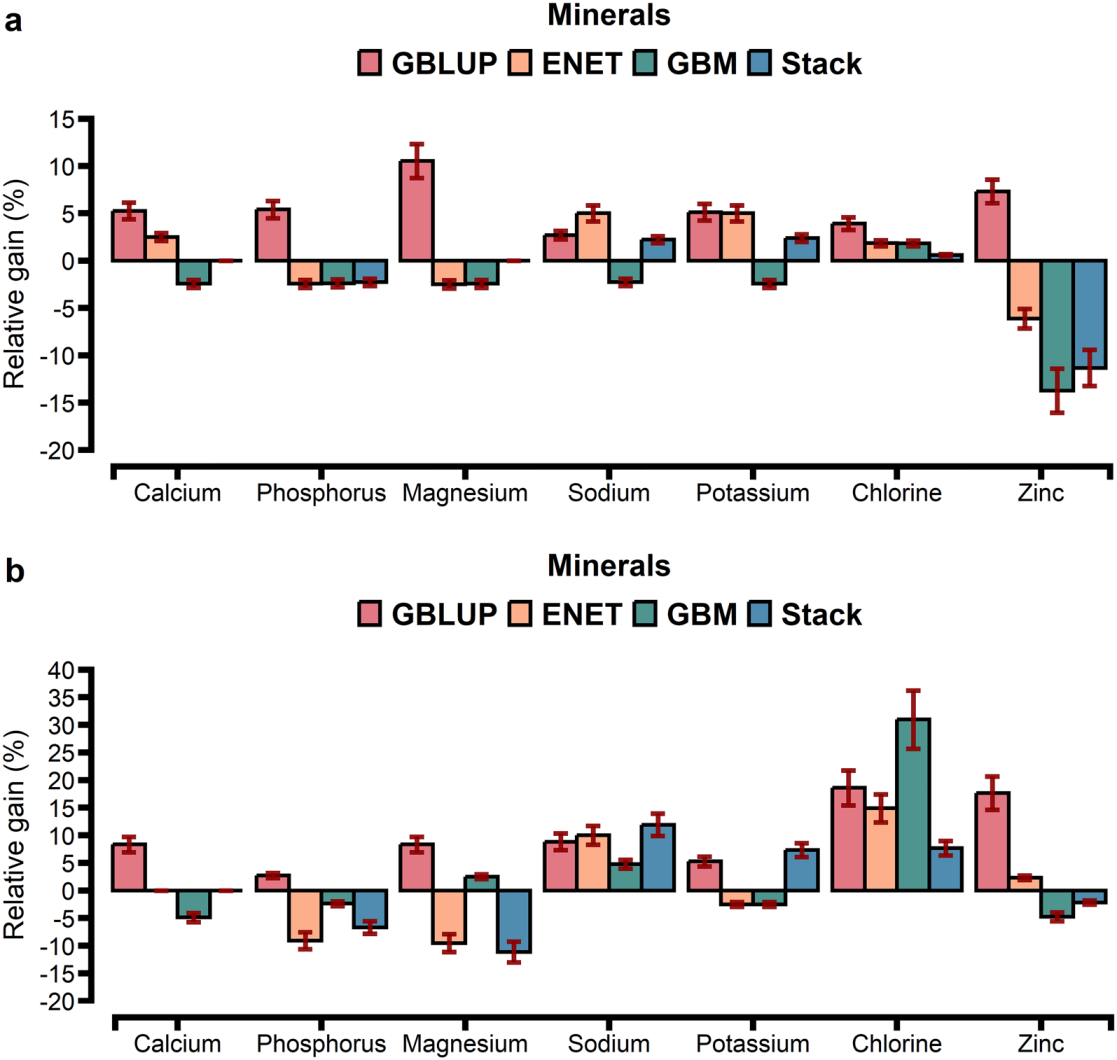
Relative gain in prediction accuracy assessed by Pearson (**a**) and Spearman (**b**) correlations considering the top 1500 SNPs ranked by a GBM model against fitting all 61k SNPs, including standard errors, for blood minerals using GBLUP, ENET, GBM, and stacking ensemble (Stack)

Using preselected SNPs, the GBLUP approach had a relative gain, ranging from 2 to 76%, while ENET, GBM, and Stack showed a reduction in prediction accuracy for some blood metabolites (Figs. 5a, 6a, and 7a). GBLUP showed significant gains in prediction accuracy for hapto (76%), globulins (58%), and NEFA (32%), but such gains were also observed for the ENET (62%, 44%, and 30%), GBM (45%, 14%, and 20%), and Stack (38%, 26%, and 22%) models. When fitting ENET with preselected SNPs as predictors, ENET showed a similar trend to that of GBLUP i.e. an increase in prediction accuracy, except for 10 blood metabolites (PON, SHp, FRAP, Zn, CuCp, GGT, AST, Mg, P, and AOPP) for which prediction accuracy decreased. For the GBM approach, among the 28 blood metabolites evaluated, 21 exhibited a reduction in prediction accuracy (Figs. 5a, 6a, and 7a), which ranged from 14% (PON and Zn) to 2% (Mg, Ca, K, ROMt, P, Na, albumin, urea, GGT, and ALP), and the only exceptions were BILt and glucose, for which inclusion of relevant preselected SNPs resulted in the same prediction accuracy as when all SNPs were used (Fig. 8).

Using Spearman’s correlation, GBLUP showed higher gains in prediction accuracy than ENET, GBM, and Stack when considering the subset of SNPs, except for cholesterol, which showed a reduction of 2% (Figs. 5b, 6b and 7b). Overall, although GBLUP achieved a greater gain in prediction accuracy when using the subset of SNPs compared to all available SNPs, the Stack model remained the model with the highest prediction accuracy (see Additional file 2: Tables S15, S16, S17, S18 and S19).

## Discussion

The data used in this work were from five herds, which differed in the health measures implemented, resulting in differences in the prevalence of metabolic disorders and clinical/subclinical mastitis. Although the trial did not include cows with clinical diseases, it did encompass cows with differing degrees of subclinical conditions, as determined by the proportion of individuals with deviating values for blood traits such as hapto and urea (see Additional file 2: Table S1). This phenomenon, frequently observed on dairy farms, was recently examined by Giannuzzi et al. [50]. Their study demonstrated a strong connection between individual metabolic behavior and production performance, which indicated that this association is not confined to specific clinical diseases or the transition phase but extends throughout the entire lactation period.

### Variance components for blood metabolites

Estimation of genetic parameters is fundamental for using the existing genetic variation to select high-performing dairy cattle with a lower susceptibility to metabolic disorders. This study estimated heritability for energy-related serum metabolites, liver function/hepatic damage, oxidative stress, inflammation/innate immunity, and minerals. Heritability estimates for some of these metabolites have previously been estimated in milk, but few studies have reported values for the serum matrix. On the one hand, compared to the estimates of heritability reported by Luke et al. [10], those obtained in our study for serum metabolites were higher for BHB (0.09 ± 0.04), Ca (0.07 ± 0.04), and urea (0.18 ± 0.05), lower for albumin (0.27 ± 0.06) and globulin (0.46 ± 0.06) and similar for Mg (0.19 ± 0.06) and hapto with values close to zero. On the other hand, Benedet et al. [51] observed higher heritability estimates for blood infrared predicted metabolites such as BHBA, i.e. 0.21 ± 0.02 and NEFA, i.e. 0.14 ± 0.02, while Mota et al. [20] observed a slight difference in heritability estimates for some serum metabolites.

The heritability estimates for serum minerals in this study were lower than those of Tsiamadis et al. [52]. Their study reported moderate to high heritabilities (0.20–0.43) for Ca, P, and Mg, while those for K were low to moderate (0.12–0.23). It is worth mentioning that heritability estimates can differ depending on the population, management methods, and statistical model used. Furthermore, various environmental factors, including diet and interaction with other genetic and non-genetic factors, also play a role in the variance of blood metabolite levels. In general, availability of the heritability estimates for blood metabolites in dairy cattle can offer useful insights into the genetic basis of metabolic health and production traits. This knowledge can drive breeding strategies to enhance these traits in dairy cattle populations.

Although additive effects play a major role in blood metabolites levels, non-additive effects should not be neglected. Our findings reveal significant contributions of non-additive variance captured by SNPs in determining blood metabolites. The dominance variance contributed a further 6 to 17% to the total phenotypic variance (see Additional file 1: Fig. S5). Moreover, our study found that from 6 to 42% of the phenotypic variance could be attributed to epistatic variation depending on the serum metabolite evaluated. To our knowledge, this is the first time that non-additive genetic variance components have been reported for serum metabolites in Holstein. Differences in estimated additive genetic variance and heritability were found between models with and without non-additive effects. This may occur because the partition of non-additive effects, especially epistasis, can be translated into additive variance when allelic frequencies are low [53]. In addition, the additive variance captured from the similarities between relatives may also include a fraction of the variance generated by interaction effects [53]. This could partially explain the observed difference in additive variance between models.

### Predictive performance of genomic prediction

Genomic prediction of complex traits has used mainly parametric methods. However, increased attention has recently been directed to nonlinear ML techniques. Few studies reported accuracies of GP for some of the metabolites evaluated in this study, such as BHBA, albumin, urea, globulin, Ca, and Mg [10, 11]. Overall, our results indicate low-to-medium predictive accuracies, ranging from 0.17 to 0.60 depending on the trait analyzed and statistical approach. These values are higher than those reported by Luke et al. [10], which ranged from 0.09 to 0.27, and similar to those reported by Cavani et al. [11] for Ca, which was 0.45. The performance of the evaluated methods seems to depend highly on the genetic architecture of the traits measured by the heritability, even for ML approaches (Fig. 4). This may be due to non-additive gene action in the ML (GBM and Stack) approaches, such as epistasis, which can be converted into additive genetic variance when allelic frequencies are low for complex traits under directional selection, or to similarity between relatives, which can also contain a fraction of the variance generated by interaction effects [53].

The differences observed in prediction accuracy between models stem from assumptions about the effect of SNPs on the target trait, which directly affects the model's ability to adapt to changes in phenotypic value (bias) and prediction variance (RMSE). This occurs when the assumptions of the prediction model are inappropriate for a given trait, and thus they impair its predictive ability. The assumption in GBLUP (i.e., the SNP contribution to trait variability is uniform across the whole genome) may not match the genetic architecture underlying the traits. Alternatively, BayesB, ENET, and GBM assign differential weights to SNPs, attempting to match their contributions to the variation of the target trait, whereas, Stack ensemble learning aims at improving prediction accuracy by combining heterogeneous base learners [49]. The Stack approach improved the prediction accuracy from 7.7 to 41.2% over the base learning methods (GBM and ENET). In the Stack model, the GBM approach builds a nonlinear model, whereas ENET uses a penalized linear model where some regression coefficient estimates are set to zero, thus performing variable selection [46]. Several authors have observed that combining heterogeneous base learning algorithms can give better generalization and more accurate phenotypes [9, 54] and genomic predictions [29].

We found that BayesB (differential shrinkage) performed better than GBLUP (homogeneous shrinkage). With a substantial superiority for blood metabolites, BayesB performed better than GBLUP, with an RD ranging from 1.89 to 17.65%, except for glucose and cholesterol for which the RD was only 0.59% and 1.14%, respectively. BayesB regression performs better for target traits with QTL with significant effects [55–57]. The results of genome-wide association studies have shown that several major chromosomal regions influence specific blood metabolites, leading to variability in their phenotypic expression. For instance, Nayeri et al. [58] found that most of the significant SNPs for milk BHB were located on *Bos taurus* (BTA) autosome 6, while Milanesi et al. [18] identified different BTA chromosomes with significant associations for CuCp (BTA1), GGT (BTA17), and PON (BTA4, 16, and 26). In addition, Cavani et al. [11] found that the genomic region located on BTA6 explained the largest percentage of additive genetic variance for blood Ca. Thus, considering GP approaches that weigh these regions differently can improve prediction accuracy.

The gain in prediction accuracy from ENET comes from the combination of two different types of penalties to the predictor variables, thus reducing the overfitting in the training population by controlling the degree of shrinkage and imposing more flexible penalties to predictors, and shrinking coefficients to zero or close to zero [59]. Piles et al. [30] found that feature selection approaches improved prediction accuracy. Furthermore, Azodi et al. [32] indicated that incorporating feature selection in ensemble methods increased accuracy compared to models that did not allow variable selection.

GP involves the estimation of GEBV or adjusted phenotypes based on genetic markers with the aim of extracting pattern and similarity relationships between markers and the target information. In general, the traditional approaches (i.e., GBLUP and Bayesian regression) used in GP might not adequately capture the complexity of the genetic architecture of traits (e.g., dominance and epistasis) [27, 30, 60, 61]. In this context, using the GBM approach, which does not assign prior knowledge about how genes affect traits, we observed a significant improvement in prediction accuracy over GBLUP. On average, prediction accuracy increased by 10.29% for energy-related metabolites, 9.48% for liver function and hepatic damage, 14.42% for oxidative stress metabolites, 20.33% for inflammation and innate immunity indicators, and 12.23% for minerals. These gains in predictive ability from GBM may be due to blood metabolites being affected by nonlinear relations (dominance and epistasis) between SNPs and phenotypes, which contribute to their variability (see Additional file 1: Fig. S5).

GBM is an attractive ensemble approach for handling a large number of predictors because it is a robust supervised learning algorithm that can learn complex nonlinear relationships. Compared with GBLUP, BayesB, and ENET, GBM has flexibility in capturing complex genetic architectures with additive and epistatic effects. Howard et al. [62] observed that when a trait is influenced by additive-by-additive epistasis, parametric approaches could not make accurate predictions even for traits with a high heritability, while nonparametric methods provided reasonably accurate predictions. Given that there are well-defined chromosomal regions that affect blood metabolites such as Ca [11], PON, GGT and CuCp [18] and BHBA [58, 63], statistical approaches that use differential shrinkage (ENET and BayesB) of SNP effects may have advantages over GBLUP.

### GBM feature selection on prediction accuracy

Feature selection and ML learners have the power to deal with heterogeneous and large datasets, providing prediction accuracies and detecting genomic regions that impact the relationships between genotype and phenotype [30, 31]. Efficient feature selection from GBM enables the preselection of SNPs with biological relevance to the target trait. Furthermore, GBM can provide high accuracy in genomic prediction when the number of SNPs is reduced [60]. However, the performance of GBM and Stack using preselected SNPs (1.5k) was lower than that obtained using all the SNPs (~ 61k) except for glucose, NEFA, BHB, urea, albumin, globulins, Hapt, Ca, P, Mg, Na, k, and Cl. However, for GBLUP, we observed an increment in prediction accuracy for all blood metabolites using the preselected SNPs, and this gain was more evident when Spearman’s correlation was used. Azodi et al. [32] observed that selecting SNPs using random forest improved prediction accuracies for artificial neural networks in the context of various plant species, but its prediction accuracy was lower than for the other models evaluated. Similarly, our findings show that while GBLUP produced gains in prediction accuracy, the Stack had the highest prediction accuracy.

Li et al. [60] used three regression tree-based ensemble learning methods (random forest, GBM, and extreme gradient boosting) and observed that, when SNPs were preselected from GBM, the accuracies of GBLUP for some traits were similar to those obtained when using all the SNPs. In addition, Piles et al. [30] and Azodi et al. [32] observed that combining feature selection approaches with parametric and nonparametric models increased prediction accuracy compared to models without variable selection. In a study on the prediction of complex phenotypes in outbred mice, Perez et al. [64] observed that selecting SNPs with GBM seemed advantageous to decrease the number of predictor variables and, in some cases, improved the accuracy of parametric models. Together with applying insights from our findings, preselecting informative SNPs from the GBM approach can be a dimension reduction strategy, even when GP is performed using a parametric model (GBLUP). Further research is required to extend this alternative from a univariate to a multivariate approach for practical implementation in genomic selection breeding programs. In this study, when compared to the predictive performance of the models evaluated, the Stack approach delivered competitive results for blood metabolites (Figs. 1, 2 and 3).

## Conclusions

The Stack approach exhibited better performance for genomic prediction of complex blood metabolites in Holstein cattle, specifically for traits that are affected by non-additive effects (dominance and epistasis), where it outperformed parametric models (GBLUP, BayesB, and ENET). In this context, more research should be carried out to increase the knowledge of the biology of these indicators and consequently on the selection direction (i.e., selection for increasing or decreasing the values). Preselecting SNPs from GBM seems beneficial for extracting a number of informative SNPs and improving the prediction accuracy of GBLUP compared with ENET, GBM, and Stack. However, validation of these results using a larger cohort of animals and/or different breeds and herds is needed.

### Supplementary Information

**Additional file 1:** **Figure S1.** Principal component analysis based on SNPs (**a**) and genomic relationship matrix (**b**) for Holstein cows. Principal component analysis of animals based on the first two principal components based on SNPs to evaluate the extent of the population structure (**a**) and genomic relationship matrix (**b**) in the Holstein cows. The colors represent the tenfold used in the cross-validation of genomic breeding values. **Figure S2.** Distribution of phenotypic values for energy-related metabolites: BHBA – β-hydroxybutyric acid; Cholest – cholesterol; CREA – creatinine; GLU – Glu – glucose; NEFA – non-esterified fatty acids and urea. **Figure S3.** Distribution of phenotypic values for liver function and hepatic damage, (**a**) and oxidative stress (**b**). Distribution of phenotypic values of blood metabolites related to liver function and hepatic damage (**a**): ALB – albumin; ALP – alkaline phosphatase; AST – aspartate aminotransferase; BILt – total bilirubin; GGT – γ -glutamyl transferase; and PON – paraoxonase and oxidative stress (**b**): AOPP – advanced oxidation protein products; FRAP – ferric reducing antioxidant power; RMT – total reactive oxygen metabolites; SHp – thiol groups. **Figure S4.** Distribution of phenotypic values for inflammation/innate immunity response (**a)** and mineral (**b**). Distribution of phenotypic values for blood metabolites related to inflammation/innate immunity (**a**): Hapto – haptoglobin; CuCp – ceruloplasmin; GLOB – globulins; MPO – myeloperoxidase; PROTt – total protein and mineral (**b**): CA – calcium; CL – chlorine; K – potassium; MG – magnesium; Na – sodium; P – phosphorus and Zn – zinc. **Figure S5.** Dominance (${{\text{d}}}^{2}$), additive-by-additive epistasis (${{\text{ep}}}_{{\text{aa}}}^{2})$, and dominance and epistasis (${{\text{epd}}}^{2}$) contribution for blood metabolites variability. Dominance (${{\text{d}}}^{2}$), additive-by-additive epistasis (${{\text{ep}}}_{{\text{aa}}}^{2})$, and dominance and epistasis (${{\text{epd}}}^{2}$) contribution for blood metabolites variability estimated as a proportion of total phenotypic variance considered as ${{\text{d}}}^{2}={\upsigma }_{{\text{d}}}^{2}/({\upsigma }_{{\text{a}}}^{2}+ {\upsigma }_{{\text{d}}}^{2}+{\upsigma }_{{{\text{ep}}}_{{\text{aa}}}}^{2}+{\upsigma }_{{\text{batch}}}^{2}+{\upsigma }_{{\text{e}}}^{2})$, ${{\text{ep}}}_{{\text{aa}}}^{2}={\upsigma }_{{{\text{ep}}}_{{\text{aa}}}}^{2}/({\upsigma }_{{\text{a}}}^{2}+ {\upsigma }_{{\text{d}}}^{2}+{\upsigma }_{{{\text{ep}}}_{{\text{aa}}}}^{2}+{\upsigma }_{{\text{batch}}}^{2}+{\upsigma }_{{\text{e}}}^{2})$ and ${{\text{epd}}}^{2}=({\upsigma }_{{\text{d}}}^{2}+{\upsigma }_{{{\text{ep}}}_{{\text{aa}}}}^{2})/({\upsigma }_{{\text{a}}}^{2}+ {\upsigma }_{{\text{d}}}^{2}+{\upsigma }_{{{\text{ep}}}_{{\text{aa}}}}^{2}+{\upsigma }_{{\text{batch}}}^{2}+{\upsigma }_{{\text{e}}}^{2})$ where ${\upsigma }_{{\text{a}}}^{2}$, ${\upsigma }_{{\text{d}}}^{2}$, ${\upsigma }_{{{\text{ep}}}_{{\text{aa}}}}^{2}$ and ${\upsigma }_{{\text{e}}}^{2}$ represents the additive genetic variance, dominance variance, additive-by-additive variance, and residual variance, respectively. **Figure S6**. Average of the relative difference (%) in predictive ability assessed by Pearson (**a**) and Spearman (**b**) correlations for statistical approaches against the genomic best linear unbiased prediction (GBLUP), for energy-related metabolites and liver function/hepatic damage blood metabolites in Holstein cows. Average of the relative difference (%) in predictive ability assessed by Pearson (**a**) and Spearman (**b**) correlations across 10-folds random cross-validation for the approaches BayesB, elastic net (EN), gradient boosting machine (GBM) and stacking ensemble (Stack) against the genomic best linear unbiased prediction (GBLUP), for energy-related metabolites and liver function/hepatic damage blood metabolites in Holstein cows. Data are shown as mean ± SD (black error bar line). NEFA—non-esterified fatty acids; BHBA—β-hydroxybutyric acid; AST—aspartate aminotransferase; GGT—γ-glutamyl transferase; BILt—total bilirubin; ALP—alkaline phosphatase and PON – paraoxonase. **Figure S7.** Average of the relative difference (%) in predictive ability assessed by Pearson (**a**) and Spearman (**b**) correlations for statistical approaches against the genomic best linear unbiased prediction (GBLUP), for oxidative stress and inflammation/innate immunity response blood metabolites in Holstein cows Average of the relative difference (%) in predictive ability assessed by Pearson (**a**) and Spearman (**b**) correlations across tenfold random cross-validation for the approaches BayesB, elastic net (EN), gradient boosting machine (GBM) and stacking ensemble (Stack) against genomic best linear unbiased prediction (GBLUP), for blood metabolites in Holstein cows. Data are shown as mean ± SD (black error bar line). ROMt—total reactive oxygen metabolites; AOPP—advanced oxidation protein products; FRAP—ferric reducing antioxidant power; SHp—thiol groups; PROTt—total proteins. **Figure S8.** Average of the relative difference (%) in predictive ability assessed by Pearson (**a**) and Spearman (**b**) correlations for statistical approaches against the genomic best linear unbiased prediction (GBLUP), blood minerals in Holstein cows. Average of the relative difference (%) in predictive ability assessed by Pearson (**A**) and Spearman (**B**) correlations across tenfold random cross-validation for the approaches BayesB, elastic net (EN), gradient boosting machine (GBM) and stacking ensemble (Stack) against genomic best linear unbiased prediction (GBLUP), for blood minerals in Holstein cows. **Figure S9.** Ward’s hierarchical clustering of models based on slope values for each model across all trait combinations.

**Additional file 2:** **Table S1.** Descriptive statistics for blood metabolites related to energy, liver function/hepatic damage, oxidative stress, inflammation/innate immunity, and minerals (n = 1353). **Table S2.** Estimates of variance components, heritability (${{\text{h}}}^{2}$), and batch incidence (${{\text{h}}}_{{\text{batch}}}^{2}$) for blood metabolites related to energy-related metabolites, liver function/hepatic damage, and oxidative stress metabolites. **Table S3.** Estimates of variance components, heritability (${{\text{h}}}^{2}$), and batch incidence (${{\text{h}}}_{{\text{batch}}}^{2}$) for blood metabolites related to inflammation/innate immunity and minerals. **Table S4.** Estimates of variance components considering non-additive effects, heritability (${{\text{h}}}^{2}$), batch incidence (${{\text{h}}}_{{\text{batch}}}^{2}$), dominance (${{\text{d}}}^{2}$), and additive-by-additive epistasis (${{\text{ep}}}_{{\text{aa}}}^{2})$, for blood metabolites. **Table S5.** Prediction metrics with standard errors, obtained from genomic BLUP (GBLUP), BayesB, elastic net (ENET), gradient boosting machine (GBM), and stacking ensemble (Stack) for energy-related metabolites for tenfold cross-validation. **Table S6.** Prediction metrics with standard errors, obtained from genomic BLUP (GBLUP), BayesB, elastic net (ENET), gradient boosting machine (GBM), and stacking ensemble (Stack) for liver function/hepatic damage for tenfold cross-validation. **Table S7.** Prediction metrics with standard errors, obtained from genomic BLUP (GBLUP), BayesB, elastic net (ENET), gradient boosting machine (GBM), and stacking ensemble (Stack) for oxidative stress metabolites for tenfold cross-validation. **Table S8.** Prediction metrics with standard errors, obtained from genomic BLUP (GBLUP), BayesB, elastic net (ENET), gradient boosting machine (GBM), and stacking ensemble (Stack) for inflammation/innate immunity for tenfold cross-validation. **Table S9.** Prediction metrics with standard errors, obtained from genomic BLUP (GBLUP), BayesB, elastic net (ENET), gradient boosting machine (GBM), and stacking ensemble (Stack) for blood minerals for tenfold cross-validation. **Table S10.** Prediction metrics with standard errors, obtained from genomic BLUP (GBLUP), BayesB, elastic net (ENET), gradient boosting machine (GBM), and stacking ensemble (Stack) for energy-related metabolites for batch-out cross-validation. **Table S11.** Prediction metrics with standard errors, obtained from genomic BLUP (GBLUP), BayesB, elastic net (ENET), gradient boosting machine (GBM), and stacking ensemble (Stack) for liver function/hepatic damage for batch-out cross-validation. **Table S12.** Prediction metrics with standard errors, obtained from genomic BLUP (GBLUP), BayesB, elastic net (ENET), gradient boosting machine (GBM), and stacking ensemble (Stack) for oxidative stress metabolites for batch-out cross-validation. **Table S13.** Prediction metrics with standard errors, obtained from genomic BLUP (GBLUP), BayesB, elastic net (ENET), gradient boosting machine (GBM), and stacking ensemble (Stack) for inflammation/innate immunity for batch-out cross-validation. **Table S14.** Prediction metrics with standard errors, obtained from genomic BLUP (GBLUP), BayesB, elastic net (ENET), gradient boosting machine (GBM), and stacking ensemble (Stack) for blood minerals for batch-out cross-validation. **Table S15.** Prediction fit parameters including standard errors, considering the top 1500 SNP markers ranked by a GBM, obtained from genomic BLUP (GBLUP), Bayesian B (BayesB), elastic net (ENET), gradient boosting machine (GBM) and stacking ensemble (Stack) for energy-related metabolites. **Table S16.** Prediction fit parameters including standard errors, considering the top 1500 SNPs ranked by a GBM, obtained from genomic BLUP (GBLUP), Bayesian B (BayesB), elastic net (ENET), gradient boosting machine (GBM) and stacking ensemble (Stack) for liver function/hepatic damage. **Table S17.** Prediction fit parameters including standard errors, considering the top 1500 SNPs ranked by a GBM, obtained from genomic BLUP (GBLUP), Bayesian B (BayesB), elastic net (ENET), gradient boosting machine (GBM) and stacking ensemble (Stack) for oxidative stress metabolites. **Table S18.** Prediction fit parameters including standard errors, considering the top 1500 SNPs ranked by a GBM, obtained from genomic BLUP (GBLUP), Bayesian B (BayesB), elastic net (ENET), gradient boosting machine (GBM) and stacking ensemble (Stack) for inflammation/innate immunity. **Table S19.** Prediction fit parameters including standard errors, considering the top 1500 SNPs ranked by a GBM, obtained from genomic BLUP (GBLUP), Bayesian B (BayesB), elastic net (ENET), gradient boosting machine (GBM) and stacking ensemble (Stack) for minerals in blood.

## Data Availability

The phenotypic and genotypic information are available for academic use from the authors upon reasonable request (contacting the researcher Alessio Cecchinato e-mail: alessio.cecchinato@unipd.it).
