# Supplementary material for: Genomic prediction of blood biomarkers of metabolic disorders in Holstein cattle using parametric and nonparametric models
Source: Genet Sel Evol. 2024 Apr 29;56:31. doi: 10.1186/s12711-024-00903-9 (PMC11057143; doi:10.1186/s12711-024-00903-9)
Supplement: Supplementary file 1 — Additional file 1: Figure S1. Principal component analysis based on SNPs (a) and genomic relationship matrix (b) for Holstein cows. Principal component analysis of animals based on the first two principal components based on SNPs to evaluate the extent of the population structure (a) and genomic relationship matrix (b) in the Holstein cows. The colors represent the tenfold used in the cross-validation of genomic breeding values. Figure S2. Distribution of phenotypic values for energy-related metabolites: BHBA – β-hydroxybutyric acid; Cholest – cholesterol; CREA – creatinine; GLU – Glu – glucose; NEFA – non-esterified fatty acids and urea. Figure S3. Distribution of phenotypic values for liver function and hepatic damage, (a) and oxidative stress (b). Distribution of phenotypic values of blood metabolites related to liver function and hepatic damage (a): ALB – albumin; ALP – alkaline phosphatase; AST – aspartate aminotransferase; BILt – total bilirubin; GGT – γ -glutamyl transferase; and PON – paraoxonase and oxidative stress (b): AOPP – advanced oxidation protein products; FRAP – ferric reducing antioxidant power; RMT – total reactive oxygen metabolites; SHp – thiol groups. Figure S4. Distribution of phenotypic values for inflammation/innate immunity response (a) and mineral (b). Distribution of phenotypic values for blood metabolites related to inflammation/innate immunity (a): Hapto – haptoglobin; CuCp – ceruloplasmin; GLOB – globulins; MPO – myeloperoxidase; PROTt – total protein and mineral (b): CA – calcium; CL – chlorine; K – potassium; MG – magnesium; Na – sodium; P – phosphorus and Zn – zinc. Figure S5. Dominance (${{\text{d}}}^{2}$d2), additive-by-additive epistasis ( [file 12711_2024_903_MOESM1_ESM.docx]

# Additional file 1

**Figure S1** Principal component analysis of animals based on the first two principal components based on SNP marker to evaluate the extent of the population structure (a) and genomic relationship matrix (b) in the Holstein cows. The fold colors represent the tenfolds used in the cross-validation of genomic breeding value.


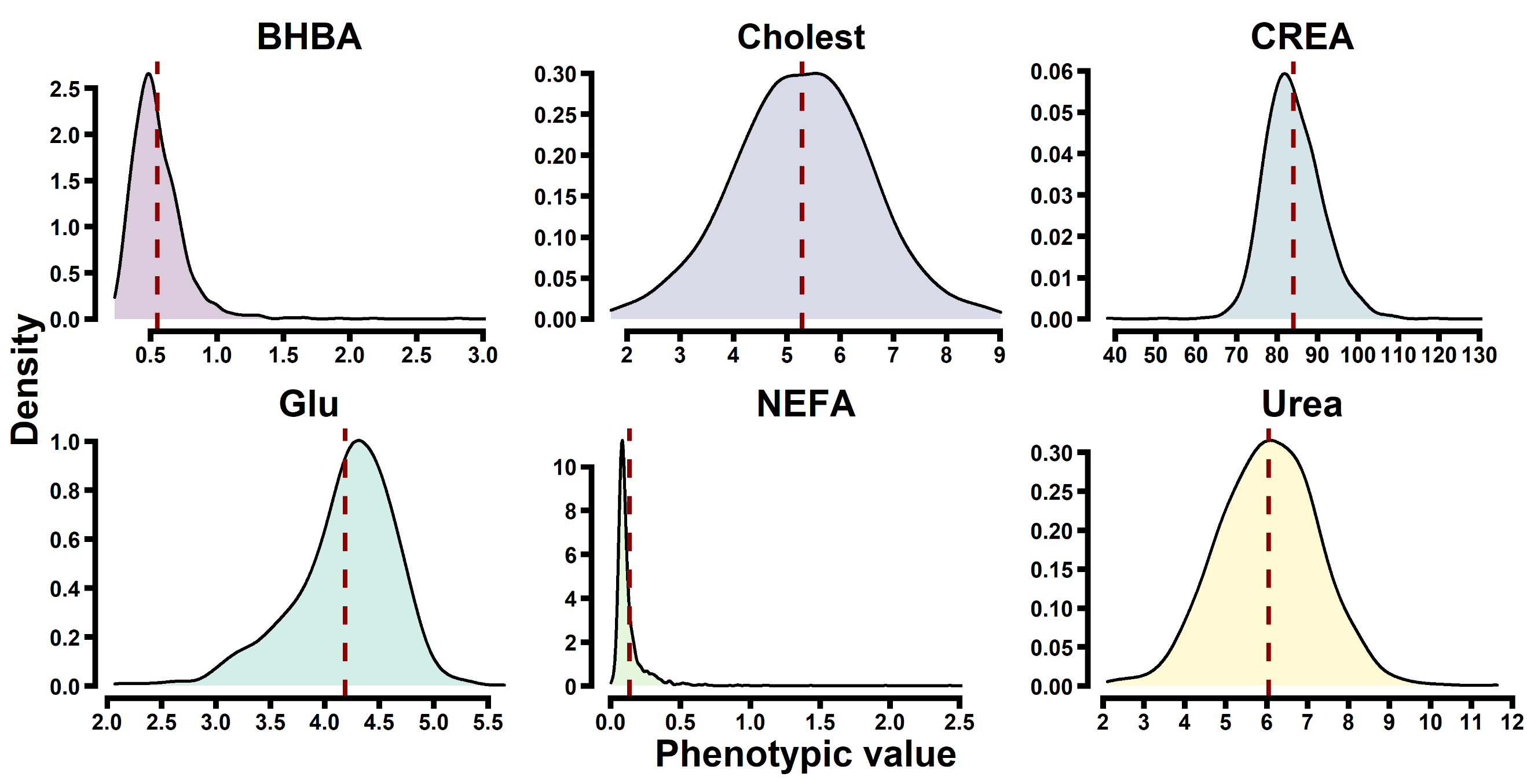


**Figure S2** Distribution of phenotypic values for energy-related metabolites: BHBA – β-hydroxybutyric acid; Cholest – Cholesterol; CREA – Creatinine; GLU – Glu – Glucose; NEFA – nonesterified fatty acids and Urea.


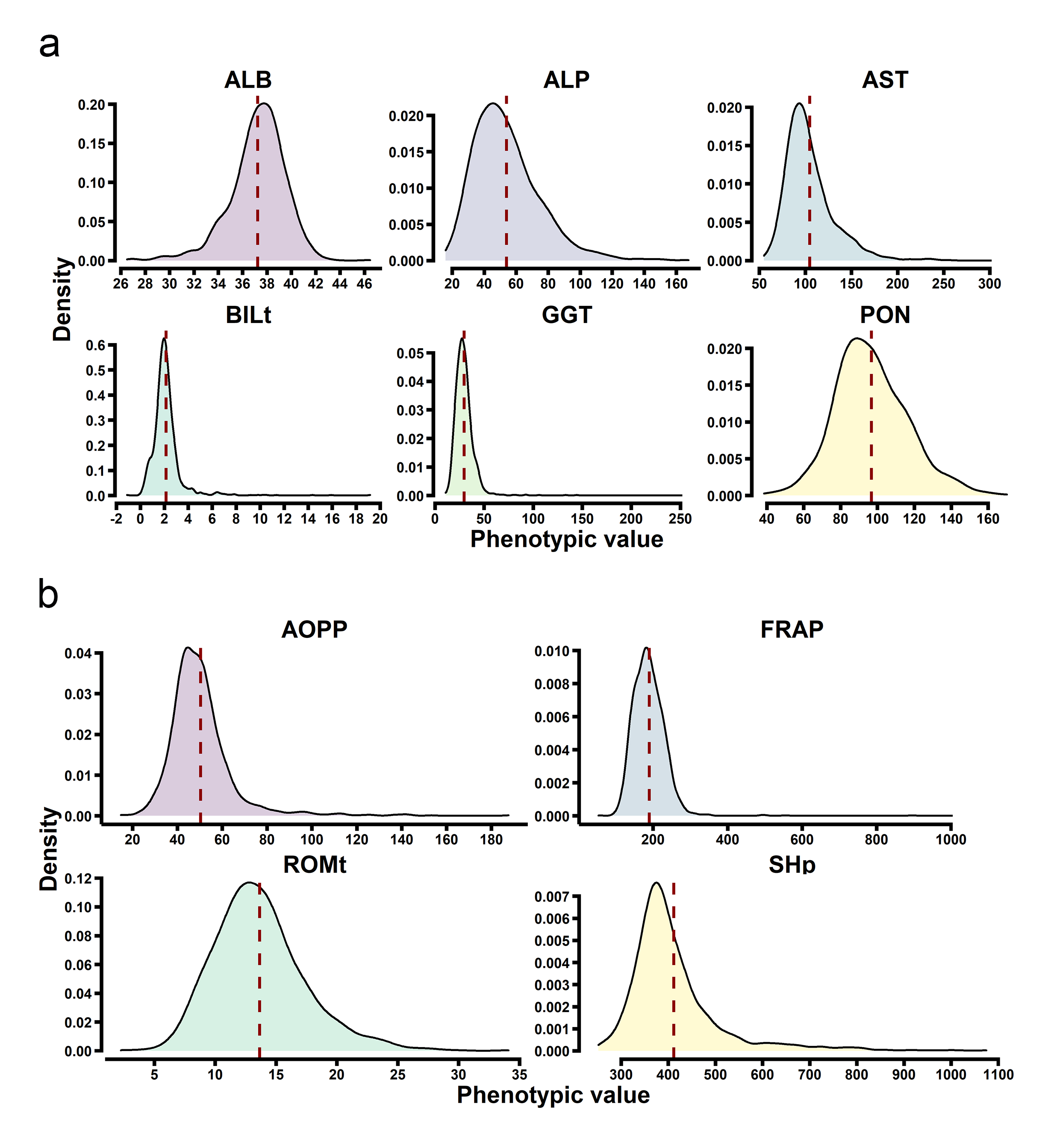


**Figure S3** Distribution of phenotypic values for blood metabolites related to liver function and hepatic damage [a]: ALB – Albumin; ALP – alkaline phosphatase; AST – aspartate aminotransferase; BILt – total bilirubin; GGT – γ -glutamyl transferase; and PON – Paraoxonase and oxidative stress [b]: AOPP – advanced oxidation protein products; FRAP – ferric reducing antioxidant power; RMT – total reactive oxygen metabolites; SHp – thiolic groups.


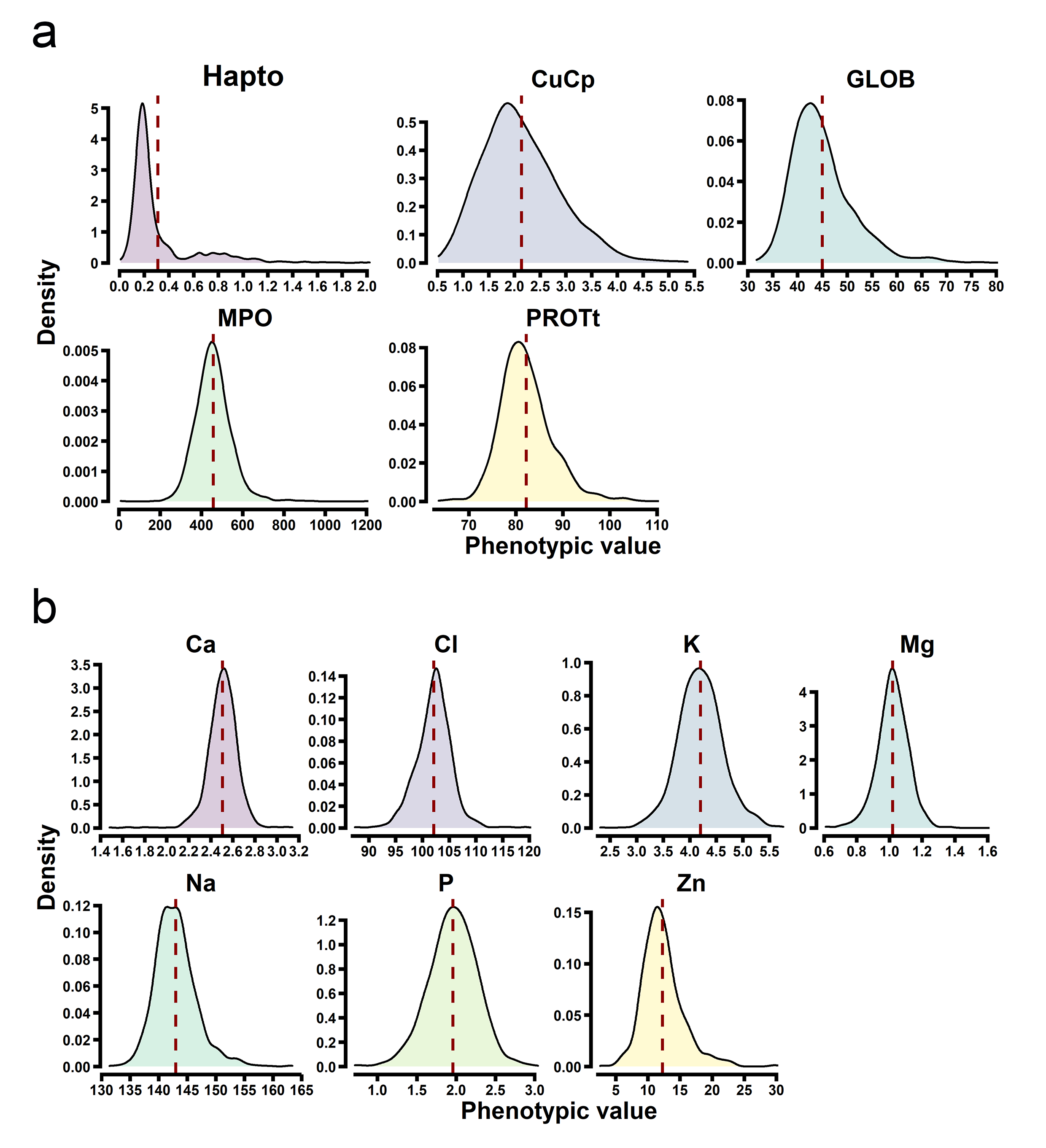


**Figure S4** Distribution of phenotypic values for blood metabolites related to inflammation/innate immunity [a]: Hapto – haptoglobin; Cucp – ceruloplasmin; GLOB – globulins; MPO – myeloperoxidase; PROTt – total protein and mineral [b]: CA – calcium; CL – chlorine; K – potassium; MG – magnesium; Na – sodium; P – phosphorus and Zn – zinc.

**
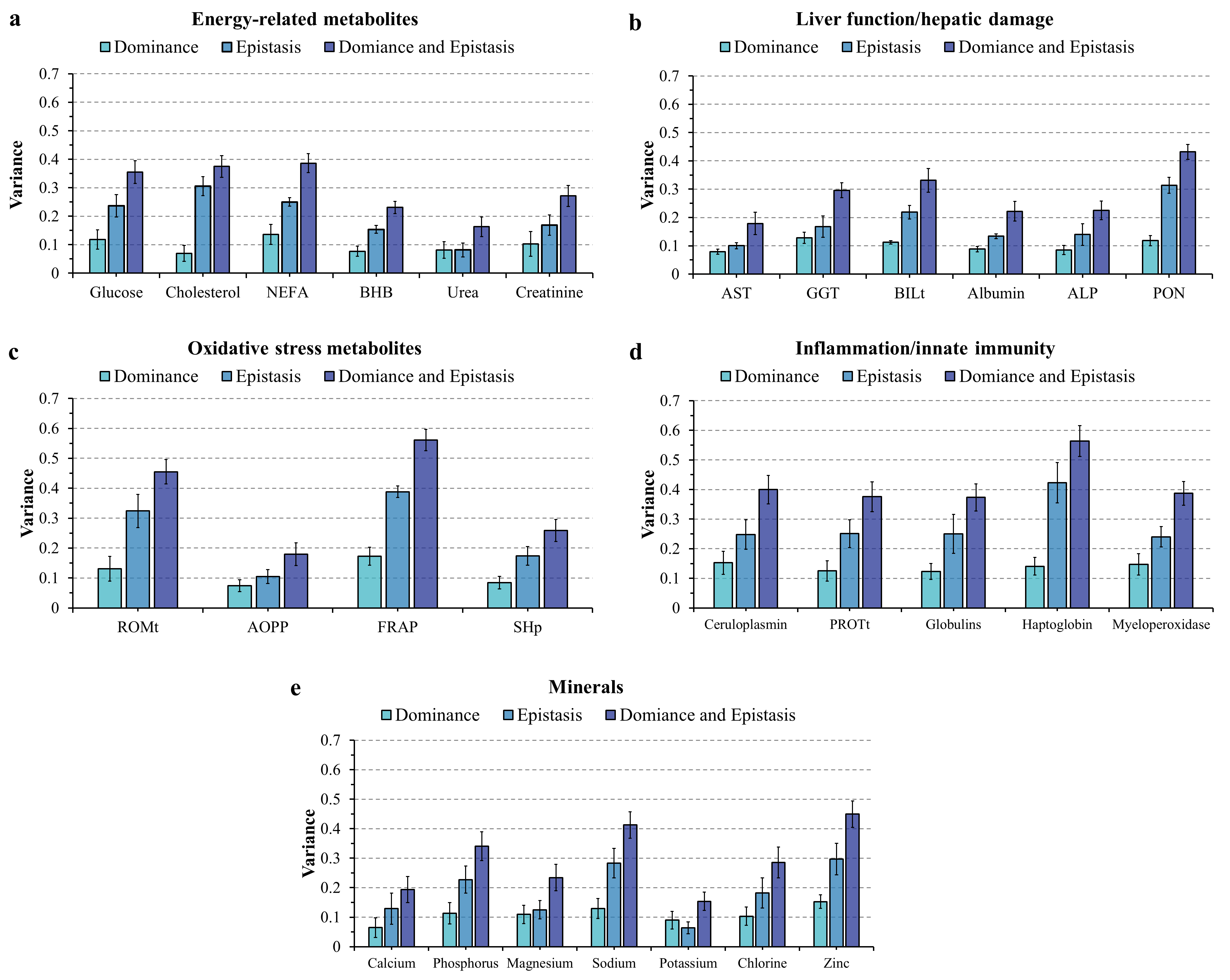
**

**Figure S5** Dominance ($d^{2}$), additive-by-additive epistasis ($\mathrm{ep}_{\mathrm{aa}}^{2})$, and dominance and epistasis ($\mathrm{epd}^{2}$) contribution for blood metabolites variability estimated as a proportion of total phenotypic variance considered as $d^{2}=\sigma_{d}^{2}/(\sigma_{a}^{2}+ \sigma_{d}^{2}+\sigma_{\mathrm{ep}_{\mathrm{aa}}}^{2}+\sigma_{\mathrm{batch}}^{2}+\sigma_{e}^{2})$, $\mathrm{ep}_{\mathrm{aa}}^{2}=\sigma_{\mathrm{ep}_{\mathrm{aa}}}^{2}/(\sigma_{a}^{2}+ \sigma_{d}^{2}+\sigma_{\mathrm{ep}_{\mathrm{aa}}}^{2}+\sigma_{\mathrm{batch}}^{2}+\sigma_{e}^{2})$ and $\mathrm{epd}^{2}=(\sigma_{d}^{2}+\sigma_{\mathrm{ep}_{\mathrm{aa}}}^{2})/(\sigma_{a}^{2}+ \sigma_{d}^{2}+\sigma_{\mathrm{ep}_{\mathrm{aa}}}^{2}+\sigma_{\mathrm{batch}}^{2}+\sigma_{e}^{2})$ where $\sigma_{a}^{2}$, $\sigma_{d}^{2}$, $\sigma_{\mathrm{ep}_{\mathrm{aa}}}^{2}$ and $\sigma_{e}^{2}$ represents the additive genetic variance, dominance variance, additive-by-additive variance, and residual variance, respectively.


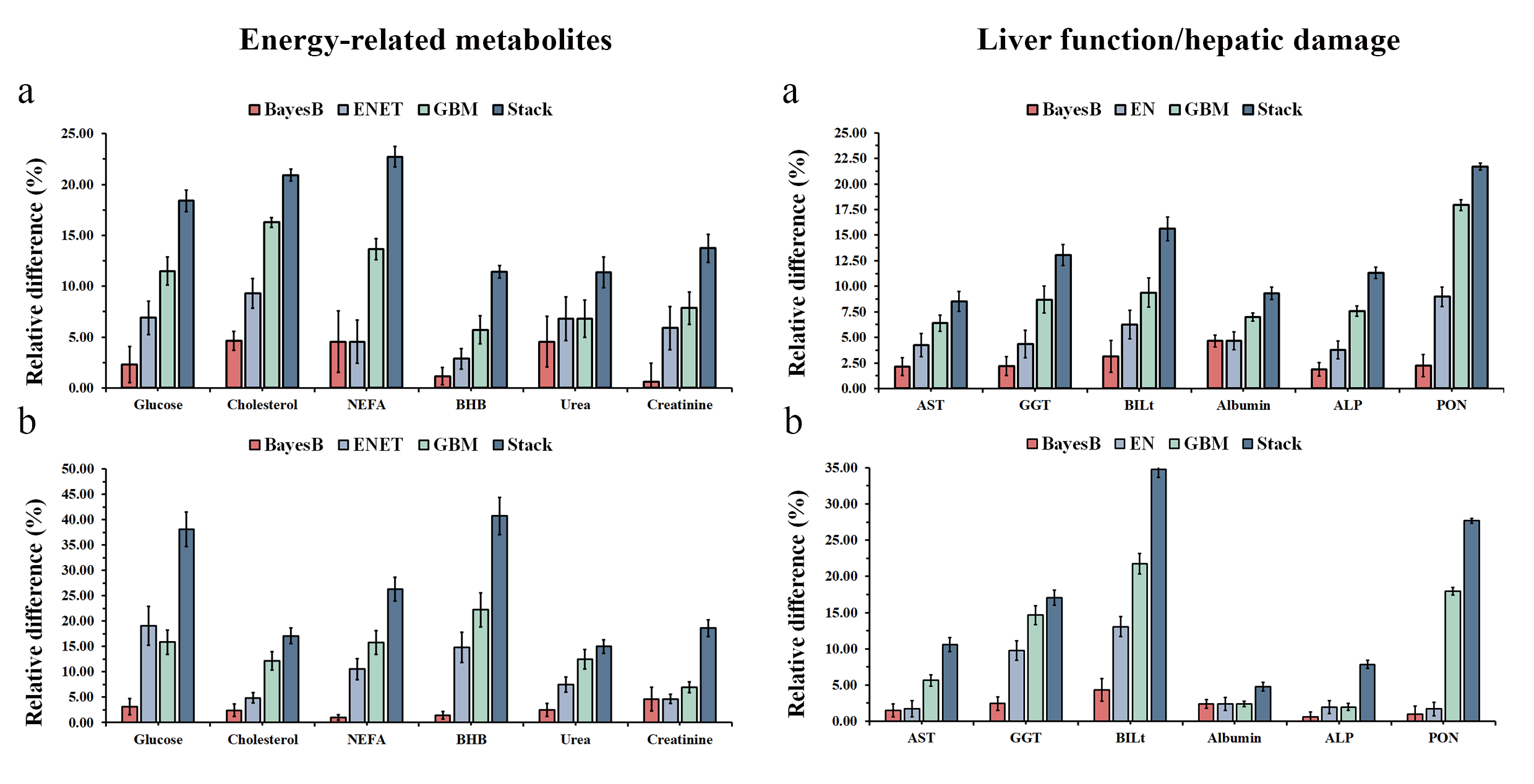


**Figure S6** Average of the relative difference (%) in predictive ability assessed by Pearson (a) and Spearman (b) correlation across 10-fold random cross-validation for the approaches BayesB, elastic net (EN), Gradient boosting machine (GBM) and stacking ensemble (Stack) against the genomic best linear unbiased prediction (GBLUP), for energy-related metabolites and liver function/hepatic damage blood metabolites in Holstein cows. Data are shown as mean ± SD (black error bar line). NEFA - non-esterified fatty acids; BHB - β-hydroxybutyric acid; AST - aspartate aminotransferase; GGT - γ-glutamyl transferase; BILt - total bilirubin; ALP - alkaline phosphatase and PON – Paraoxonase.


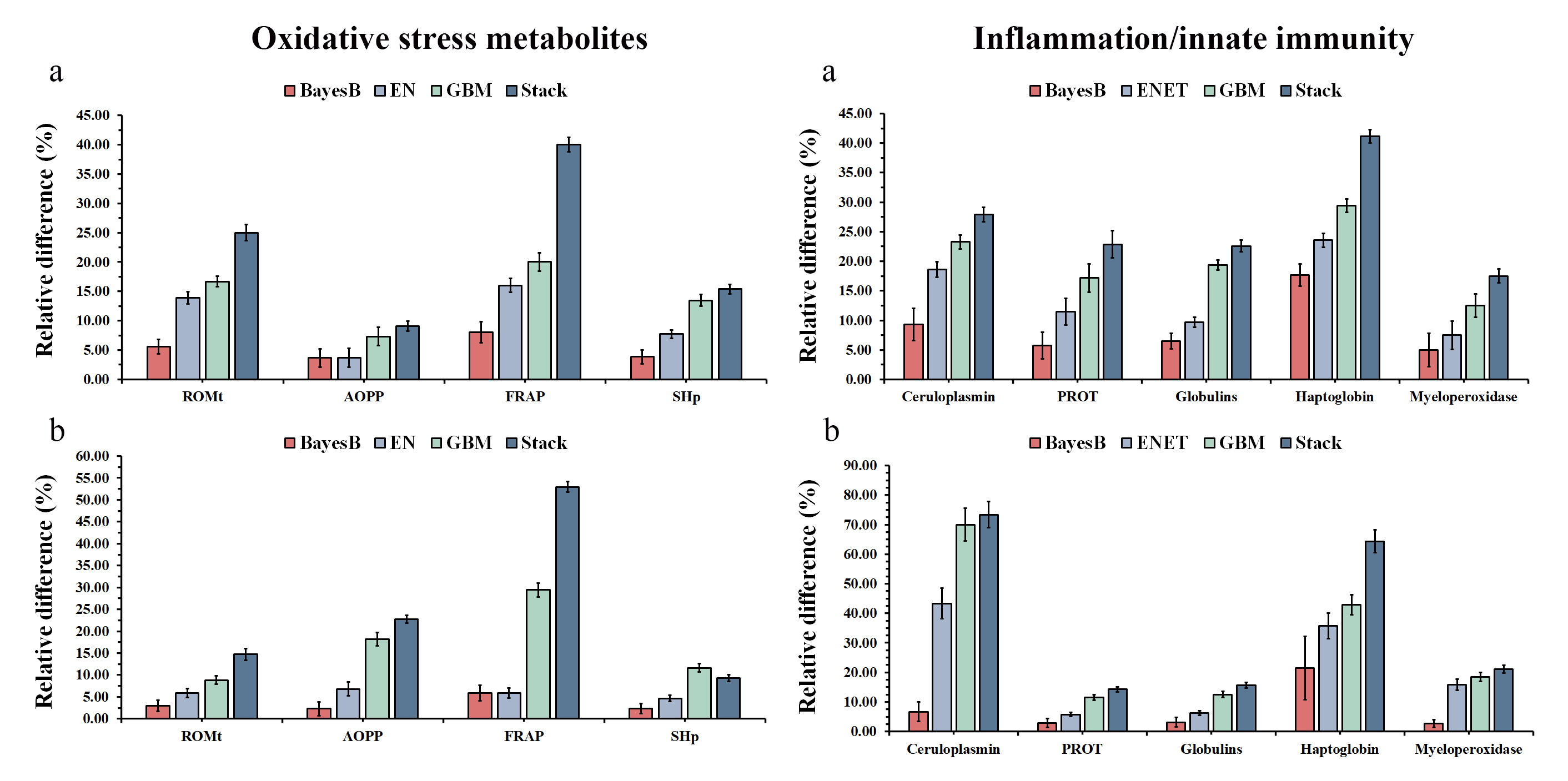


**Figure S7** Average of the relative difference (%) in predictive ability assessed by Pearson (a) and Spearman (b) correlation across 10-fold random cross-validation for the approaches BayesB, elastic net (ENET), Gradient boosting machine (GBM) and stacking ensemble (Stack) against genomic best linear unbiased prediction (GBLUP), for oxidative stress and inflammation/innate immunity response blood metabolites in Holstein cows. Data are shown as mean ± SD (black error bar line). ROMt - total reactive oxygen metabolites; AOPP - advanced oxidation protein products; FRAP - ferric reducing antioxidant power; SHp - thiol groups; PROT - total proteins.


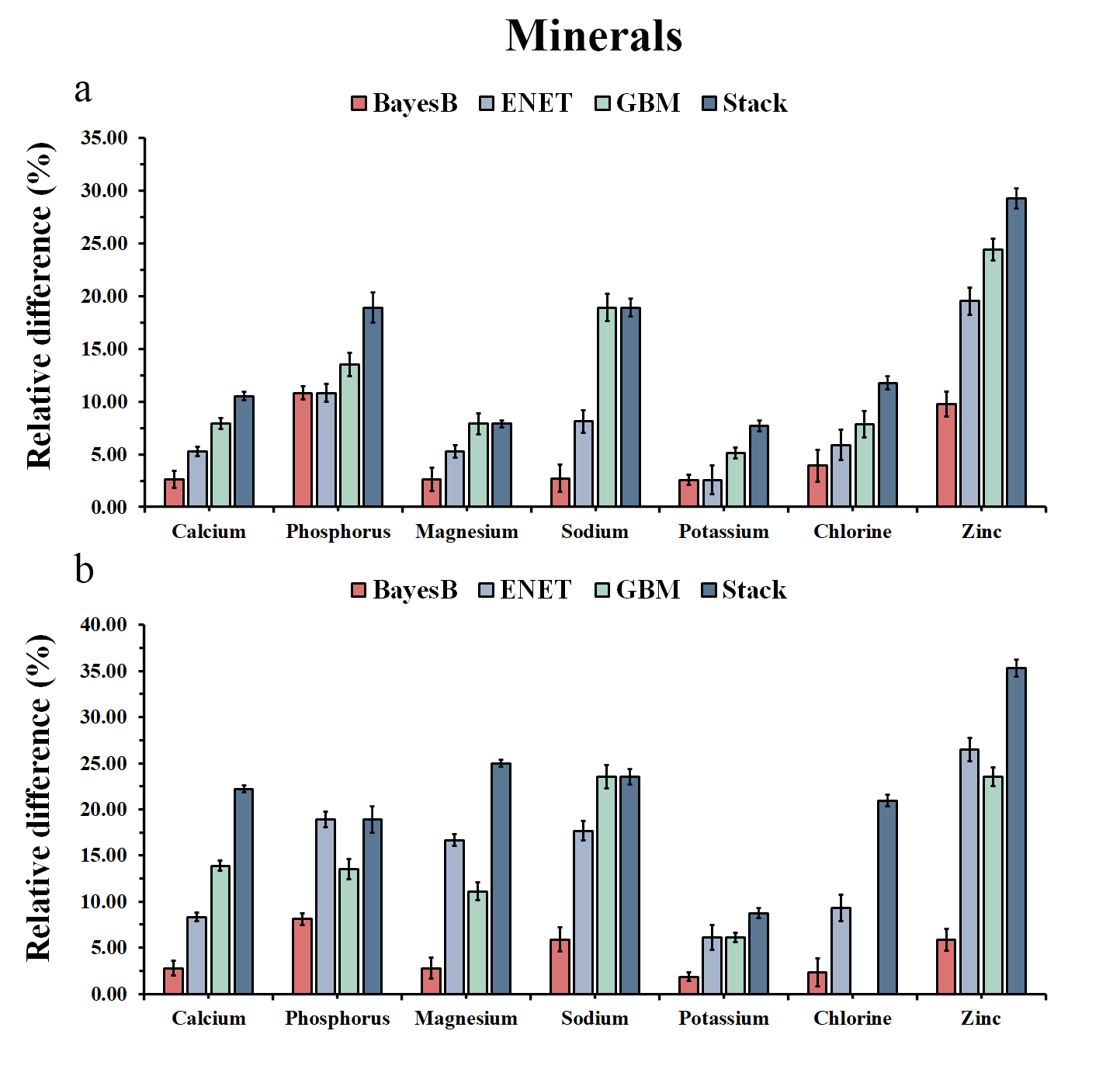


**Figure S8** Average of the relative difference (%) in predictive ability assessed by Pearson (A) and Spearman (B) correlation across 10-fold random cross-validation for the approaches BayesB, elastic net (ENET), Gradient boosting machine (GBM) and stacking ensemble (Stack) against genomic best linear unbiased prediction (GBLUP), for blood minerals in Holstein cows.


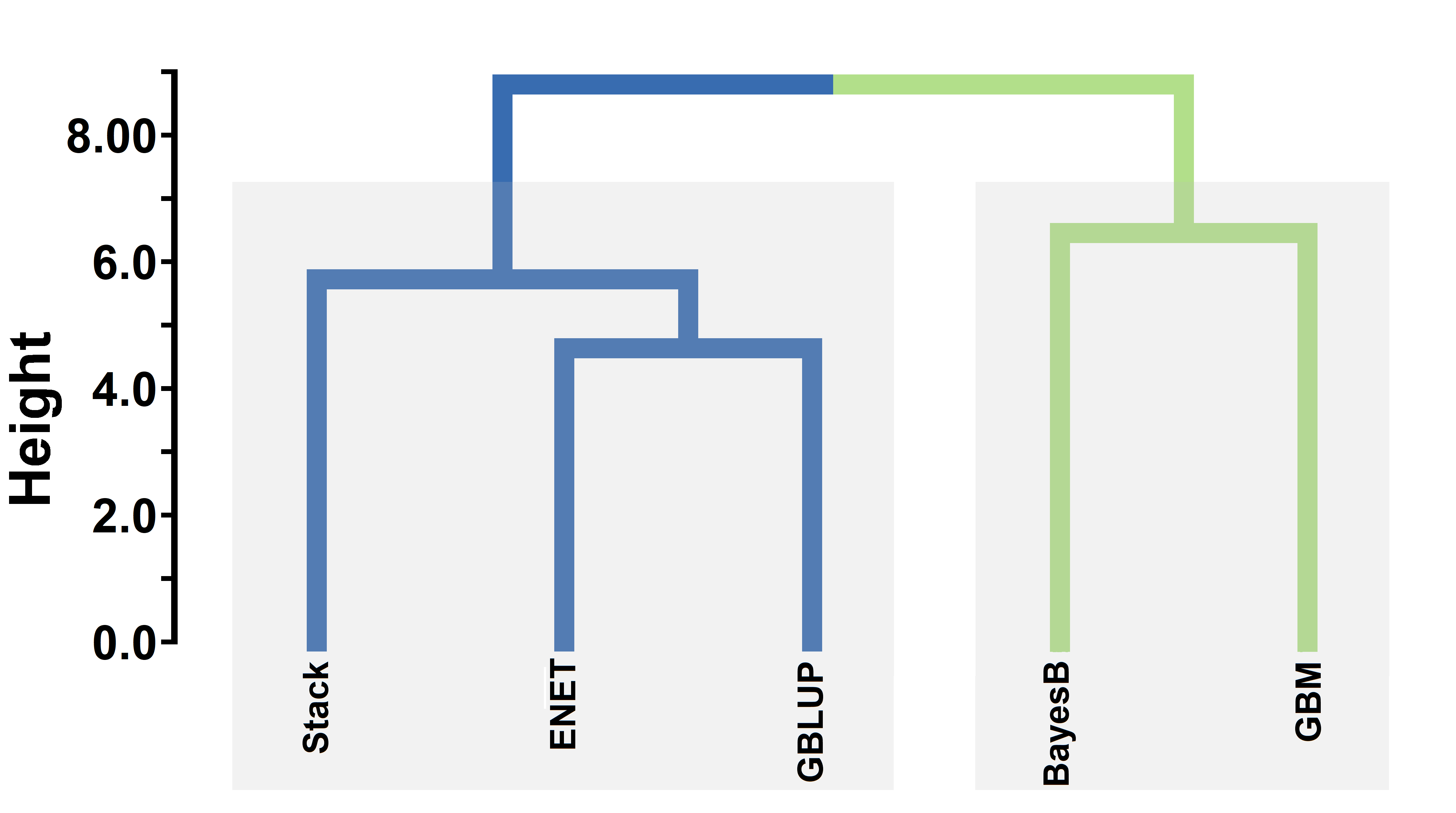


**Figure S9** Ward’s hierarchical clustering of models based on slope values for each model across all trait combinations.
