# Supplementary material for: Genomic prediction of blood biomarkers of metabolic disorders in Holstein cattle using parametric and nonparametric models
Source: Genet Sel Evol. 2024 Apr 29;56:31. doi: 10.1186/s12711-024-00903-9 (PMC11057143; doi:10.1186/s12711-024-00903-9)
Supplement: Supplementary file 2 — Additional file 2: Table S1. Descriptive statistics for blood metabolites related to energy, liver function/hepatic damage, oxidative stress, inflammation/innate immunity, and minerals (n = 1353). Table S2. Estimates of variance components, heritability (${{\text{h}}}^{2}$h2), and batch incidence (${{\text{h}}}_{{\text{batch}}}^{2}$hbatch2) for blood metabolites related to energy-related metabolites, liver function/hepatic damage, and oxidative stress metabolites. Table S3. Estimates of variance components, heritability (${{\text{h}}}^{2}$h2), and batch incidence (${{\text{h}}}_{{\text{batch}}}^{2}$hbatch2) for blood metabolites related to inflammation/innate immunity and minerals. Table S4. Estimates of variance components considering non-additive effects, heritability ( [file 12711_2024_903_MOESM2_ESM.docx]

# Additional file 2

**Table S1** Descriptive statistics for blood metabolites (n = 1353).

| **Blood metabolites^1^** | **Mean** | **SD** | **Min** | **Max** |
| --- | --- | --- | --- | --- |
| *Energy-related metabolites* | | | | |
| Glucose, mmol/l | 4.19 | 0.47 | 2.07 | 5.65 |
| Cholesterol, mmol/l | 5.29 | 1.27 | 1.70 | 9.02 |
| NEFA, mmol/l | 0.14 | 0.16 | 0.00 | 2.80 |
| BHB, mmol/l | 0.55 | 0.21 | 0.23 | 3.16 |
| Urea, mmol/l | 6.05 | 1.21 | 2.11 | 11.63 |
| Creatinine, µmol/l | 84.03 | 7.19 | 38.05 | 134.37 |
| *Liver function/hepatic damage* | | | | |
| AST, U/l | 104.72 | 26.88 | 55.06 | 318.61 |
| GGT, U/l | 29.75 | 11.46 | 10.66 | 258.33 |
| BILt, µmol/l | 2.15 | 1.29 | 0.11 | 19.16 |
| Albumin, g/l | 37.26 | 2.27 | 26.5 | 46.46 |
| ALP, U/l | 54.03 | 20.49 | 15.83 | 167.65 |
| PON, U/ml | 96.56 | 19.52 | 38.44 | 170.23 |
| *Oxidative stress metabolites* | | | | |
| ROMt, gH2O2/100ml | 13.71 | 4.19 | 2.24 | 80.14 |
| AOPP, µmol/l | 50.37 | 14.08 | 14.75 | 187.68 |
| FRAP, µmol/l | 190.00 | 56.09 | 53.51 | 1094.13 |
| SHp, µmol/l | 411.68 | 98.35 | 24.70 | 1074.49 |
| *Inflammation/innate immunity* | | | | |
| Ceruloplasmin, µmol/l | 2.14 | 0.76 | 0.52 | 5.37 |
| PROTt, g/l | 82.24 | 5.63 | 63.56 | 116.50 |
| Globulins, g/l | 44.98 | 6.33 | 31.73 | 81.84 |
| Haptoglobin, g/l | 0.31 | 0.28 | 0.01 | 2.02 |
| Myeloperoxidase, U/l | 457.56 | 80.89 | 8.23 | 1215.71 |
| *Minerals* | | | | |
| Calcium, mmol/l | 2.51 | 0.12 | 1.479 | 3.14 |
| Phosphorus, mmol/l | 1.96 | 0.30 | 0.71 | 3.04 |
| Magnesium, mmol/l | 1.02 | 0.09 | 0.61 | 1.73 |
| Sodium, mmol/l | 143.01 | 3.64 | 131.38 | 163.42 |
| Potassium, mmol/l | 4.20 | 0.42 | 2.32 | 5.76 |
| Chlorine, mmol/l | 102.14 | 3.19 | 87.34 | 121.58 |
| Zinc, µmol/l | 12.29 | 2.82 | 2.58 | 31.05 |

^1^NEFA - non-esterified fatty acids; BHB - β-hydroxybutyrate; AST - aspartate aminotransferase; GGT - γ -glutamyl transferase; BILt - total bilirubin; ALP - alkaline phosphatase; PON - paraoxonase; ROMt - total reactive oxygen metabolites; AOPP - advanced oxidation protein products; FRAP - ferric reducing antioxidant power; SHp - thiol groups; PROTt - total proteins.

N: number of records; SD: standard deviation; Min: minimum; Max: maximum.

**Table S2** Estimates (± SD) of variance components, heritability ($h^{2}$), and batch incidence ($h_{b\mathrm{atch}}^{2}$) for blood metabolites related to energy-related metabolites, liver function/hepatic damage and Oxidative stress metabolites.

| **Blood metabolites^1^** | $\boldsymbol{\sigma}_{\mathbf{batch}}^{\mathbf{2}}$ | $\boldsymbol{\sigma}_{\mathbf{a}}^{\mathbf{2}}$ | $\boldsymbol{\sigma}_{\mathbf{e}}^{\mathbf{2}}$ | $\mathbf{h}^{\mathbf{2}}$ | $\mathbf{h}_{\mathbf{b}\mathbf{atc}\mathbf{h}}^{\mathbf{2}}$ | **Acc^2^** |
| --- | --- | --- | --- | --- | --- | --- |
| Energy-related metabolites | | | | | | |
| Glucose, mmol/l | 0.046 ± 0.03 | 0.049 ± 0.009 | 0.081 ± 0.01 | 0.28 ± 0.05 | 0.26 ± 0.10 | 0.67 ± 0.09 |
| Cholesterol, mmol/l | 0.243 ± 0.09 | 0.463 ± 0.07 | 0.694 ± 0.05 | 0.33 ± 0.05 | 0.17 ± 0.09 | 0.69 ± 0.07 |
| NEFA, mmol/l | 0.003 ± 0.002 | 0.001 ± 0.0001 | 0.013 ± 0.001 | 0.06 ± 0.01 | 0.18 ± 0.08 | 0.70 ± 0.10 |
| BHB, mmol/l | 0.004 ± 0.01 | 0.005 ± 0.001 | 0.021 ± 0.001 | 0.17 ± 0.04 | 0.13 ± 0.12 | 0.76 ± 0.03 |
| Urea, mmol/l | 0.390 ± 0.17 | 0.427 ± 0.04 | 0.381 ± 0.03 | 0.36 ± 0.06 | 0.33 ± 0.15 | 0.65 ± 0.11 |
| Creatinine, µmol/l | 12.485 ± 4.29 | 10.893 ± 1.59 | 11.222 ± 1.11 | 0.32 ± 0.06 | 0.36 ± 0.16 | 0.63 ± 0.06 |
| Liver function/hepatic damage | | | | | | |
| AST, U/l | 56.476 ± 15.15 | 148.785 ± 24.15 | 271.96 ± 19.12 | 0.31 ± 0.05 | 0.12 ± 0.05 | 0.71 ± 0.11 |
| GGT, U/l | 4.178 ± 1.03 | 20.694 ± 5.52 | 42.121 ± 4.40 | 0.31 ± 0.06 | 0.06 ± 0.03 | 0.69 ± 0.10 |
| BILt, µmol/l | 0.435 ± 0.33 | 0.145 ± 0.04 | 0.709 ± 0.04 | 0.11 ± 0.05 | 0.34 ± 0.12 | 0.60 ± 0.17 |
| Albumin, g/l | 0.859 ± 0.21 | 0.905 ± 0.21 | 2.256 ± 0.17 | 0.23 ± 0.06 | 0.21 ± 0.11 | 0.63 ± 0.10 |
| ALP, U/l | 119.064 ± 61.94 | 93.774 ± 11.65 | 83.915 ± 7.83 | 0.32 ± 0.05 | 0.40 ± 0.14 | 0.76 ± 0.09 |
| PON, U/ml | 138.202 ± 82.71 | 98.941 ± 11.49 | 76.991 ± 7.54 | 0.32 ± 0.04 | 0.44 ± 0.15 | 0.77 ± 0.10 |
| Oxidative stress metabolites | | | | | | |
| ROMt, mgH_2_O_2_/100ml | 2.278 ± 1.09 | 2.49 ± 0.52 | 7.179 ± 0.46 | 0.22 ± 0.05 | 0.19 ± 0.07 | 0.68 ± 0.14 |
| AOPP, µmol/l | 36.56 ± 9.09 | 43.10 ± 6.67 | 38.434 ± 6.16 | 0.37 ± 0.05 | 0.31 ± 0.07 | 0.86 ± 0.06 |
| FRAP, µmol/l | 635.86 ± 78.35 | 198.34 ± 26.62 | 1294.483 ± 66.15 | 0.09 ± 0.02 | 0.30 ± 0.10 | 0.79 ± 0.08 |
| SHp, µmol/l | 1509.33 ± 233.33 | 1870.67 ± 226.31 | 2136 ± 128.36 | 0.34 ± 0.13 | 0.27 ± 0.05 | 0.83 ± 0.07 |

^1^ NEFA - non-esterified fatty acids; BHB - β-hydroxybutyrate; AST - aspartate aminotransferase; GGT - γ-glutamyl transferase; BILt - total bilirubin; ALP - alkaline phosphatase; PON - paraoxonase; ROMt - total reactive oxygen metabolites; AOPP - advanced oxidation protein products; FRAP - ferric reducing antioxidant power; SHp - thiol groups. $h^{2}$ – heritability computed as $\sigma_{a}^{2}/(\sigma_{a}^{2}+\sigma_{\mathrm{batch}}^{2}+\sigma_{e}^{2})$; $h_{b\mathrm{atch}}^{2}$ – herd incidence estimated as $\sigma_{\mathrm{batch}}^{2}/(\sigma_{a}^{2}+\sigma_{\mathrm{batch}}^{2}+\sigma_{e}^{2})$.

^2^Acc – accuracy of GEBV based on prediction error variance (PEV) as $\sqrt{1-PEV/\sigma_{a}^{2}}$ .

**Table S3** Estimates (± SD) of variance components, heritability ($h^{2}$), and batch incidence ($h_{b\mathrm{atch}}^{2}$) for blood metabolites related to Inflammation/innate immunity, and Minerals.

| **Blood metabolites^1^** | $\boldsymbol{\sigma}_{\mathbf{batch}}^{\mathbf{2}}$ | $\boldsymbol{\sigma}_{\mathbf{a}}^{\mathbf{2}}$ | $\boldsymbol{\sigma}_{\mathbf{e}}^{\mathbf{2}}$ | $\mathbf{h}^{\mathbf{2}}$ | $\mathbf{h}_{\mathbf{b}\mathbf{atc}\mathbf{h}}^{\mathbf{2}}$ | **Acc^2^** |
| --- | --- | --- | --- | --- | --- | --- |
| Inflammation/innate immunity | | | | | | |
| Ceruloplasmin, µmol/l | 0.05 ± 0.04 | 0.156 ± 0.03 | 0.228 ± 0.02 | 0.41 ± 0.06 | 0.11 ± 0.07 | 0.64 ± 0.14 |
| PROTt, g/l | 1.89 ± 1.10 | 5.000 ± 1.77 | 19.43 ± 1.57 | 0.21 ± 0.06 | 0.07 ± 0.04 | 0.59 ± 0.12 |
| Globulins, g/l | 3.04 ± 1.21 | 3.962 ± 1.31 | 19.194 ± 1.23 | 0.17 ± 0.05 | 0.12 ± 0.06 | 0.60 ± 0.13 |
| Haptoglobin, g/l | 0.02 ± 0.01 | 0.005 ± 0.001 | 0.032 ± 0.002 | 0.09 ± 0.02 | 0.35 ± 0.12 | 0.62 ± 0.08 |
| Myeloperoxidase, U/l | 1492.25 ± 562.00 | 704.7 ± 89.47 | 1832.8 ± 97.07 | 0.18 ± 0.06 | 0.37 ± 0.15 | 0.58 ± 0.18 |
| Minerals | | | | | | |
| Calcium, mmol/l | 0.002 ± 0.001 | 0.002 ± 0.0004 | 0.007 ± 0.0004 | 0.14 ± 0.04 | 0.21 ± 0.07 | 0.63 ± 0.14 |
| Phosphorus, mmol/l | 0.009 ± 0.006 | 0.01 ± 0.002 | 0.03 ± 0.0021 | 0.21 ± 0.05 | 0.18 ± 0.08 | 0.59 ± 0.17 |
| Magnesium, mmol/l | 0.001 ± 0.001 | 0.001 ± 0.0003 | 0.003 ± 0.0003 | 0.19 ± 0.06 | 0.21 ± 0.09 | 0.60 ± 0.17 |
| Sodium, mmol/l | 5.13 ± 3.11 | 1.28 ± 0.31 | 3.97 ± 0.23 | 0.12 ± 0.07 | 0.49 ± 0.12 | 0.65 ± 0.14 |
| Potassium, mmol/l | 0.05 ± 0.02 | 0.02 ± 0.003 | 0.07 ± 0.003 | 0.16 ± 0.04 | 0.36 ± 0.14 | 0.58 ± 0.18 |
| Chlorine, mmol/l | 1.98 ± 0.99 | 1.58 ± 0.13 | 3.84 ± 0.09 | 0.21 ± 0.07 | 0.27 ± 0.11 | 0.73 ± 0.12 |
| Zinc, µmol/l | 1.780 ± 0.58 | 1.56 ± 0.29 | 2.50 ± 0.18 | 0.27 ± 0.05 | 0.33 ± 0.12 | 0.72 ± 0.17 |

^1^PROTt – total proteins. $h^{2}$ – heritability computed as $\sigma_{a}^{2}/(\sigma_{a}^{2}+\sigma_{\mathrm{batch}}^{2}+\sigma_{e}^{2}); h_{b\mathrm{atch}}^{2}$ – herd incidence estimated as $\sigma_{\mathrm{batch}}^{2}/(\sigma_{a}^{2}+\sigma_{\mathrm{batch}}^{2}+\sigma_{e}^{2})$. ^2^Acc – accuracy of GEBV based on prediction error variance (PEV) as $\sqrt{1-PEV/\sigma_{a}^{2}}$ .

**Table S4** Estimates (± SD) of variance components considering dominance and additive-by-additive epistatic effect, heritability ($h^{2}$), batch incidence ($h_{b\mathrm{atch}}^{2}$), dominance ($d^{2}$), additive-by-additive epistasis ($\mathrm{ep}_{\mathrm{aa}}^{2})$, and dominance and epistasis ($\mathrm{epd}^{2}$) contribution for blood metabolites variability.

| **Blood metabolites^1^** | $\boldsymbol{\sigma}_{\mathbf{batch}}^{\mathbf{2}}$ | $\boldsymbol{\sigma}_{\mathbf{a}}^{\mathbf{2}}$ | $\boldsymbol{\sigma}_{\mathbf{d}}^{\mathbf{2}}$ | $\boldsymbol{\sigma}_{\boldsymbol{ep}}^{\mathbf{2}}$ | $\boldsymbol{\sigma}_{\mathbf{e}}^{\mathbf{2}}$ | $\mathbf{h}^{\mathbf{2}}$ | $\mathbf{h}_{\mathbf{b}\mathbf{atc}\mathbf{h}}^{\mathbf{2}}$ | $\mathbf{d}^{\mathbf{2}}$ | $\mathbf{ep}_{\mathbf{aa}}^{\mathbf{2}}$ |
| --- | --- | --- | --- | --- | --- | --- | --- | --- | --- |
| *Energy-related metabolites* | | | | | | | | | |
| Glucose, mmol/l | 0.02 ± 0.02 | 0.039 ± 0.008 | 0.02 ± 0.006 | 0.04 ± 0.01 | 0.05 ± 0.007 | 0.36 ± 0.05 | 0.18 ± 0.07 | 0.12 ± 0.03 | 0.24 ± 0.04 |
| Cholesterol, mmol/l | 0.15 ± 0.16 | 0.47 ± 0.09 | 0.15 ± 0.054 | 0.66 ± 0.07 | 0.73 ± 0.08 | 0.35 ± 0.05 | 0.11 ± 0.05 | 0.07 ± 0.02 | 0.31 ± 0.03 |
| NEFA, mmol/l | 0.002 ± 0.002 | 0.0015 ± 0.001 | 0.003 ± 0.001 | 0.0055 ± 0.001 | 0.01 ± 0.001 | 0.11 ± 0.02 | 0.15 ± 0.05 | 0.14 ± 0.03 | 0.25 ± 0.02 |
| BHB, mmol/l | 0.005 ± 0.005 | 0.005 ± 0.001 | 0.004 ± 0.001 | 0.008 ± 0.001 | 0.03 ± 0.002 | 0.13 ± 0.03 | 0.13 ± 0.06 | 0.08 ± 0.02 | 0.15 ± 0.01 |
| Urea, mmol/l | 0.13 ± 0.13 | 0.41 ± 0.06 | 0.11 ± 0.042 | 0.11 ± 0.04 | 0.59 ± 0.06 | 0.36 ± 0.05 | 0.12 ± 0.06 | 0.08 ± 0.03 | 0.08 ± 0.02 |
| Creatinine, µmol/l | 4.63 ± 4.40 | 9.03 ± 0.99 | 4.45 ± 2.58 | 7.29 ± 4.49 | 17.83 ± 3.01 | 0.29 ± 0.05 | 0.15 ± 0.05 | 0.10 ± 0.04 | 0.17 ± 0.06 |
| *Liver function/hepatic damage* | | | | | | | | | |
| AST, U/l | 64.66 ± 43.76 | 132.61 ± 39.04 | 49.75 ± 29.244 | 63.01 ± 61.41 | 319.77 ± 46.38 | 0.26 ± 0.05 | 0.13 ± 0.05 | 0.08 ± 0.01 | 0.10 ± 0.011 |
| GGT, U/l | 2.74 ± 1.70 | 18.52 ± 3.16 | 9.74 ± 0.994 | 12.71 ± 1.66 | 32.08 ± 3.78 | 0.35 ± 0.02 | 0.05 ± 0.06 | 0.13 ± 0.02 | 0.17 ± 0.038 |
| BILt, µmol/l | 0.13 ± 0.15 | 0.14 ± 0.05 | 0.18 ± 0.11 | 0.35 ± 0.14 | 0.80 ± 0.15 | 0.13 ± 0.03 | 0.12 ± 0.06 | 0.11 ± 0.01 | 0.22 ± 0.02 |
| Albumin, g/l | 0.475 ± 0.49 | 0.78 ± 0.25 | 0.47 ± 0.23 | 0.71 ± 0.37 | 2.88 ± 0.38 | 0.19 ± 0.05 | 0.12 ± 0.07 | 0.09 ± 0.004 | 0.13 ± 0.05 |
| ALP, U/l | 40.96 ± 20.25 | 98.54 ± 19.24 | 28.08 ± 8.15 | 45.96 ± 14.40 | 115.27 ± 14.43 | 0.39 ± 0.07 | 0.16 ± 0.05 | 0.09 ± 0.002 | 0.14 ± 0.03 |
| PON, U/ml | 38.69 ± 17.88 | 96.55 ± 23.12 | 58.59 ± 7.91 | 155.15 ± 12.55 | 145.92 ± 15.31 | 0.34 ± 0.08 | 0.14 ± 0.05 | 0.12 ± 0.01 | 0.31 ± 0.02 |
| *Oxidative stress metabolites* | | | | | | | | | |
| ROMt, mgH2O2/100ml | 1.08 ± 1.74 | 2.11 ± 0.67 | 1.87 ± 0.63 | 4.62 ± 1.14 | 4.59 ± 1.16 | 0.27 ± 0.05 | 0.14 ± 0.05 | 0.13 ± 0.04 | 0.32 ± 0.05 |
| AOPP, µmol/l | 26.27 ± 24.03 | 39.29 ± 5.11 | 9.17 ± 3.27 | 12.88 ± 8.083 | 35.23 ± 4.01 | 0.39 ± 0.02 | 0.26 ± 0.06 | 0.07 ± 0.02 | 0.10 ± 0.04 |
| FRAP, µmol/l | 274.42 ± 90.79 | 205.90 ± 87.49 | 647.42 ± 90.77 | 1452.09 ± 57.84 | 1161.41 ± 151.07 | 0.13 ± 0.03 | 0.17 ± 0.06 | 0.17 ± 0.03 | 0.39 ± 0.01 |
| SHp, µmol/l | 1043.18 ± 96.47 | 1521.16 ± 161.18 | 437.75 ± 22.18 | 897.75 ± 82.42 | 1262.76 ± 130.36 | 0.40 ± 0.04 | 0.27 ± 0.07 | 0.08 ± 0.02 | 0.17 ± 0.03 |
| *Inflammation/innate immunity* | | | | | | | | | |
| Ceruloplasmin, µmol/l | 0.05 ± 0.05 | 0.12 ± 0.02 | 0.08 ± 0.02 | 0.13 ± 0.02 | 0.14 ± 0.02 | 0.40 ± 0.05 | 0.17 ± 0.05 | 0.15 ± 0.03 | 0.25 ± 0.05 |
| PROTt, g/l | 2.83 ± 3.33 | 2.42 ± 0.82 | 3.46 ± 0.96 | 6.95 ± 0.84 | 10.05 ± 2.33 | 0.15 ± 0.02 | 0.18 ± 0.06 | 0.13 ± 0.03 | 0.27 ± 0.04 |
| Globulins, g/l | 3.58 ± 3.46 | 3.97 ± 1.28 | 4.84 ± 1.07 | 9.85 ± 1.13 | 17.09 ± 2.95 | 0.16 ± 0.03 | 0.14 ± 0.06 | 0.12 ± 0.02 | 0.25 ± 0.06 |
| Haptoglobin, g/l | 0.007 ± 0.007 | 0.005 ± 0.001 | 0.02 ± 0.004 | 0.06 ± 0.003 | 0.05 ± 0.005 | 0.07 ± 0.04 | 0.10 ± 0.07 | 0.17 ± 0.03 | 0.40 ± 0.06 |
| Myeloperoxidase, U/l | 509.92 ± 56.99 | 693.10 ± 113.88 | 650.21 ± 81.26 | 1064.58 ± 513.41 | 1511.67 ± 364.54 | 0.26 ± 0.07 | 0.19 ± 0.05 | 0.15 ± 0.03 | 0.24 ± 0.03 |
| *Minerals* | | | | | | | | | |
| Calcium, mmol/l | 0.001 ± 0.001 | 0.001 ± 0.0004 | 0.001 ± 0.001 | 0.002 ± 0.001 | 0.01 ± 0.001 | 0.15 ± 0.05 | 0.17 ± 0.05 | 0.10 ± 0.03 | 0.14 ± 0.05 |
| Phosphorus, mmol/l | 0.008 ± 0.01 | 0.01 ± 0.004 | 0.01 ± 0.005 | 0.02 ± 0.006 | 0.04 ± 0.007 | 0.20 ± 0.02 | 0.15 ± 0.06 | 0.12 ± 0.03 | 0.29 ± 0.04 |
| Magnesium, mmol/l | 0.0009 ± 0.001 | 0.001 ± 0.001 | 0.0007 ± 0.0004 | 0.0008 ± 0.0009 | 0.003 ± 0.001 | 0.20 ± 0.03 | 0.18 ± 0.06 | 0.11 ± 0.03 | 0.13 ± 0.06 |
| Sodium, mmol/l | 1.34 ± 0.32 | 1.03 ± 0.45 | 1.12 ± 0.33 | 2.45 ± 0.74 | 2.71 ± 0.44 | 0.20 ± 0.04 | 0.26 ± 0.07 | 0.13 ± 0.03 | 0.28 ± 0.05 |
| Potassium, mmol/l | 0.02 ± 0.02 | 0.022 ± 0.01 | 0.014 ± 0.005 | 0.01 ± 0.004 | 0.09 ± 0.007 | 0.16 ± 0.07 | 0.16 ± 0.05 | 0.09 ± 0.03 | 0.07 ± 0.02 |
| Chlorine, mmol/l | 1.05 ± 1.16 | 1.58 ± 0.63 | 0.83 ± 0.29 | 1.47 ± 0.79 | 3.12 ± 0.50 | 0.27 ± 0.07 | 0.18 ± 0.05 | 0.10 ± 0.03 | 0.18 ± 0.05 |
| Zinc, µmol/l | 0.84 ± 0.06 | 1.05 ± 0.05 | 0.98 ± 0.22 | 1.91 ± 0.82 | 1.65 ± 0.28 | 0.30 ± 1.07 | 0.24 ± 1.05 | 0.15 ± 0.02 | 0.30 ± 0.06 |

^1^ NEFA - non-esterified fatty acids; BHB - β-hydroxybutyrate; AST - aspartate aminotransferase; GGT - γ-glutamyl transferase; BILt - total bilirubin; ALP - alkaline phosphatase; PON - paraoxonase; ROMt - total reactive oxygen metabolites; AOPP - advanced oxidation protein products; FRAP - ferric reducing antioxidant power; SHp - thiol groups; PROTt – total proteins.

$h^{2}$ – heritability computed as $\sigma_{a}^{2}/(\sigma_{a}^{2}+\sigma_{\mathrm{batch}}^{2}+\sigma_{e}^{2})$; $h_{b\mathrm{atch}}^{2}$ – herd incidence estimated as $\sigma_{\mathrm{batch}}^{2}/(\sigma_{a}^{2}+\sigma_{\mathrm{batch}}^{2}+\sigma_{e}^{2})$, $d^{2}=\sigma_{d}^{2}/(\sigma_{a}^{2}+ \sigma_{d}^{2}+\sigma_{\mathrm{ep}_{\mathrm{aa}}}^{2}+\sigma_{\mathrm{batch}}^{2}+\sigma_{e}^{2})$, $\mathrm{ep}_{\mathrm{aa}}^{2}=\sigma_{\mathrm{ep}_{\mathrm{aa}}}^{2}/(\sigma_{a}^{2}+ \sigma_{d}^{2}+\sigma_{\mathrm{ep}_{\mathrm{aa}}}^{2}+\sigma_{\mathrm{batch}}^{2}+\sigma_{e}^{2})$ and $\mathrm{epd}^{2}=(\sigma_{d}^{2}+\sigma_{\mathrm{ep}_{\mathrm{aa}}}^{2})/(\sigma_{a}^{2}+ \sigma_{d}^{2}+\sigma_{\mathrm{ep}_{\mathrm{aa}}}^{2}+\sigma_{\mathrm{batch}}^{2}+\sigma_{e}^{2})$ where $\sigma_{a}^{2}$, $\sigma_{d}^{2}$, $\sigma_{\mathrm{ep}_{\mathrm{aa}}}^{2}$ and $\sigma_{e}^{2}$ represents the additive genetic variance, dominance variance, additive-by-additive variance, and residual variance, respectively.

**Table S5** Prediction metrics with standard errors, obtained from genomic BLUP (GBLUP), BayesB, elastic net (ENET), gradient boosting machine (GBM), and stacking ensemble (Stack) for energy-related metabolites for tenfold cross-validation.

| **Model** | **Metrics** | **Glucose** | **Cholesterol** | **NEFA** | **BHB** | **Urea** | **Creatinine** |
| --- | --- | --- | --- | --- | --- | --- | --- |
| Gblup | r_pearson_ | 0.44 ± 0.05 | 0.43 ± 0.02 | 0.22 ± 0.10 | 0.35 ± 0.02 | 0.44 ± 0.07 | 0.51 ± 0.04 |
|  | r_spearman_ | 0.32 ± 0.05 | 0.41 ± 0.03 | 0.19 ± 0.06 | 0.27 ± 0.03 | 0.40 ± 0.04 | 0.43 ± 0.06 |
|  | RMSE | 0.41 ± 0.02 | 1.14 ± 0.08 | 0.20 ± 0.02 | 0.94 ± 0.05 | 0.26 ± 0.01 | 5.34 ± 0.38 |
|  | slope | 1.08 ± 0.05 | 1.11 ± 0.08 | 1.01 ± 0.07 | 1.02 ± 0.01 | 1.04 ± 0.05 | 1.03 ± 0.03 |
| BayesB | r_pearson_ | 0.45 ± 0.04 | 0.45 ± 0.02 | 0.23 ± 0.07 | 0.35 ± 0.03 | 0.46 ± 0.05 | 0.51 ± 0.04 |
|  | r_spearman_ | 0.33 ± 0.05 | 0.42 ± 0.03 | 0.20 ± 0.06 | 0.27 ± 0.02 | 0.41 ± 0.03 | 0.45 ± 0.05 |
|  | RMSE | 0.42 ± 0.01 | 1.05 ± 0.06 | 0.19 ± 0.02 | 0.93 ± 0.04 | 0.26 ± 0.01 | 5.32 ± 0.38 |
|  | slope | 1.08 ± 0.05 | 1.05 ± 0.06 | 1.11 ± 0.06 | 1.03 ± 0.01 | 1.05 ± 0.09 | 1.03 ± 0.03 |
| ENET | r_pearson_ | 0.47 ± 0.04 | 0.47 ± 0.03 | 0.23 ± 0.05 | 0.36 ± 0.02 | 0.47 ± 0.05 | 0.54 ± 0.05 |
|  | r_spearman_ | 0.38 ± 0.07 | 0.43 ± 0.06 | 0.21 ± 0.03 | 0.31 ± 0.03 | 0.38 ± 0.03 | 0.45 ± 0.05 |
|  | RMSE | 0.45 ± 0.02 | 1.21 ± 0.10 | 0.18 ± 0.01 | 0.92 ± 0.03 | 0.27 ± 0.01 | 5.29 ± 0.25 |
|  | slope | 1.07 ± 0.06 | 1.04 ± 0.08 | 1.04 ± 0.04 | 1.07 ± 0.04 | 1.07 ± 0.07 | 1.04 ± 0.03 |
| GBM | r_pearson_ | 0.49 ± 0.03 | 0.50 ± 0.02 | 0.25 ± 0.03 | 0.37 ± 0.03 | 0.47 ± 0.05 | 0.55 ± 0.04 |
|  | r_spearman_ | 0.37 ± 0.04 | 0.46 ± 0.03 | 0.22 ± 0.04 | 0.33 ± 0.05 | 0.45 ± 0.03 | 0.46 ± 0.05 |
|  | RMSE | 0.39 ± 0.01 | 1.20 ± 0.05 | 0.19 ± 0.01 | 0.92 ± 0.04 | 0.26 ± 0.01 | 5.28 ± 0.22 |
|  | slope | 1.07 ± 0.05 | 1.03 ± 0.07 | 1.03 ± 0.04 | 1.02 ± 0.02 | 1.02 ± 0.05 | 1.06 ± 0.02 |
| Stack | r_pearson_ | 0.52 ± 0.02 | 0.52 ± 0.01 | 0.27 ± 0.02 | 0.39 ± 0.01 | 0.49 ± 0.03 | 0.58 ± 0.03 |
|  | r_spearman_ | 0.44 ± 0.03 | 0.48 ± 0.03 | 0.24 ± 0.02 | 0.38 ± 0.02 | 0.46 ± 0.02 | 0.51 ± 0.04 |
|  | RMSE | 0.35 ± 0.01 | 1.09 ± 0.06 | 0.16 ± 0.01 | 0.87 ± 0.02 | 0.25 ± 0.01 | 4.73 ± 0.19 |
|  | slope | 1.03 ± 0.05 | 1.06 ± 0.06 | 1.03 ± 0.04 | 1.02 ± 0.02 | 1.01 ± 0.05 | 1.03 ± 0.01 |

r_pearson_ – Pearson correlation; r_spearman_ – Spearman rank correlation; RMSE – root mean squared error. NEFA - non-esterified fatty acids; BHB - β-hydroxybutyrate.

**Table S6** Prediction metrics with standard errors, obtained from genomic BLUP (GBLUP), BayesB, elastic net (ENET), gradient boosting machine (GBM), and stacking ensemble (Stack) for liver function/hepatic damage for tenfold cross-validation.

| **Model** | **Metrics** | **AST** | **GGT** | **BILt** | **Albumin** | **ALP** | **PON** |
| --- | --- | --- | --- | --- | --- | --- | --- |
| Gblup | r_pearson_ | 0.47 ± 0.04 | 0.46 ± 0.06 | 0.32 ± 0.07 | 0.43 ± 0.03 | 0.49 ± 0.02 | 0.49 ± 0.05 |
|  | r_spearman_ | 0.41 ± 0.03 | 0.41 ± 0.04 | 0.23 ± 0.06 | 0.42 ± 0.03 | 0.47 ± 0.03 | 0.46 ± 0.06 |
|  | RMSE | 18.85 ± 1.99 | 7.51 ± 0.77 | 0.84 ± 0.10 | 1.43 ± 0.12 | 13.79 ± 1.12 | 15.25 ± 1.01 |
|  | slope | 0.92 ± 0.09 | 1.07 ± 0.08 | 1.07 ± 0.07 | 1.11 ± 0.13 | 1.04 ± 0.08 | 1.08 ± 0.05 |
| BayesB | r_pearson_ | 0.48 ± 0.04 | 0.47 ± 0.04 | 0.33 ± 0.07 | 0.45 ± 0.02 | 0.50 ± 0.03 | 0.50 ± 0.05 |
|  | r_spearman_ | 0.41 ± 0.03 | 0.42 ± 0.02 | 0.24 ± 0.06 | 0.43 ± 0.04 | 0.47 ± 0.04 | 0.46 ± 0.05 |
|  | RMSE | 18.84 ± 1.97 | 6.15 ± 0.53 | 0.77 ± 0.11 | 1.36 ± 0.15 | 13.53 ± 1.06 | 15.09 ± 0.9 |
|  | slope | 1.11 ± 0.09 | 0.94 ± 0.08 | 1.08 ± 0.08 | 1.15 ± 0.26 | 1.04 ± 0.08 | 1.09 ± 0.05 |
| ENET | r_pearson_ | 0.49 ± 0.05 | 0.48 ± 0.06 | 0.34 ± 0.06 | 0.45 ± 0.04 | 0.51 ± 0.04 | 0.54 ± 0.04 |
|  | r_spearman_ | 0.41 ± 0.04 | 0.45 ± 0.04 | 0.26 ± 0.04 | 0.43 ± 0.04 | 0.48 ± 0.03 | 0.46 ± 0.05 |
|  | RMSE | 18.7 ± 1.68 | 6.19 ± 0.69 | 0.79 ± 0.12 | 1.35 ± 0.17 | 13.16 ± 0.63 | 14.16 ± 0.89 |
|  | slope | 0.94 ± 0.08 | 1.07 ± 0.08 | 1.12 ± 0.03 | 1.18 ± 0.08 | 1.02 ± 0.05 | 0.98 ± 0.04 |
| GBM | r_pearson_ | 0.50 ± 0.03 | 0.50 ± 0.06 | 0.35 ± 0.07 | 0.46 ± 0.02 | 0.53 ± 0.02 | 0.58 ± 0.02 |
|  | r_spearman_ | 0.43 ± 0.06 | 0.47 ± 0.08 | 0.28 ± 0.06 | 0.43 ± 0.04 | 0.48 ± 0.04 | 0.54 ± 0.06 |
|  | RMSE | 17.24 ± 1.56 | 6.56 ± 0.59 | 0.73 ± 0.07 | 1.22 ± 0.05 | 12.82 ± 0.56 | 10.56 ± 0.36 |
|  | slope | 1.03 ± 0.02 | 0.98 ± 0.03 | 0.97 ± 0.08 | 1.05 ± 0.01 | 0.95 ± 0.03 | 1.02 ± 0.04 |
| Stack | r_pearson_ | 0.51 ± 0.04 | 0.52 ± 0.05 | 0.37 ± 0.05 | 0.47 ± 0.03 | 0.55 ± 0.02 | 0.60 ± 0.01 |
|  | r_spearman_ | 0.45 ± 0.06 | 0.48 ± 0.05 | 0.32 ± 0.06 | 0.44 ± 0.04 | 0.51 ± 0.03 | 0.58 ± 0.03 |
|  | RMSE | 16.78 ± 1.10 | 6.48 ± 0.60 | 0.74 ± 0.12 | 1.15 ± 0.05 | 11.47 ± 0.35 | 10.49 ± 0.05 |
|  | slope | 0.98 ± 0.01 | 1.06 ± 0.16 | 1.09 ± 0.09 | 1.05 ± 0.04 | 1.06 ± 0.06 | 1.03 ± 0.04 |

r_pearson_ – Pearson correlation; r_spearman_ – Spearman rank correlation; RMSE – root mean squared error. AST - aspartate aminotransferase; GGT - γ-glutamyl transferase; BILt - total bilirubin; ALP - alkaline phosphatase; PON – paraoxonase.

**Table S7** Prediction metrics with standard errors, obtained from genomic BLUP (GBLUP), BayesB, elastic net (ENET), gradient boosting machine (GBM), and stacking ensemble (Stack) for oxidative stress metabolites for tenfold cross-validation.

| **Model** | **Metrics** | **ROMt** | **AOPP** | **FRAP** | **SHp** |
| --- | --- | --- | --- | --- | --- |
| Gblup | r_pearson_ | 0.36 ± 0.03 | 0.55 ± 0.04 | 0.25 ± 0.04 | 0.52 ± 0.03 |
|  | r_spearman_ | 0.34 ± 0.03 | 0.44 ± 0.04 | 0.17 ± 0.04 | 0.44 ± 0.04 |
|  | RMSE | 3.14 ± 0.33 | 9.42 ± 0.81 | 37.41 ± 7.24 | 58.38 ± 7.57 |
|  | slope | 1.01 ± 0.03 | 1.01 ± 0.02 | 0.93 ± 0.07 | 1.08 ± 0.09 |
| BayesB | r_pearson_ | 0.38 ± 0.04 | 0.57 ± 0.04 | 0.27 ± 0.05 | 0.54 ± 0.02 |
|  | r_spearman_ | 0.35 ± 0.03 | 0.45 ± 0.04 | 0.18 ± 0.05 | 0.45 ± 0.03 |
|  | RMSE | 3.01 ± 0.19 | 8.93 ± 0.54 | 34.02 ± 5.58 | 52.34 ± 2.58 |
|  | slope | 1.07 ± 0.10 | 1.02 ± 0.02 | 0.93 ± 0.05 | 1.11 ± 0.09 |
| ENET | r_pearson_ | 0.41 ± 0.03 | 0.57 ± 0.03 | 0.29 ± 0.03 | 0.56 ± 0.02 |
|  | r_spearman_ | 0.36 ± 0.02 | 0.47 ± 0.04 | 0.20 ± 0.03 | 0.46 ± 0.03 |
|  | RMSE | 2.87 ± 0.23 | 8.80 ± 0.46 | 29.87 ± 2.54 | 44.69 ± 1.91 |
|  | slope | 0.95 ± 0.08 | 1.02 ± 0.02 | 0.98 ± 0.02 | 1.06 ± 0.01 |
| GBM | r_pearson_ | 0.42 ± 0.03 | 0.59 ± 0.03 | 0.30 ± 0.03 | 0.59 ± 0.01 |
|  | r_spearman_ | 0.37 ± 0.02 | 0.52 ± 0.02 | 0.22 ± 0.04 | 0.49 ± 0.03 |
|  | RMSE | 2.80 ± 0.15 | 7.41 ± 0.66 | 29.46 ± 2.26 | 44.59 ± 5.81 |
|  | slope | 1.03 ± 0.02 | 1.01 ± 0.01 | 1.01 ± 0.03 | 0.98 ± 0.02 |
| Stack | r_pearson_ | 0.45 ± 0.02 | 0.60 ± 0.02 | 0.35 ± 0.03 | 0.60 ± 0.01 |
|  | r_spearman_ | 0.39 ± 0.03 | 0.54 ± 0.02 | 0.26 ± 0.03 | 0.48 ± 0.02 |
|  | RMSE | 2.66 ± 0.15 | 7.21 ± 0.43 | 25.69 ± 3.26 | 42.13 ± 2.44 |
|  | slope | 1.03 ± 0.03 | 1.01 ± 0.01 | 1.01 ± 0.02 | 1.01 ± 0.01 |

r_pearson_ – Pearson correlation; r_spearman_ – Spearman rank correlation; RMSE – root mean squared error. ROMt - total reactive oxygen metabolites; AOPP - advanced oxidation protein products; FRAP - ferric reducing antioxidant power; SHp - thiolic groups.

**Table S8** Prediction metrics with standard errors, obtained from genomic BLUP (GBLUP), BayesB, elastic net (ENET), gradient boosting machine (GBM), and stacking ensemble (Stack) for inflammation/innate immunity for tenfold cross-validation.

| **Model** | **Metrics** | **Ceruloplasmin** | **PROT** | **Globulins** | **Haptoglobin** | **Myeloperoxidase** |
| --- | --- | --- | --- | --- | --- | --- |
| Gblup | r_pearson_ | 0.43 ± 0.06 | 0.35 ± 0.05 | 0.31 ± 0.03 | 0.17 ± 0.01 | 0.40 ± 0.07 |
|  | r_spearman_ | 0.39 ± 0.05 | 0.35 ± 0.02 | 0.32 ± 0.03 | 0.14 ± 0.07 | 0.38 ± 0.04 |
|  | RMSE | 0.68 ± 0.03 | 4.79 ± 0.50 | 5.52 ± 0.45 | 0.24 ± 0.02 | 75.09 ± 10.51 |
|  | slope | 0.97 ± 0.06 | 0.99 ± 0.01 | 1.03 ± 0.07 | 0.97 ± 0.01 | 0.98 ± 0.01 |
| BayesB | r_pearson_ | 0.47 ± 0.06 | 0.37 ± 0.05 | 0.33 ± 0.03 | 0.20 ± 0.04 | 0.42 ± 0.09 |
|  | r_spearman_ | 0.32 ± 0.07 | 0.36 ± 0.02 | 0.33 ± 0.03 | 0.17 ± 0.06 | 0.39 ± 0.03 |
|  | RMSE | 0.67 ± 0.03 | 4.74 ± 0.48 | 5.43 ± 0.50 | 0.23 ± 0.02 | 74.39 ± 8.78 |
|  | slope | 0.95 ± 0.05 | 0.99 ± 0.02 | 1.01 ± 0.04 | 1.05 ± 0.05 | 0.98 ± 0.06 |
| ENET | r_pearson_ | 0.51 ± 0.03 | 0.39 ± 0.05 | 0.34 ± 0.02 | 0.21 ± 0.03 | 0.43 ± 0.06 |
|  | r_spearman_ | 0.43 ± 0.09 | 0.37 ± 0.04 | 0.34 ± 0.02 | 0.19 ± 0.07 | 0.44 ± 0.03 |
|  | RMSE | 0.66 ± 0.05 | 4.60 ± 0.46 | 5.35 ± 0.46 | 0.24 ± 0.02 | 73.32 ± 9.59 |
|  | slope | 0.98 ± 0.02 | 1.01 ± 0.01 | 1.02 ± 0.02 | 1.01 ± 0.04 | 0.98 ± 0.01 |
| GBM | r_pearson_ | 0.53 ± 0.02 | 0.41 ± 0.06 | 0.37 ± 0.02 | 0.22 ± 0.02 | 0.45 ± 0.05 |
|  | r_spearman_ | 0.51 ± 0.04 | 0.39 ± 0.02 | 0.36 ± 0.02 | 0.20 ± 0.06 | 0.45 ± 0.03 |
|  | RMSE | 0.65 ± 0.05 | 4.58 ± 0.44 | 5.27 ± 0.46 | 0.24 ± 0.02 | 72.55 ± 9.22 |
|  | slope | 1.01 ± 0.01 | 1.01 ± 0.01 | 1.03 ± 0.03 | 0.97 ± 0.03 | 1.02 ± 0.01 |
| Stack | r_pearson_ | 0.55 ± 0.03 | 0.43 ± 0.05 | 0.38 ± 0.02 | 0.24 ± 0.02 | 0.47 ± 0.03 |
|  | r_spearman_ | 0.52 ± 0.04 | 0.40 ± 0.03 | 0.37 ± 0.02 | 0.23 ± 0.02 | 0.46 ± 0.03 |
|  | RMSE | 0.64 ± 0.04 | 4.52 ± 0.43 | 5.20 ± 0.45 | 0.23 ± 0.02 | 71.58 ± 9.25 |
|  | slope | 1.03 ± 0.05 | 0.99 ± 0.01 | 1.03 ± 0.03 | 1.07 ± 0.08 | 1.02 ± 0.01 |

r_pearson_ – Pearson correlation; r_spearman_ – Spearman rank correlation; RMSE – root mean squared error. PROTt – total proteins

**Table S9** Prediction metrics with standard errors, obtained from genomic BLUP (GBLUP), BayesB, elastic net (ENET), gradient booSting machine (GBM), and stacking ensemble (Stack) for blood minerals for tenfold cross-validation.

| **Model** | **Metrics** | **Calcium** | **Phosphorus** | **Magnesium** | **Sodium** | **Potassium** | **Chlorine** | **Zinc** |
| --- | --- | --- | --- | --- | --- | --- | --- | --- |
| Gblup | r_pearson_ | 0.38 ± 0.02 | 0.37 ± 0.03 | 0.38 ± 0.05 | 0.37 ± 0.05 | 0.39 ± 0.02 | 0.51 ± 0.05 | 0.41 ± 0.05 |
|  | r_spearman_ | 0.36 ± 0.02 | 0.37 ± 0.03 | 0.36 ± 0.04 | 0.34 ± 0.04 | 0.38 ± 0.01 | 0.43 ± 0.05 | 0.34 ± 0.05 |
|  | RMSE | 0.06 ± 0.009 | 0.17 ± 0.01 | 0.06 ± 0.004 | 2.23 ± 0.18 | 0.20 ± 0.01 | 2.37 ± 0.13 | 2.77 ± 0.15 |
|  | slope | 0.98 ± 0.03 | 0.99 ± 0.02 | 0.97 ± 0.02 | 0.99 ± 0.01 | 0.98 ± 0.02 | 1.02 ± 0.02 | 1.03 ± 0.05 |
| BayesB | r_pearson_ | 0.39 ± 0.02 | 0.41 ± 0.01 | 0.39 ± 0.03 | 0.38 ± 0.04 | 0.40 ± 0.02 | 0.53 ± 0.05 | 0.45 ± 0.07 |
|  | r_spearman_ | 0.37 ± 0.02 | 0.40 ± 0.02 | 0.37 ± 0.03 | 0.36 ± 0.04 | 0.38 ± 0.02 | 0.44 ± 0.03 | 0.36 ± 0.03 |
|  | RMSE | 0.06 ± 0.003 | 0.14 ± 0.01 | 0.05 ± 0.004 | 2.15 ± 0.13 | 0.19 ± 0.01 | 2.33 ± 0.13 | 2.70 ± 0.13 |
|  | slope | 1.01 ± 0.01 | 1.04 ± 0.02 | 1.03 ± 0.03 | 1.01 ± 0.02 | 1.01 ± 0.01 | 0.95 ± 0.01 | 1.02 ± 0.04 |
| ENET | r_pearson_ | 0.40 ± 0.03 | 0.41 ± 0.01 | 0.40 ± 0.04 | 0.40 ± 0.02 | 0.40 ± 0.04 | 0.54 ± 0.05 | 0.49 ± 0.04 |
|  | r_spearman_ | 0.39 ± 0.03 | 0.44 ± 0.03 | 0.42 ± 0.05 | 0.40 ± 0.03 | 0.40 ± 0.03 | 0.47 ± 0.04 | 0.43 ± 0.03 |
|  | RMSE | 0.05 ± 0.01 | 0.13 ± 0.01 | 0.05 ± 0.01 | 2.02 ± 0.14 | 0.18 ± 0.04 | 2.11 ± 0.58 | 2.57 ± 0.29 |
|  | slope | 1.03 ± 0.02 | 0.99 ± 0.01 | 1.08 ± 0.05 | 1.04 ± 0.01 | 1.02 ± 0.01 | 1.05 ± 0.01 | 0.98 ± 0.02 |
| GBM | r_pearson_ | 0.41 ± 0.01 | 0.42 ± 0.01 | 0.41 ± 0.03 | 0.44 ± 0.03 | 0.41 ± 0.01 | 0.55 ± 0.04 | 0.51 ± 0.03 |
|  | r_spearman_ | 0.41 ± 0.03 | 0.42 ± 0.04 | 0.40 ± 0.07 | 0.42 ± 0.03 | 0.40 ± 0.03 | 0.42 ± 0.04 | 0.42 ± 0.04 |
|  | RMSE | 0.05 ± 0.003 | 0.12 ± 0.01 | 0.04 ± 0.03 | 1.72 ± 0.31 | 0.16 ± 0.02 | 2.15 ± 0.31 | 2.49 ± 0.16 |
|  | slope | 0.98 ± 0.01 | 1.02 ± 0.02 | 1.02 ± 0.02 | 1.03 ± 0.01 | 1.06 ± 0.02 | 0.98 ± 0.01 | 1.03 ± 0.05 |
| Stack | r_pearson_ | 0.42 ± 0.02 | 0.44 ± 0.01 | 0.41 ± 0.03 | 0.45 ± 0.01 | 0.42 ± 0.02 | 0.57 ± 0.02 | 0.53 ± 0.03 |
|  | r_spearman_ | 0.44 ± 0.04 | 0.45 ± 0.03 | 0.45 ± 0.06 | 0.42 ± 0.03 | 0.41 ± 0.03 | 0.52 ± 0.03 | 0.46 ± 0.04 |
|  | RMSE | 0.04 ± 0.03 | 0.11 ± 0.01 | 0.05 ± 0.01 | 1.70 ± 0.13 | 0.16 ± 0.02 | 1.92 ± 0.54 | 2.47 ± 0.11 |
|  | slope | 1.01 ± 0.02 | 1.02 ± 0.02 | 1.05 ± 0.03 | 1.05 ± 0.01 | 1.04 ± 0.02 | 1.05 ± 0.01 | 0.99 ± 0.02 |

r_pearson_ – Pearson correlation; r_spearman_ – Spearman rank correlation; RMSE – root mean squared error

**Table S10** Prediction metrics with standard errors, obtained from genomic BLUP (GBLUP), BayesB, elastic net (ENET), gradient boosting machine (GBM), and stacking ensemble (Stack) for energy-related metabolites for batch-out cross-validation.

| **Model** | **Metrics** | **Glucose** | **Cholesterol** | **NEFA** | **BHB** | **Urea** | **Creatinine** |
| --- | --- | --- | --- | --- | --- | --- | --- |
| Gblup | r_pearson_ | 0.34 ± 0.09 | 0.41 ± 0.15 | 0.20 ± 0.14 | 0.30 ± 0.09 | 0.40 ± 0.18 | 0.42 ± 0.06 |
|  | r_spearman_ | 0.31 ± 0.09 | 0.39 ± 0.15 | 0.20 ± 0.13 | 0.40 ± 0.08 | 0.39 ± 0.15 | 0.41 ± 0.06 |
|  | RMSE | 0.98 ± 0.04 | 1.16 ± 0.10 | 0.27 ± 0.99 | 0.44 ± 0.11 | 0.25 ± 0.03 | 0.35 ± 0.08 |
|  | slope | 0.90 ± 0.32 | 1.09 ± 0.46 | 1.05 ± 0.58 | 0.91 ± 0.40 | 0.97 ± 0.09 | 1.01 ± 0.13 |
| BayesB | r_pearson_ | 0.37 ± 0.09 | 0.41 ± 0.14 | 0.21 ± 0.19 | 0.31 ± 0.09 | 0.37 ± 0.05 | 0.48 ± 0.15 |
|  | r_spearman_ | 0.36 ± 0.10 | 0.59 ± 0.14 | 0.24 ± 0.17 | 0.29 ± 0.10 | 0.35 ± 0.06 | 0.45 ± 0.13 |
|  | RMSE | 0.48 ± 0.11 | 1.21 ± 0.10 | 0.25 ± 0.03 | 0.98 ± 0.04 | 0.39 ± 0.09 | 5.73 ± 0.98 |
|  | slope | 0.96 ± 0.46 | 1.07 ± 0.44 | 0.94 ± 0.56 | 0.89 ± 0.14 | 0.99 ± 0.10 | 1.08 ± 0.58 |
| ENET | r_pearson_ | 0.37 ± 0.04 | 0.43 ± 0.04 | 0.20 ± 0.05 | 0.29 ± 0.08 | 0.39 ± 0.06 | 0.42 ± 0.06 |
|  | r_spearman_ | 0.35 ± 0.08 | 0.42 ± 0.08 | 0.18 ± 0.03 | 0.28 ± 0.07 | 0.36 ± 0.04 | 0.41 ± 0.06 |
|  | RMSE | 0.51 ± 0.03 | 1.28 ± 0.11 | 0.25 ± 0.02 | 0.93 ± 0.08 | 0.33 ± 0.02 | 6.38 ± 0.28 |
|  | slope | 0.97 ± 0.07 | 0.97 ± 0.09 | 1.07 ± 0.04 | 0.9 ± 0.05 | 1.06 ± 0.08 | 1.10 ± 0.04 |
| GBM | r_pearson_ | 0.41 ± 0.04 | 0.46 ± 0.02 | 0.23 ± 0.03 | 0.37 ± 0.94 | 0.42 ± 0.05 | 0.48 ± 0.05 |
|  | r_spearman_ | 0.41 ± 0.05 | 0.45 ± 0.04 | 0.20 ± 0.04 | 0.33 ± 0.11 | 0.41 ± 0.04 | 0.49 ± 0.06 |
|  | RMSE | 0.48 ± 0.02 | 1.25 ± 0.06 | 0.22 ± 0.01 | 0.92 ± 0.22 | 0.31 ± 0.01 | 6.04 ± 0.24 |
|  | slope | 0.96 ± 0.05 | 0.96 ± 0.08 | 1.03 ± 0.05 | 1.02 ± 0.09 | 1.05 ± 0.06 | 1.01 ± 0.03 |
| Stack | r_pearson_ | 0.44 ± 0.03 | 0.49 ± 0.02 | 0.25 ± 0.02 | 0.36 ± 0.01 | 0.45 ± 0.04 | 0.52 ± 0.04 |
|  | r_spearman_ | 0.40 ± 0.04 | 0.39 ± 0.04 | 0.21 ± 0.03 | 0.32 ± 0.02 | 0.44 ± 0.02 | 0.51 ± 0.05 |
|  | RMSE | 0.46 ± 0.04 | 1.15 ± 0.08 | 0.20 ± 0.01 | 0.19 ± 0.02 | 0.29 ± 0.01 | 5.84 ± 0.21 |
|  | slope | 0.98 ± 0.06 | 0.95 ± 0.07 | 0.99 ± 0.05 | 0.95 ± 0.03 | 1.50 ± 0.06 | 1.06 ± 0.02 |

r_pearson_ – Pearson correlation; r_spearman_ – Spearman rank correlation; RMSE – root mean squared error. NEFA - non-esterified fatty acids; BHB - β-hydroxybutyrate

**Table S11** Prediction metrics with standard errors, obtained from genomic BLUP (GBLUP), BayesB, elastic net (ENET), gradient boosting machine (GBM), and stacking ensemble (Stack) for liver function/hepatic damage for batch-out cross-validation.

| **Model** | **Metrics** | **AST** | **GGT** | **BILt** | **Albumin** | **ALP** | **PON** |
| --- | --- | --- | --- | --- | --- | --- | --- |
| Gblup | r_pearson_ | 0.37 ± 0.05 | 0.36 ± 0.08 | 0.26 ± 0.09 | 0.35 ± 0.03 | 0.39 ± 0.03 | 0.40 ± 0.07 |
|  | r_spearman_ | 0.33 ± 0.04 | 0.33 ± 0.06 | 0.18 ± 0.08 | 0.33 ± 0.04 | 0.37 ± 0.04 | 0.37 ± 0.08 |
|  | RMSE | 19.25 ± 2.33 | 8.12 ± 0.91 | 1.11 ± 0.12 | 1.55 ± 0.14 | 14.48 ± 1.32 | 16.01 ± 1.17 |
|  | slope | 0.93 ± 0.11 | 1.07 ± 0.10 | 0.98 ± 0.09 | 1.11 ± 0.15 | 1.04 ± 0.13 | 1.08 ± 0.07 |
| BayesB | r_pearson_ | 0.39 ± 0.05 | 0.38 ± 0.05 | 0.26 ± 0.10 | 0.36 ± 0.03 | 0.40 ± 0.04 | 0.39 ± 0.07 |
|  | r_spearman_ | 0.33 ± 0.04 | 0.34 ± 0.03 | 0.19 ± 0.08 | 0.34 ± 0.05 | 0.38 ± 0.05 | 0.37 ± 0.07 |
|  | RMSE | 19.22 ± 2.23 | 8.02 ± 0.62 | 0.99 ± 0.13 | 1.43 ± 0.18 | 14.22 ± 1.23 | 15.85 ± 1.12 |
|  | slope | 1.11 ± 0.11 | 0.94 ± 0.09 | 0.92 ± 0.11 | 1.15 ± 0.30 | 1.04 ± 0.15 | 1.09 ± 0.07 |
| ENET | r_pearson_ | 0.38 ± 0.07 | 0.38 ± 0.10 | 0.28 ± 0.09 | 0.35 ± 0.05 | 0.40 ± 0.05 | 0.43 ± 0.06 |
|  | r_spearman_ | 0.33 ± 0.05 | 0.36 ± 0.05 | 0.21 ± 0.05 | 0.34 ± 0.05 | 0.38 ± 0.04 | 0.42 ± 0.06 |
|  | RMSE | 19.22 ± 2 | 7.95 ± 0.81 | 0.85 ± 0.15 | 1.41 ± 0.22 | 13.88 ± 0.74 | 14.89 ± 1.04 |
|  | slope | 0.94 ± 0.09 | 1.07 ± 0.10 | 0.97 ± 0.04 | 1.18 ± 0.15 | 1.02 ± 0.07 | 0.98 ± 0.05 |
| GBM | r_pearson_ | 0.40 ± 0.04 | 0.40 ± 0.08 | 0.28 ± 0.08 | 0.37 ± 0.02 | 0.42 ± 0.03 | 0.46 ± 0.03 |
|  | r_spearman_ | 0.35 ± 0.08 | 0.38 ± 0.09 | 0.23 ± 0.07 | 0.34 ± 0.05 | 0.38 ± 0.06 | 0.44 ± 0.08 |
|  | RMSE | 18.95 ± 1.79 | 7.82 ± 0.68 | 0.83 ± 0.09 | 1.28 ± 0.06 | 13.49 ± 0.65 | 11.70 ± 0.43 |
|  | slope | 1.03 ± 0.03 | 0.98 ± 0.04 | 0.95 ± 0.09 | 1.05 ± 0.02 | 0.95 ± 0.04 | 1.02 ± 0.05 |
| Stack | r_pearson_ | 0.41 ± 0.06 | 0.41 ± 0.06 | 0.30 ± 0.07 | 0.37 ± 0.04 | 0.44 ± 0.03 | 0.47 ± 0.02 |
|  | r_spearman_ | 0.36 ± 0.08 | 0.38 ± 0.07 | 0.26 ± 0.08 | 0.36 ± 0.05 | 0.41 ± 0.04 | 0.46 ± 0.05 |
|  | RMSE | 18.88 ± 1.28 | 7.55 ± 0.71 | 0.81 ± 0.14 | 1.21 ± 0.06 | 12.85 ± 0.41 | 11.55 ± 0.07 |
|  | slope | 0.98 ± 0.02 | 1.06 ± 0.09 | 0.98 ± 0.08 | 1.05 ± 0.05 | 1.06 ± 0.08 | 1.03 ± 0.06 |

r_pearson_ – Pearson correlation; r_spearman_ – Spearman rank correlation; RMSE – root mean squared error. AST - aspartate aminotransferase; GGT - γ-glutamyl transferase; BILt - total bilirubin; ALP - alkaline phosphatase and PON – Paraoxonase.

**Table S12** Prediction metrics with standard errors, obtained from genomic BLUP (GBLUP), BayesB, elastic net (ENET), gradient boosting machine (GBM), and stacking ensemble (Stack) for oxidative stress metabolites for batch-out cross-validation.

| **Model** | **Metrics** | **ROMt** | **AOPP** | **FRAP** | **SHp** |
| --- | --- | --- | --- | --- | --- |
| Gblup | r_pearson_ | 0.31 ± 0.04 | 0.45 ± 0.05 | 0.21 ± 0.06 | 0.46 ± 0.04 |
|  | r_spearman_ | 0.30 ± 0.04 | 0.36 ± 0.06 | 0.14 ± 0.05 | 0.39 ± 0.05 |
|  | RMSE | 3.62 ± 0.41 | 9.85 ± 1.07 | 40.53 ± 8.39 | 60.62 ± 8.43 |
|  | slope | 0.94 ± 0.04 | 1.24 ± 0.03 | 1.07 ± 0.09 | 1.03 ± 0.11 |
| BayesB | r_pearson_ | 0.33 ± 0.05 | 0.46 ± 0.06 | 0.23 ± 0.06 | 0.40 ± 0.03 |
|  | r_spearman_ | 0.30 ± 0.04 | 0.36 ± 0.06 | 0.15 ± 0.07 | 0.39 ± 0.04 |
|  | RMSE | 3.46 ± 0.24 | 9.33 ± 0.71 | 36.74 ± 6.45 | 63.02 ± 2.87 |
|  | slope | 0.93 ± 0.14 | 1.25 ± 0.03 | 0.95 ± 0.07 | 0.97 ± 0.11 |
| ENET | r_pearson_ | 0.36 ± 0.04 | 0.47 ± 0.05 | 0.25 ± 0.05 | 0.50 ± 0.03 |
|  | r_spearman_ | 0.31 ± 0.04 | 0.38 ± 0.06 | 0.17 ± 0.04 | 0.49 ± 0.04 |
|  | RMSE | 3.27 ± 0.29 | 9.25 ± 0.60 | 31.96 ± 2.89 | 58.76 ± 2.11 |
|  | slope | 1.03 ± 0.10 | 1.27 ± 0.03 | 0.97 ± 0.03 | 1.02 ± 0.01 |
| GBM | r_pearson_ | 0.36 ± 0.04 | 0.48 ± 0.05 | 0.25 ± 0.05 | 0.52 ± 0.02 |
|  | r_spearman_ | 0.33 ± 0.04 | 0.43 ± 0.03 | 0.19 ± 0.05 | 0.50 ± 0.04 |
|  | RMSE | 3.21 ± 0.19 | 8.75 ± 0.87 | 31.7 ± 2.60 | 57.68 ± 6.42 |
|  | slope | 0.89 ± 0.03 | 1.24 ± 0.01 | 0.96 ± 0.04 | 1.01 ± 0.03 |
| Stack | r_pearson_ | 0.39 ± 0.03 | 0.49 ± 0.03 | 0.30 ± 0.04 | 0.56 ± 0.02 |
|  | r_spearman_ | 0.34 ± 0.05 | 0.44 ± 0.03 | 0.22 ± 0.04 | 0.55 ± 0.03 |
|  | RMSE | 3.06 ± 0.22 | 8.40 ± 0.56 | 27.77 ± 3.78 | 55.77 ± 2.71 |
|  | slope | 0.98 ± 0.05 | 1.02 ± 0.07 | 0.99 ± 0.03 | 1.03 ± 0.01 |

r_pearson_ – Pearson correlation; r_spearman_ – Spearman rank correlation; RMSE – root mean squared error. ROMt - total reactive oxygen metabolites; AOPP - advanced oxidation protein products; FRAP - ferric reducing antioxidant power; SHp - thiolic groups.

**Table S13** Prediction metrics with standard errors, obtained from genomic BLUP (GBLUP), BayesB, elastic net (ENET), gradient boosting machine (GBM), and stacking ensemble (Stack) for inflammation/innate immunity for tenfold cross-validation.

| **Model** | **Fit parameter** | **Ceruloplasmin** | **PROTt** | **Globulins** | **Haptoglobin** | **Myeloperoxidase** |
| --- | --- | --- | --- | --- | --- | --- |
| Gblup | r | 0.41 ± 0.06 | 0.31 ± 0.12 | 0.29 ± 0.12 | 0.16 ± 0.07 | 0.35 ± 0.15 |
|  | rsp | 0.40 ± 0.07 | 0.31 ± 0.11 | 0.28 ± 0.11 | 0.15 ± 0.12 | 0.34 ± 0.12 |
|  | RMSE | 0.72 ± 0.04 | 5.08 ± 1.07 | 5.95 ± 0.94 | 0.33 ± 0.05 | 75.49 ± 13.14 |
|  | slope | 1.01 ± 0.12 | 0.90 ± 0.52 | 0.91 ± 0.56 | 0.92 ± 0.82 | 0.97 ± 0.94 |
| BayesB | r | 0.36 ± 0.09 | 0.32 ± 0.07 | 0.29 ± 0.04 | 0.17 ± 0.06 | 0.35 ± 0.11 |
|  | rsp | 0.24 ± 0.10 | 0.31 ± 0.03 | 0.29 ± 0.04 | 0.15 ± 0.07 | 0.34 ± 0.04 |
|  | RMSE | 0.74 ± 0.04 | 4.98 ± 0.57 | 5.70 ± 0.59 | 0.24 ± 0.03 | 76.11 ± 10.37 |
|  | slope | 1.06 ± 0.07 | 1.11 ± 0.03 | 0.88 ± 0.05 | 0.89 ± 0.06 | 1.13 ± 0.07 |
| EN | r | 0.48 ± 0.04 | 0.33 ± 0.06 | 0.31 ± 0.02 | 0.19 ± 0.03 | 0.36 ± 0.07 |
|  | rsp | 0.46 ± 0.11 | 0.32 ± 0.05 | 0.29 ± 0.03 | 0.18 ± 0.09 | 0.35 ± 0.04 |
|  | RMSE | 0.57 ± 0.06 | 4.30 ± 0.54 | 5.75 ± 0.54 | 0.38 ± 0.03 | 74.65 ± 11.03 |
|  | slope | 0.89 ± 0.03 | 0.90 ± 0.02 | 0.93 ± 0.03 | 0.92 ± 0.05 | 1.04 ± 0.01 |
| GBM | r | 0.50 ± 0.03 | 0.35 ± 0.07 | 0.34 ± 0.02 | 0.21 ± 0.03 | 0.39 ± 0.06 |
|  | rsp | 0.48 ± 0.05 | 0.34 ± 0.03 | 0.33 ± 0.03 | 0.20 ± 0.08 | 0.37 ± 0.04 |
|  | RMSE | 0.55 ± 0.06 | 4.12 ± 0.52 | 5.45 ± 0.53 | 0.33 ± 0.03 | 73.31 ± 10.60 |
|  | slope | 0.91 ± 0.01 | 0.91 ± 0.01 | 1.01 ± 0.04 | 1.09 ± 0.04 | 0.95 ± 0.02 |
| Stack | r | 0.58 ± 0.03 | 0.38 ± 0.07 | 0.38 ± 0.03 | 0.22 ± 0.03 | 0.41 ± 0.03 |
|  | rsp | 0.56 ± 0.05 | 0.37 ± 0.04 | 0.37 ± 0.03 | 0.21 ± 0.03 | 0.40 ± 0.04 |
|  | RMSE | 0.59 ± 0.05 | 4.02 ± 0.49 | 5.11 ± 0.53 | 0.30 ± 0.03 | 73.25 ± 10.64 |
|  | slope | 0.95 ± 0.06 | 0.95 ± 0.01 | 0.97 ± 0.04 | 0.95 ± 0.09 | 0.98 ± 0.02 |

r_pearson_ – Pearson correlation; r_spearman_ – Spearman rank correlation; RMSE – root mean squared error. PROTt – total proteins

**Table S14** Prediction metrics with standard errors, obtained from genomic BLUP (GBLUP), BayesB, elastic net (ENET), gradient boosting machine (GBM), and stacking ensemble (Stack) for blood minerals for batch-out cross-validation.

| **Model** | **Metrics** | **Calcium** | **Phosphorus** | **Magnesium** | **Sodium** | **Potassium** | **Chlorine** | **Zinc** |
| --- | --- | --- | --- | --- | --- | --- | --- | --- |
| Gblup | r_pearson_ | 0.32 ± 0.04 | 0.33 ± 0.03 | 0.33 ± 0.07 | 0.32 ± 0.07 | 0.33 ± 0.04 | 0.44 ± 0.09 | 0.39 ± 0.12 |
|  | r_spearman_ | 0.30 ± 0.04 | 0.32 ± 0.04 | 0.30 ± 0.06 | 0.29 ± 0.07 | 0.32 ± 0.04 | 0.42 ± 0.14 | 0.38 ± 0.11 |
|  | RMSE | 0.07 ± 0.01 | 0.19 ± 0.02 | 0.07 ± 0.01 | 2.62 ± 0.26 | 0.24 ± 0.03 | 3.62 ± 0.27 | 3.86 ± 0.22 |
|  | slope | 1.05 ± 0.04 | 1.02 ± 0.03 | 1.05 ± 0.03 | 1.05 ± 0.01 | 1.07 ± 0.03 | 1.06 ± 0.04 | 0.98 ± 0.06 |
| BayesB | r_pearson_ | 0.33 ± 0.04 | 0.36 ± 0.01 | 0.33 ± 0.05 | 0.32 ± 0.06 | 0.34 ± 0.03 | 0.43 ± 0.12 | 0.37 ± 0.10 |
|  | r_spearman_ | 0.31 ± 0.03 | 0.35 ± 0.02 | 0.31 ± 0.05 | 0.31 ± 0.06 | 0.32 ± 0.04 | 0.40 ± 0.13 | 0.24 ± 0.09 |
|  | RMSE | 0.07 ± 0.01 | 0.16 ± 0.01 | 0.06 ± 0.01 | 2.51 ± 0.19 | 0.23 ± 0.03 | 3.69 ± 0.26 | 4.27 ± 0.19 |
|  | slope | 1.09 ± 0.02 | 1.07 ± 0.03 | 1.10 ± 0.04 | 1.08 ± 0.03 | 1.04 ± 0.02 | 0.99 ± 0.02 | 0.94 ± 0.05 |
| ENET | r_pearson_ | 0.34 ± 0.08 | 0.36 ± 0.02 | 0.35 ± 0.06 | 0.34 ± 0.03 | 0.33 ± 0.08 | 0.46 ± 0.09 | 0.42 ± 0.07 |
|  | r_spearman_ | 0.33 ± 0.09 | 0.38 ± 0.07 | 0.36 ± 0.14 | 0.34 ± 0.07 | 0.33 ± 0.15 | 0.44 ± 0.14 | 0.40 ± 0.08 |
|  | RMSE | 0.06 ± 0.03 | 0.15 ± 0.01 | 0.06 ± 0.01 | 2.38 ± 0.20 | 0.21 ± 0.08 | 3.21 ± 1.21 | 3.65 ± 0.42 |
|  | slope | 1.11 ± 0.03 | 1.04 ± 0.02 | 1.15 ± 0.06 | 1.11 ± 0.01 | 1.03 ± 0.01 | 1.09 ± 0.02 | 0.98 ± 0.03 |
| GBM | r_pearson_ | 0.35 ± 0.02 | 0.37 ± 0.02 | 0.35 ± 0.19 | 0.37 ± 0.05 | 0.35 ± 0.03 | 0.48 ± 0.09 | 0.45 ± 0.08 |
|  | r_spearman_ | 0.35 ± 0.05 | 0.37 ± 0.06 | 0.34 ± 0.21 | 0.36 ± 0.07 | 0.33 ± 0.07 | 0.47 ± 0.13 | 0.43 ± 0.10 |
|  | RMSE | 0.06 ± 0.01 | 0.14 ± 0.02 | 0.05 ± 0.04 | 2.00 ± 0.44 | 0.19 ± 0.04 | 3.20 ± 0.64 | 3.52 ± 0.23 |
|  | slope | 1.05 ± 0.02 | 1.07 ± 0.08 | 1.09 ± 0.03 | 1.08 ± 0.07 | 1.08 ± 0.02 | 1.02 ± 0.01 | 0.99 ± 0.06 |
| Stack | r_pearson_ | 0.36 ± 0.06 | 0.39 ± 0.01 | 0.35 ± 0.06 | 0.38 ± 0.02 | 0.35 ± 0.04 | 0.50 ± 0.07 | 0.48 ± 0.08 |
|  | r_spearman_ | 0.37 ± 0.06 | 0.39 ± 0.04 | 0.38 ± 0.11 | 0.36 ± 0.06 | 0.35 ± 0.07 | 0.49 ± 0.11 | 0.46 ± 0.09 |
|  | RMSE | 0.05 ± 0.03 | 0.13 ± 0.01 | 0.06 ± 0.01 | 1.98 ± 0.19 | 0.19 ± 0.04 | 2.85 ± 1.11 | 3.28 ± 0.15 |
|  | slope | 1.09 ± 0.02 | 1.06 ± 0.09 | 1.11 ± 0.04 | 1.08 ± 0.02 | 0.97 ± 0.03 | 1.09 ± 0.02 | 1.01 ± 0.02 |

r_pearson_ – Pearson correlation; r_spearman_ – Spearman rank correlation; RMSE – root mean squared error

**Table S15** Prediction fit parameters, including standard errors, considering the top 1500 SNP markers ranked by a GBM, obtained from genomic BLUP (GBLUP), Bayesian B (BayesB), elastic net (ENET), gradient boosting machine (GBM), and stacking ensemble (Stack) for Energy-related metabolites.

| **Model** | **Fit parameter** | **Glucose** | **Cholesterol** | **NEFA** | **BHB** | **Urea** | **Creatinine** |
| --- | --- | --- | --- | --- | --- | --- | --- |
| GBLUP | r_pearson_ | 0.48 ± 0.07 | 0.46 ± 0.07 | 0.29 ± 0.11 | 0.37 ± 0.09 | 0.47 ± 0.03 | 0.56 ± 0.06 |
|  | r_spearman_ | 0.46 ± 0.08 | 0.40 ± 0.07 | 0.27 ± 0.10 | 0.30 ± 0.06 | 0.46 ± 0.05 | 0.53 ± 0.08 |
|  | RMSE | 0.37 ± 0.03 | 1.12 ± 0.11 | 0.13 ± 0.04 | 0.18 ± 0.03 | 0.89 ± 0.04 | 5.62 ± 0.56 |
|  | slope | 0.98 ± 0.11 | 1.00 ± 0.09 | 0.93 ± 0.08 | 0.96 ± 0.10 | 0.99 ± 0.07 | 1.02 ± 0.05 |
| ENET | r_pearson_ | 0.49 ± 0.06 | 0.47 ± 0.07 | 0.3 ± 0.09 | 0.38 ± 0.09 | 0.48 ± 0.03 | 0.57 ± 0.04 |
|  | r_spearman_ | 0.47 ± 0.08 | 0.45 ± 0.07 | 0.28 ± 0.09 | 0.33 ± 0.06 | 0.48 ± 0.03 | 0.54 ± 0.05 |
|  | RMSE | 0.35 ± 0.04 | 1.07 ± 0.11 | 0.13 ± 0.04 | 0.18 ± 0.03 | 0.87 ± 0.05 | 5.47 ± 0.05 |
|  | slope | 0.985 ± 0.07 | 1.073 ± 0.08 | 1.2 ± 2.51 | 1.04 ± 0.04 | 1.08 ± 0.11 | 0.99 ± 0.08 |
| GBM | r_pearson_ | 0.49 ± 0.09 | 0.46 ± 0.07 | 0.3 ± 0.09 | 0.39 ± 0.09 | 0.46 ± 0.08 | 0.53 ± 0.08 |
|  | r_spearman_ | 0.48 ± 0.09 | 0.45 ± 0.07 | 0.29 ± 0.09 | 0.34 ± 0.06 | 0.46 ± 0.09 | 0.53 ± 0.08 |
|  | RMSE | 0.35 ± 0.05 | 1.11 ± 0.06 | 0.12 ± 0.04 | 0.18 ± 0.03 | 0.91 ± 0.12 | 5.85 ± 0.68 |
|  | slope | 1.10 ± 0.08 | 1.01 ± 0.06 | 1.07 ± 0.81 | 0.97 ± 0.06 | 1.02 ± 0.23 | 1.05 ± 0.10 |
| Stack | r_pearson_ | 0.52 ± 0.06 | 0.48 ± 0.07 | 0.33 ± 0.08 | 0.40 ± 0.08 | 0.50 ± 0.03 | 0.56 ± 0.03 |
|  | r_spearman_ | 0.52 ± 0.07 | 0.46 ± 0.08 | 0.32 ± 0.09 | 0.39 ± 0.06 | 0.50 ± 0.04 | 0.56 ± 0.04 |
|  | RMSE | 0.34 ± 0.03 | 1.01 ± 0.11 | 0.11 ± 0.03 | 0.16 ± 0.02 | 0.84 ± 0.04 | 5.45 ± 0.45 |
|  | slope | 0.99 ± 0.06 | 0.94 ± 0.05 | 1.02 ± 0.08 | 0.98 ± 0.03 | 0.94 ± 0.06 | 1.02 ± 0.07 |

r_pearson_ – Pearson correlation; r_spearman_ – Spearman rank correlation; RMSE – root mean square. NEFA - non-esterified fatty acids; BHB - β-hydroxybutyrate

**Table S16** Prediction fit parameters, including standard errors, considering the top 1500 SNP markers ranked by a GBM, obtained from genomic BLUP (GBLUP), Bayesian B (BayesB), elastic net (ENET), gradient boosting machine (GBM) and stacking ensemble (Stack) for Liver function/hepatic damage.

| **Model** | **Fit parameter** | **AST** | **GGT** | **BILt** | **Albumin** | **ALP** | **PON** |
| --- | --- | --- | --- | --- | --- | --- | --- |
| GBLUP | r_pearson_ | 0.48 ± 0.02 | 0.47 ± 0.09 | 0.35 ± 0.07 | 0.44 ± 0.07 | 0.51 ± 0.01 | 0.50 ± 0.02 |
|  | r_spearman_ | 0.45 ± 0.03 | 0.47 ± 0.04 | 0.31 ± 0.03 | 0.47 ± 0.03 | 0.49 ± 0.01 | 0.48 ± 0.03 |
|  | RMSE | 17.47 ± 2.51 | 9.73 ± 3.28 | 1.03 ± 0.25 | 1.67 ± 0.35 | 12.25 ± 1.2 | 13.27 ± 0.7 |
|  | slope | 0.99 ± 0.09 | 0.97 ± 0.05 | 0.91 ± 0.04 | 1.06 ± 0.04 | 1.01 ± 0.09 | 1.03 ± 0.08 |
| ENET | r_pearson_ | 0.47 ± 0.04 | 0.46 ± 0.08 | 0.34 ± 0.12 | 0.48 ± 0.04 | 0.52 ± 0.02 | 0.50 ± 0.03 |
|  | r_spearman_ | 0.45 ± 0.05 | 0.43 ± 0.04 | 0.30 ± 0.05 | 0.51 ± 0.05 | 0.50 ± 0.02 | 0.48 ± 0.03 |
|  | RMSE | 19.95 ± 2.06 | 9.33 ± 3.20 | 1.13 ± 0.37 | 2.02 ± 0.13 | 13.65 ± 0.83 | 13.8 ± 0.85 |
|  | slope | 0.88 ± 0.08 | 1.06 ± 0.06 | 1.06 ± 0.07 | 0.97 ± 0.09 | 0.98 ± 0.78 | 1.02 ± 0.08 |
| GBM | r_pearson_ | 0.48 ± 0.05 | 0.49 ± 0.10 | 0.35 ± 0.11 | 0.45 ± 0.13 | 0.52 ± 0.03 | 0.50 ± 0.07 |
|  | r_spearman_ | 0.46 ± 0.05 | 0.44 ± 0.11 | 0.32 ± 0.08 | 0.49 ± 0.08 | 0.48 ± 0.03 | 0.49 ± 0.06 |
|  | RMSE | 23.92 ± 3.35 | 9.66 ± 3.34 | 3.34 ± 1.20 | 2.05 ± 0.18 | 16.50 ± 1.61 | 16.48 ± 0.9 |
|  | slope | 1.15 ± 0.08 | 0.99 ± 0.08 | 0.97 ± 0.08 | 1.07 ± 0.05 | 1.04 ± 0.03 | 1.03 ± 0.08 |
| Stack | r_pearson_ | 0.49 ± 0.04 | 0.50 ± 0.08 | 0.36 ± 0.08 | 0.47 ± 0.05 | 0.53 ± 0.02 | 0.53 ± 0.04 |
|  | r_spearman_ | 0.48 ± 0.04 | 0.48 ± 0.05 | 0.33 ± 0.05 | 0.53 ± 0.05 | 0.51 ± 0.02 | 0.50 ± 0.03 |
|  | RMSE | 19.76 ± 2.10 | 9.16 ± 3.04 | 3.03 ± 1.04 | 2.03 ± 0.15 | 13.63 ± 0.79 | 14.04 ± 0.87 |
|  | slope | 0.96 ± 0.08 | 1.09 ± 0.06 | 0.06 ± 1.05 | 1.08 ± 0.05 | 0.94 ± 0.08 | 0.95 ± 0.06 |

r_pearson_ – Pearson correlation; r_spearman_ – Spearman rank correlation; RMSE – root mean square. AST - aspartate aminotransferase; GGT - γ-glutamyl transferase; BILt - total bilirubin; ALP - alkaline phosphatase and PON – Paraoxonase.

**Table S17** Prediction fit parameters, including standard errors, considering the top 1500 SNP markers ranked by a GBM, obtained from genomic BLUP (GBLUP), Bayesian B (BayesB), elastic net (ENET), gradient boosting machine (GBM) and stacking ensemble (Stack) for oxidative stress metabolites.

| **Model** | **Fit parameter** | **ROMt** | **AOPP** | **FRAP** | **SHp** |
| --- | --- | --- | --- | --- | --- |
| Gblup | r_pearson_ | 0.40 ± 0.079 | 0.56 ± 0.043 | 0.26 ± 0.057 | 0.53 ± 0.021 |
|  | r_spearman_ | 0.39 ± 0.050 | 0.45 ± 0.043 | 0.26 ± 0.036 | 0.50 ± 0.029 |
|  | RMSE | 2.41 ± 0.436 | 6.36 ± 0.521 | 24.36 ± 3.039 | 35.85 ± 4.293 |
|  | slope | 0.92 ± 0.078 | 0.99 ± 0.077 | 0.94 ± 0.066 | 1.03 ± 0.060 |
| EM | r_pearson_ | 0.41 ± 0.042 | 0.56 ± 0.057 | 0.27 ± 0.079 | 0.52 ± 0.019 |
|  | r_spearman_ | 0.43 ± 0.039 | 0.44 ± 0.044 | 0.27 ± 0.036 | 0.50 ± 0.044 |
|  | RMSE | 1.96 ± 0.390 | 6.92 ± 0.555 | 30.33 ± 4.119 | 36.38 ± 2.027 |
|  | slope | 0.98 ± 0.077 | 0.98 ± 0.052 | 0.91 ± 0.033 | 1.02 ± 0.062 |
| GBM | r_pearson_ | 0.41 ± 0.07 | 0.53 ± 0.044 | 0.26 ± 0.066 | 0.52 ± 0.028 |
|  | r_spearman_ | 0.42 ± 0.086 | 0.36 ± 0.091 | 0.24 ± 0.089 | 0.49 ± 0.016 |
|  | RMSE | 2.03 ± 0.721 | 6.87 ± 0.826 | 32.64 ± 3.826 | 38.39 ± 2.757 |
|  | slope | 1.03 ± 0.08 | 1.28 ± 0.078 | 0.94 ± 0.078 | 1.08 ± 0.066 |
| Stack | r_pearson_ | 0.42 ± 0.042 | 0.57 ± 0.052 | 0.28 ± 0.052 | 0.54 ± 0.019 |
|  | r_spearman_ | 0.43 ± 0.040 | 0.46 ± 0.039 | 0.28 ± 0.039 | 0.53 ± 0.041 |
|  | RMSE | 1.97 ± 0.396 | 6.36 ± 0.470 | 0.47 ± 29.65 | 35.52 ± 1.475 |
|  | slope | 0.93 ± 0.073 | 0.94 ± 0.066 | 0.066 ± 0.94 | 1.05 ± 0.055 |

r_pearson_ – Pearson correlation; r_spearman_ – Spearman rank correlation; RMSE – root mean square. ROMt - total reactive oxygen metabolites; AOPP - advanced oxidation protein products; FRAP - ferric reducing antioxidant power; SHp - thiol groups.

**Table S18** Prediction, fit parameters including standard errors, considering the top 1500 SNP markers ranked by a GBM, obtained from genomic BLUP (GBLUP), Bayesian B (BayesB), elastic net (ENET), gradient boosting machine (GBM), and stacking ensemble (Stack) for Inflammation/innate immunity.

| **Model** | **Fit parameter** | **Ceruloplasmin** | **PROTt** | **Globulins** | **Haptoglobin** | **Myeloperoxidase** |
| --- | --- | --- | --- | --- | --- | --- |
| Gblup | r_pearson_ | 0.43 ± 0.083 | 0.35 ± 0.055 | 0.31 ± 0.036 | 0.17 ± 0.011 | 0.4 ± 0.07 |
|  | r_spearman_ | 0.30 ± 0.053 | 0.35 ± 0.026 | 0.32 ± 0.033 | 0.14 ± 0.077 | 0.38 ± 0.043 |
|  | RMSE | 0.68 ± 0.031 | 4.79 ± 0.507 | 5.52 ± 0.459 | 0.24 ± 0.023 | 75.09 ± 10.514 |
|  | slope | 0.97 ± 0.068 | 0.99 ± 0.016 | 1.03 ± 0.071 | 0.97 ± 0.011 | 0.98 ± 0.018 |
| BayesB | r_pearson_ | 0.47 ± 0.078 | 0.37 ± 0.057 | 0.33 ± 0.033 | 0.20 ± 0.047 | 0.42 ± 0.09 |
|  | r_spearman_ | 0.32 ± 0.081 | 0.36 ± 0.025 | 0.33 ± 0.036 | 0.17 ± 0.062 | 0.39 ± 0.038 |
|  | RMSE | 0.67 ± 0.031 | 4.74 ± 0.487 | 5.43 ± 0.501 | 0.23 ± 0.022 | 74.39 ± 8.789 |
|  | slope | 0.95 ± 0.059 | 0.99 ± 0.025 | 1.01 ± 0.043 | 1.05 ± 0.052 | 0.98 ± 0.063 |
| EM | r_pearson_ | 0.51 ± 0.032 | 0.39 ± 0.056 | 0.34 ± 0.021 | 0.21 ± 0.030 | 0.43 ± 0.060 |
|  | r_spearman_ | 0.43 ± 0.091 | 0.37 ± 0.04 | 0.34 ± 0.025 | 0.19 ± 0.079 | 0.44 ± 0.039 |
|  | RMSE | 0.66 ± 0.052 | 4.60 ± 0.466 | 5.35 ± 0.466 | 0.24 ± 0.024 | 73.32 ± 9.594 |
|  | slope | 0.98 ± 0.025 | 1.01 ± 0.018 | 1.02 ± 0.023 | 1.01 ± 0.044 | 0.98 ± 0.012 |
| GBM | r_pearson_ | 0.53 ± 0.029 | 0.41 ± 0.060 | 0.37 ± 0.021 | 0.22 ± 0.029 | 0.45 ± 0.050 |
|  | r_spearman_ | 0.51 ± 0.043 | 0.39 ± 0.025 | 0.36 ± 0.028 | 0.20 ± 0.067 | 0.45 ± 0.038 |
|  | RMSE | 0.65 ± 0.054 | 4.58 ± 0.449 | 5.27 ± 0.460 | 0.24 ± 0.022 | 72.55 ± 9.22 |
|  | slope | 1.01 ± 0.012 | 1.01 ± 0.012 | 1.03 ± 0.035 | 0.97 ± 0.039 | 1.02 ± 0.018 |
| Stack | r_pearson_ | 0.55 ± 0.030 | 0.43 ± 0.058 | 0.38 ± 0.024 | 0.24 ± 0.028 | 0.47 ± 0.03 |
|  | r_spearman_ | 0.52 ± 0.047 | 0.40 ± 0.039 | 0.37 ± 0.024 | 0.23 ± 0.029 | 0.46 ± 0.038 |
|  | RMSE | 0.64 ± 0.044 | 4.52 ± 0.430 | 5.20 ± 0.459 | 0.23 ± 0.024 | 71.58 ± 9.253 |
|  | slope | 1.03 ± 0.056 | 0.99 ± 0.011 | 1.03 ± 0.035 | 1.07 ± 0.081 | 1.02 ± 0.015 |

r_pearson_ – Pearson correlation; r_spearman_ – Spearman rank correlation; RMSE – root mean square. PROTt – total proteins.

**Table S19** Prediction fit parameters, including standard errors, considering the top 1500 SNP markers ranked by a GBM, obtained from genomic BLUP (GBLUP), Bayesian B (BayesB), elastic net (ENET), gradient boosting machine (GBM), and stacking ensemble (Stack) for Minerals in blood.

| **Model** | **Fit parameter** | **Calcium** | **Phosphorus** | **Magnesium** | **Sodium** | **Potassium** | **Chlorine** | **Zinc** |
| --- | --- | --- | --- | --- | --- | --- | --- | --- |
| Gblup | r_pearson_ | 0.40 ± 0.03 | 0.39 ± 0.03 | 0.42 ± 0.06 | 0.38 ± 0.017 | 0.41 ± 0.02 | 0.53 ± 0.020 | 0.44 ± 0.045 |
|  | r_spearman_ | 0.39 ± 0.02 | 0.38 ± 0.03 | 0.39 ± 0.05 | 0.37 ± 0.015 | 0.40 ± 0.020 | 0.51 ± 0.020 | 0.40 ± 0.050 |
|  | RMSE | 0.06 ± 0.01 | 0.17 ± 0.01 | 0.06 ± 0.05 | 1.83 ± 0.08 | 0.20 ± 0.010 | 1.27 ± 0.125 | 2.04 ± 0.20 |
|  | slope | 0.99 ± 0.07 | 0.99 ± 0.06 | 0.99 ± 0.12 | 0.98 ± 0.03 | 1.01 ± 0.07 | 0.99 ± 0.055 | 0.97 ± 0.081 |
| EN | r_pearson_ | 0.41 ± 0.04 | 0.4 ± 0.041 | 0.39 ± 0.025 | 0.42 ± 0.038 | 0.42 ± 0.022 | 0.55 ± 0.022 | 0.46 ± 0.06 |
|  | r_spearman_ | 0.39 ± 0.031 | 0.4 ± 0.06 | 0.38 ± 0.018 | 0.44 ± 0.035 | 0.39 ± 0.023 | 0.54 ± 0.045 | 0.44 ± 0.038 |
|  | RMSE | 0.05 ± 0.019 | 0.18 ± 0.056 | 0.06 ± 0.012 | 2.06 ± 0.088 | 0.17 ± 0.060 | 2.02 ± 0.065 | 2.08 ± 0.877 |
|  | slope | 1.06 ± 0.014 | 0.98 ± 0.085 | 1.09 ± 0.033 | 1.035 ± 0.078 | 1.033 ± 0.09 | 1.036 ± 0.096 | 0.99 ± 0.038 |
| GBM | r_pearson_ | 0.40 ± 0.029 | 0.41 ± 0.045 | 0.40 ± 0.021 | 0.43 ± 0.025 | 0.40 ± 0.017 | 0.56 ± 0.023 | 0.44 ± 0.033 |
|  | r_spearman_ | 0.39 ± 0.04 | 0.41 ± 0.05 | 0.41 ± 0.023 | 0.44 ± 0.022 | 0.39 ± 0.025 | 0.55 ± 0.028 | 0.4 ± 0.029 |
|  | RMSE | 0.04 ± 0.025 | 0.11 ± 0.035 | 0.05 ± 0.012 | 2.01 ± 0.688 | 0.19 ± 0.028 | 2.07 ± 0.076 | 2.48 ± 0.25 |
|  | slope | 1.021 ± 0.069 | 1.07 ± 0.085 | 1.014 ± 0.08 | 1.08 ± 0.079 | 1.034 ± 0.058 | 1.013 ± 0.05 | 1.07 ± 0.055 |
| Stack | r_pearson_ | 0.42 ± 0.038 | 0.43 ± 0.068 | 0.41 ± 0.023 | 0.46 ± 0.020 | 0.43 ± 0.013 | 0.57 ± 0.025 | 0.47 ± 0.057 |
|  | r_spearman_ | 0.44 ± 0.275 | 0.42 ± 0.064 | 0.40 ± 0.024 | 0.47 ± 0.017 | 0.44 ± 0.019 | 0.56 ± 0.033 | 0.45 ± 0.029 |
|  | RMSE | 0.03 ± 0.024 | 0.26 ± 0.055 | 0.05 ± 0.013 | 1.02 ± 0.332 | 0.36 ± 0.041 | 2.05 ± 0.031 | 2.06 ± 0.293 |
|  | slope | 1.09 ± 0.019 | 1.07 ± 0.098 | 0.99 ± 0.047 | 0.98 ± 0.066 | 1.06 ± 0.04 | 0.98 ± 0.066 | 0.97 ± 0.049 |

r_pearson_ – Pearson correlation; r_spearman_ – Spearman rank correlation; RMSE – root mean square.
